# Two *Theobroma cacao* genotypes with contrasting pathogen tolerance show aberrant transcriptional and ROS responses after salicylic acid treatment

**DOI:** 10.1093/jxb/erv334

**Published:** 2015-07-10

**Authors:** Andrew S. Fister, Shawn T. O’Neil, Zi Shi, Yufan Zhang, Brett M. Tyler, Mark J. Guiltinan, Siela N. Maximova

**Affiliations:** ^1^The Huck Institutes of the Life Sciences, The Pennsylvania State University, University Park, PA 16802, USA; ^2^Center for Genome Research and Biocomputing, Oregon State University, Corvallis, OR 97331, USA; ^3^Center for Applied Genetic Technologies, University of Georgia, Athens, GA 30602, USA; ^4^Department of Botany and Plant Pathology, Oregon State University, Corvallis, OR 97331, USA; ^5^The Department of Plant Science, The Pennsylvania State University, University Park, PA 16802, USA

**Keywords:** Cacao, pathogenesis-related genes, *Phytophthora tropicalis*, reactive oxygen species, salicylic acid, TcNPR1.

## Abstract

Understanding the genetic basis of pathogen susceptibility in various crop plants is crucial to increasing the stability of food, feed, and fuel production. Varietal differences in defence responses provide insights into the mechanisms of resistance and are a key resource for plant breeders. To explore the role of salicylic acid in the regulation of defence in cacao, we demonstrated that SA treatment decreased susceptibility to a pod rot pathogen, *Phytophthora tropicalis* in two genotypes, Scavina 6 and Imperial College Selection 1, which differ in their resistance to several agriculturally important pathogens. Transient overexpression of TcNPR1, a major transcriptional regulator of the SA-dependent plant immune system, also increased pathogen tolerance in cacao leaves. To explore further the genetic basis of resistance in cacao, we used microarrays to measure gene expression profiles after salicylic acid (SA) treatment in these two cacao genotypes. The two genotypes displayed distinct transcriptional responses to SA. Unexpectedly, the expression profile of the susceptible genotype ICS1 included a larger number of pathogenesis-related genes that were induced by SA at 24h after treatment, whereas genes encoding many chloroplast and mitochondrial proteins implicated in reactive oxygen species production were up-regulated in the resistant genotype, Sca6. Sca6 accumulated significantly more superoxide at 24h after treatment of leaves with SA. These experiments revealed critical insights regarding the molecular differences between cacao varieties, which will allow a better understanding of defence mechanisms to help guide breeding programmes.

## Highlight

Genotype specific strategies for plant defence: the Sca6 genotype of *Theobroma cacao* favors the accumulation of reactive oxygen species, while the ICS1 variety has a stronger PR gene transcriptional response.

## Introduction

*Theobroma cacao* (cacao), the seeds of which are used to make chocolate, is an economically important crop providing income to small-scale farmers in tropical regions all over the world (Wood and Lass, 2008). However, ∼30−40% of annual cacao production is lost to pathogens due to its very high disease susceptibility (Hebbar, 2007; Argout *et al.*, 2008). Cacao is the host to several diseases including witches’ broom disease (WBD), caused by *Moniliophthora perniciosa* (Purdy and Schmidt, 1996), frosty pod rot caused by *Moniliophthora roreri* (Phillips-Mora and Wilkinson, 2007), and black pod rot caused by several *Phytophthora* species (Bailey *et al.*, 2005*a*). Two genotypes of cacao, Scavina 6 (Sca6) and Imperial College Selection 1 (ICS1), are of special importance to the study of cacao disease resistance because they differ in their tolerance to the above-mentioned pathogens; Sca6 is a more resistant variety and ICS1 is highly susceptible (Yamada and Lopes, 1999; Brown *et al.*, 2005; Faleiro *et al.*, 2006). Several quantitative trait loci (QTLs) have been mapped for resistance to WBD and black pod rot in Sca6; however, the mechanistic differences underlying the variation in susceptibility between these two varieties are still unclear (Risterucci *et al.*, 2003; Brown *et al.*, 2005; Faleiro *et al.*, 2006). A fuller understanding of the genes associated with susceptible and resistance responses would be extremely useful for cacao breeding programmes and the selection of new varieties with higher resistance.

Salicylic acid (SA) is considered to be the most important signalling hormone controlling the responses to biotrophic and hemibiotrophic pathogens in other plant species (Shah, 2003; Durrant and Dong, 2004; Loake and Grant, 2007; Vlot *et al.*, 2008; Fu and Dong, 2013). Hundreds of genes induced by SA have been isolated and characterized in the model plant *Arabidopsis thaliana* (Arabidopsis) (Cao *et al.*, 1994; Dong, 2004; Uquillas *et al.*, 2004; Grant and Lamb, 2006; Wang *et al.*, 2006; Lee *et al*., 2007, 2009; Loake and Grant, 2007; Attaran *et al.*, 2009). In SA-treated unripe pepper fruit, 177 of 7900 cDNA clones exhibited more than 4-fold transcript accumulation (Lee *et al.*, 2009). In rice, microarray analysis identified SA-inducible WRKY transcription factors involved in rice blast resistance (Shimono *et al.*, 2007). Aside from its role in pathogenesis-related (PR) gene induction, SA is also involved in the oxidative burst and hypersensitive response during pathogen attack (Alvarez, 2000; Torres *et al.*, 2006). SA synthesis and reactive oxygen species (ROS) production are believed to act in a positive feedback loop with each other and together lead to induction of programmed cell death (Overmyer *et al.*, 2003). The oxidative burst and cell death are known to involve ROS production both in the apoplast as well as in intracellular compartments, including mitochondria and chloroplasts (Vlot *et al.*, 2009; O’Brien *et al.*, 2012).

Recent studies have indicated that proteins in the non-expressor of pathogenesis-related (NPR) family are the receptors for SA (Fu *et al.*, 2012; Wu *et al.*, 2012). Two groups have demonstrated that NPR3 and NPR4 are members of a receptor complex for NPR1, mediating an interaction between it and CUL3 E3 ligase (Fu *et al.*, 2012), and that cysteine residues in NPR1 are necessary for direct interaction between the protein and SA (Wu *et al.*, 2012). Taken together, these results suggest that there may be some partial redundancy within this family enabling each to interact directly with SA. Even without its potential role as a direct receptor for SA, the importance of NPR1 in regulating the transcriptional changes associated with systemic acquired resistance has been a highly active area of research (Fu and Dong, 2013).

Recent evidence suggests that *T. cacao* also uses the SA-dependent pathway during defence responses (Borrone *et al.*, 2004; Bailey *et al*., 2005*a*, *b*; Maximova *et al.*, 2006; Gesteira *et al.*, 2007), and PR genes are up-regulated in leaves after treatment with the SA analogue BTH (Verica *et al.*, 2004). Moreover, genes encoding cacao homologues of NPR1 (Tc09_g007660) and NPR3 (Tc06_g011480) can partially restore the Arabidopsis *npr1* and *npr3* mutant phenotypes, demonstrating the highly conserved nature of this signalling pathway (Shi *et al*., 2010, 2013). In contrast, the transcriptional responses of *Theobroma cacao* cultivar ‘Comum’ to infection by WBD did not include significant changes in the transcription of genes in the SA pathway although there was activation of a variety of other genes implicated in defence responses and repression of photosynthesis (Teixeira *et al.*, 2014), implying that the SA pathway may not be the predominant mechanism of response to this particular pathogen or in this cultivar. To explore the mechanisms potentially responsible for genotype-specific differences in defence responses in cacao, we used a custom cacao microarray to evaluate differential gene expression in cacao leaves in response to SA treatment in the Sca6 and ICS1 genotypes. Our results uncovered distinct differences between the two genotypes in accumulation of ROS in SA treated leaves that were consistent with specific differences in gene expression, suggesting that these mechanisms may play key roles in determining disease susceptibility in cacao.

## Materials and methods

### Leaf disk pathogen bioassay using cacao leaves

Sca6 genotype is known to be more resistant to a number of pathogens, and genotype ICS1 is considered to be highly susceptible (Risterucci *et al.*, 2003; Faleiro *et al.*, 2006). Thus we utilized these two genotypes to study the molecular mechanisms of defence response in cacao. A leaf inoculation assay was performed with *Phytophthora tropicalis* to verify and quantify the differences between ICS1 and Sca6 in their response to treatment with SA. Fully-expanded, light green, and supple leaves at developmental stage C (Mejia *et al.*, 2012) on greenhouse-grown trees of both genotypes were treated with 1mM SA or water (as a control). Twenty-four hours after treatment, the leaves were harvested from the plants and inoculated with mycelial plugs of *Phytophthora tropicalis* as previously described (Mejia *et al.*, 2012). Eight leaf pieces from each genotype and each treatment were inoculated and photographs were taken 72h post-inoculation with a 1/30s exposure time, aperture of f=5.6, using a Nikon D90 equipped with a Nikon AF-S NIKKOR DX 18−135mm lens. Lesion sizes were measured using ImageJ. Average lesion sizes were calculated from 24 replicates and significance was determined by single factor ANOVA. As a complementary measurement of pathogen virulence, the relative amount of pathogen DNA was measured by determining the ratio of *Phytophthora* DNA to cacao DNA in infection zones by qPCR. Lesions were collected using a 2cm diameter cork borer surrounding the inoculation site and genomic DNA was extracted using a Tissumizer (Tekmar, Mason, Ohio, USA) and DNeasy plant mini kit (Qiagen). Specific primers for *P. tropicalis Actin* (F: GACAACGGCTCCGGTATGTGCAAGG and R: GTCAGCACACCACGCTTGGACTG) and cacao *Actin*7 (Tc01g010900) (F: AGGTGGAGATCATTGAAGGAGGGT and R: ACCAGCGGTCATCACAAGTCACAA) genes were used as pathogen and host targets. qPCR was performed using an ABI 7300 (Applied Biosystems, Foster City, CA, USA) as previously described (Shi *et al.*, 2013). Differences between genotypes and treatments were identified using Fisher’s partial least-squares difference analysis.

### Transient *Agrobacterium*-mediated transformation of cacao leaves

To create a T-DNA binary vector for overexpression of the *TcNPR1* coding sequence, plasmid pGZ12.0106 (GenBank: KP844566) was digested with restriction enzymes SpeI and HpaI and then was ligated to a DNA fragment containing the *TcNPR1* coding sequence isolated as a Spe I-Pvu II restriction fragment generated by digesting plasmid pGEM-TcNPR1 (Shi *et al.*, 2010) resulting in pGS12.0224 (GenBank: KP844565). The T-DNA region of the vector contains the modified CaMV-35S derivative, E12-Ω promoter (Mitsuhara *et al.*, 1996), which drives *TcNPR1, EGFP* (Clontech), and *NPTII-A* (De Block *et al.*, 1984) transgenes, and these are followed by the 35S terminator. A second copy of the *NPTII* marker gene (*NPTII-B*), is flanked by the NOS promoter and terminator (Lichtenstein and Fuller, 1987). The vector map for pGS12.0224 was created in Geneious (Drummond *et al.*, 2012), and is shown in Supplementary Fig. S1. The pGS12.0224 vector and the control vector (pGH00.0126, GenBank: KF018696) were used to transiently transform cacao leaf tissue using *Agrobacterium tumefaciens* vacuum infiltration as previously described (Shi *et al.*, 2013). Forty-eight hours after infiltration, leaves were screened with a fluorescent stereo-microscope equipped with an EGFP filter system as previously described (Maximova *et al.*, 1998). Leaves exhibiting green fluorescence over 90% of their area were used in *P. tropicalis* infection assays. Inoculation was performed as described above. Disease impact was determined using lesion size analysis and qPCR as described above. Tissue surrounding the lesions was collected and used for RNA extractions and subsequent qRT-PCR to verify transgene expression. RNA from each sample were isolated as previously described (Verica *et al.*, 2004). qRT-PCR was performed using the Taqman ABI 7300 Sequence Detection System (Applied Biosystems Inc, Foster City, CA, USA). Primer and probe sequences for qRT-PCR were: *TcNPR1*: 5ʹ—GTCACGTGCTGTCTGACCTTGT, 3ʹ—TCACAGTTCATAATCTGGTCGAGC, Probe—TYCCGCGCT GTTCGGCAGT; *TcActin*: 5ʹ—GATTCAGATGCCCAGAAGT CTTG, 3ʹ—TCTCGTGGATTCCAGCAGCT, Probe—CCAGCCC TCGTTGTGGGAAAGG; *TcUbiquitin*: 5ʹ —AGGCCTCAACTG GTTGCTGT, 3ʹ—ACCGGCAAGACCATCACTCT, Probe— CGAGAGCAGCGACACCCATCGACA. The qRT-PCR normalization and analysis were performed using REST software (Pfaffl *et al.*, 2002) as previously described (Mejía *et al.*, 2014).

### Plant treatment and RNA extraction

Three- to four-month-old cacao rooted cuttings of genotypes ICS1 and Sca6 were treated with 2mM SA dissolved in water under greenhouse conditions as previously described (Swanson *et al.*, 2008). Plants treated with only water served as negative controls. Twenty-four hours after treatment, leaf samples from different developmental stages A, C and E (Mejia *et al.*, 2012) were collected. Three biological replicates were collected for each genotype. Thus, 36 samples were collected in total. RNAs from each sample were isolated as previously described (Verica *et al.*, 2004).

### Microarray analysis

Roche Nimblegen oligonucleotide custom *T. cacao* gene expression 4×72 k (four arrays of 72 000 probes) were manufactured (*T. cacao* 17K microarray, design ID 7114 manufactured by Roche). Each array contained four probes of 50–60 mers in length for each of 17 247 unigenes. Three biological replicates were collected for each genotype, each treatment and each developmental stage, except for Stage A Sca6 and Stage E ICS1, both of which had only two replicates per treatment. Array design, RNA extraction protocol, hybridization procedures, scanning and data normalization protocols are described in Mejía *et al*. (2014). Data from the microarray experiment are available at the NCBI Gene Expression Omnibus (GEO: GPL18260).

### Statistical analysis of microarray results

After log_2_ transformation of expression data, probe sets with mean log_2_ expression less than the background level of 8.0 were removed. Differential expression between genotypes on per-treatment and per-treatment+per-leaf-stage bases were assessed by general linear hypothesis (GLH) tests via the R multcomp package version 1.3 (Bretz *et al.*, 2010). Differential expression between treatments was assessed on a per-genotype+per-leaf-stage basis with two-sided student’s *t*-tests on expression differences (testing whether mean differential expression equaled 0) between replicates paired by sampling day per group. Differential expression between treatments overall and on per-genotype bases were assessed with GLH tests. On the microarrays, a number of genes were represented by additional probe sets generated from 3ʹ UTR regions. For those genes with multiple probe sets that passed expression cutoffs, a mixed linear model treating probe set as a random factor was used for the GLH tests rather than a simple linear model. All *P*-values were adjusted using the Benjamini-Hochberg procedure on a per-test basis (Benjamini and Hochberg, 1995).

### Identification of *T. cacao* PR genes

PR genes in the *T. cacao* Criollo genome were identified according to the protocol described in Campos *et al.* (2007). The amino acid sequence for each PR gene type member was compared to a database of cacao polypeptide sequences using BLASTp with an e-value cutoff of e<10^–5^.

### GO enrichment analysis of microarray data

The probe set on the microarray was annotated with best hits from Blast searches of the Arabidopsis genome as previously described (Mejía *et al.*, 2014). All genes with available *A. thaliana* loci accession numbers were classified according to gene ontology (GO) terms using the tools for GO annotations at The Arabidopsis Information Resource (http://arabidopsis.org/tools/bulk/go/index.jsp). Gene Ontology enrichment analysis was performed using the Parametric Analysis of Gene Enrichment (Kim and Volsky, 2005) module on agriGO (http://bioinfo.cau.edu.cn/agriGO/) (Du *et al.*, 2010). For any given comparison, all genes on the microarray with annotated Arabidopsis best hits and statistically significant (Benjamini-Hochberg *P*<0.05) differential regulation were included in the analysis.

### qRT-PCR measurement of selected genes

qRT-PCR analyses were performed on the same RNA samples produced for the microarray experiment. One microgram of RNA from each sample was reverse transcribed by M MuLV Reverse Transcriptase (New England Biolabs) using oligo-(dT)15 as a primer to generate cDNA. qRT-PCR was performed in 10 µl reactions, consisting of 5 µl of SYBR Green PCR Master Mix (Takara), 0.2 µl of Rox, and 0.4 µl of each primer, diluted to 5 µM, and 4 µl of cDNA. Each reaction was performed in technical duplicate using the Applied Biosystem Step One Plus Realtime PCR System (Roche) with the following programme: 15min at 94°C, 40 cycles of 15 s at 94°C, 20 s at 60°C and 40 s at 72°C. The specificity of the primer pairs were examined using a dissociation curve and by visualization on 2% agarose gels. A cacao *Actin* gene was used as a reference (Shi *et al.*, 2010), and fold change was calculated using the ΔΔC_T_ method (Livak and Schmittgen, 2001).

### ROS quantification by peroxide and superoxide staining

To test whether accumulation of ROS differed between the two genotypes, we performed nitroblue tetrazolium (NBT) and 3,3′-diaminobenzidine (DAB) staining to quantify the accumulation of superoxide and peroxide, respectively. Greenhouse-grown mature (flowering) ICS1 and Sca6 trees were sprayed with 2mM SA or with water as a control. Twenty-four hours after treatment, stage C leaves were removed from the trees, discs were punched out using a 1.5cm diameter cork borer and randomized into groups of three for infiltration with either 1% NBT solution in 10mM potassium phosphate buffer or 1mg/ml DAB following a published protocol (Daudi *et al.*, 2012). Leaves were vacuum infiltrated for 5min three times, with pressure reaching −23 in.-Hg. After infiltration, NBT-treated discs were bathed in NBT solution in darkness for 2h on an orbital shaker (60rpm) and DAB-treated samples were bathed in DAB solution in darkness for 8h on an orbital shaker (60rpm). After incubation, chlorophyll was bleached by soaking leaf discs in a 3:1:1 ethanol, glycerol, acetic acid mixture for 45min with periodic vortexing for 5 s. Leaf discs were placed back in their original petri dishes, but sandwiched between the lid and the base to flatten them. Leaf discs were photographed as described above. Using ImageJ, NBT-stained areas were calculated by selecting blue tissue using a colour threshold, passing red=0−160, green=0−160, and blue=0−255. Area of staining was averaged across the three discs on a plate, and to reflect darker stains with a larger number, this value was multiplied by 255 minus the mean grey value within the area selected by the colour threshold. DAB staining also was detected by using a colour threshold to select brown tissue, passing red=0−160, green=0−150 and blue=0−90. The area of staining for each biological replicate was multiplied by 255 minus the measured mean grey value. Differences between genotypes and treatments were identified using Fisher’s partial least-squares difference analysis.

## Results

### SA treatment enhances resistance to *P. tropicalis* in both genotypes

**Fig. 1.**
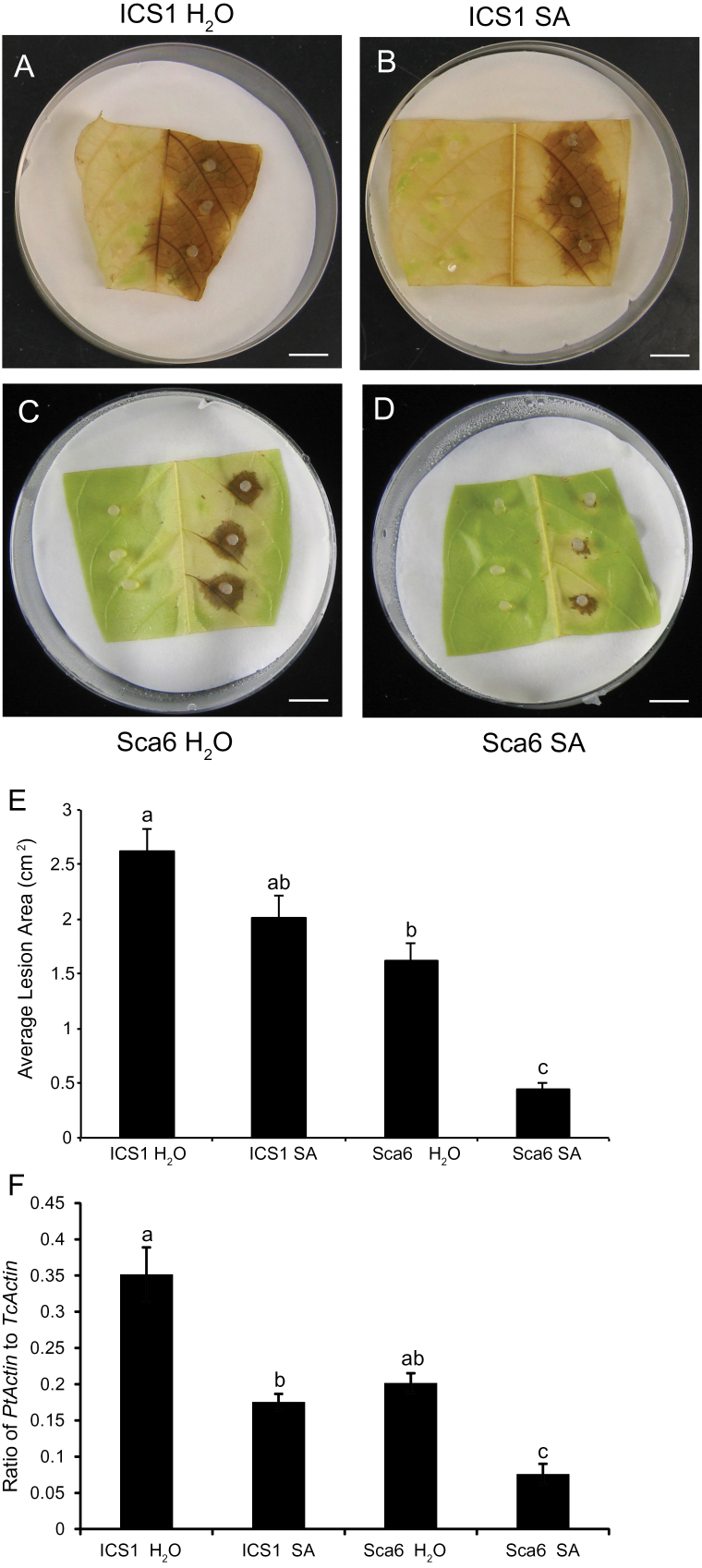
Inoculation of SA pre-treated stage C leaves from ICS1 and SCA6 with *Phytophthora tropicalis*. Stage C leaves were inoculated with agar plugs containing *P. tropicalis* mycelium 24h after water or 1mM SA treatment. Representative images of (A) water-treated ICS1 leaves, (B) SA-treated ICS1 leaves, (C) water-treated SCA6 leaves and (D) SA-treated SCA6 leaves 3 d after inoculation. Scale bars, 1cm. (E) Average lesion areas in replicate leaves were evaluated by ImageJ. Data represent means ±SE of treated leaves from 24 replicates per genotype. Letters above bar chart show the significant differences (*P*<0.05) determined by Fisher’s PLSD analysis. (F) Relative pathogen biomass was measured by qPCR with DNA isolated 48h after inoculation and is expressed as the ratio of *P. tropicalis actin* to cacao *actin*. Bars represent means ±SE of four biological replicates, each with three technical replicates. Letters above the bar show the significant differences (*P*<0.05) determined by Fisher’s PLSD analysis.

At the time of collection, the ICS1 and Sca6 leaves were very similar in appearance and texture, however at the end of the 24h incubation, most of the leaf surfaces of ICS1 became chlorotic, while Sca6 leaves remained green (Fig. 1A−D). This difference was apparent until the end of the pathogen infection period. By 3 d post-inoculation, all SA and water treated leaves of both genotypes developed necrotic lesions at the sites of infection (Fig. 1A−D). Lesion areas were measured using ImageJ software (Schneider *et al.*, 2012). Symptoms were most severe in the water-treated ICS1 leaves. The average lesion areas of SA-treated ICS1 leaves was 20% smaller compared to the water treated ICS1 leaves, though the difference was not significant (Fig. 1E). Treatment of Sca6 leaves with SA resulted in a statistically significant reduction of lesion areas. The lesions of SA treated Sca6 leaves were ~60% smaller than those of Sca6 control leaves and 80% smaller than ICS1 controls. To assess pathogen growth we extracted genomic DNA from the lesions and performed qPCR with *P. tropicalis—*specific primers and primers for the cacao actin gene. SA treatment significantly (*P*<0.05) reduced pathogen growth in both genotypes (Fig. 1F). Pathogen biomass in control Sca6 leaves was similar to SA-treated ICS1, but was further reduced in SA-treated Sca6 tissue. These results confirmed that detached leaves of ICS1 are more susceptible to *P. tropicalis* than Sca6 at both basal and SA-induced states. Both genotypes demonstrated an SA response that resulted in decreased lesion size and pathogen growth, however, the SA effect was greater in Sca6 (4-fold reduction in pathogen biomass accumulation) than in ICS1 (1.7-fold) (Fig. 1E, F).

### Transient overexpression of TcNPR1 enhances resistance to *P. tropicalis*

**Fig. 2.**
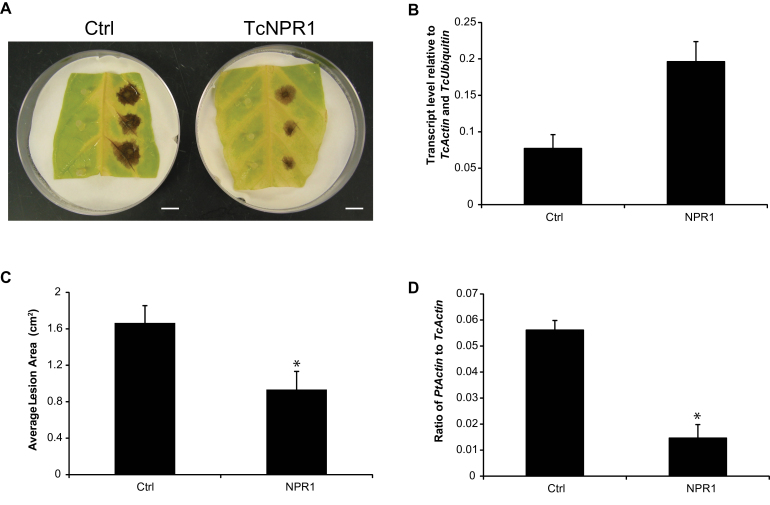
Functional analysis of *TcNPR1*. (A) Representative images of lesions from control and *TcNPR1* transiently transformed leaves 2 d after *Phytophthora tropicalis* inoculation. (B) qRT-PCR analysis of *TcNPR1* transcript 2 d after vacuum infiltration. Ctrl, control; NPR1, *TcNPR1*overexpression. Bars represent means ±SE of three biological replicates. (C) Average lesion areas from control and *TcNPR1* overexpressing leaves were measured 3 d after inoculation using ImageJ. Bars represent the means ±SE of measurements from 12 lesion spots from four leaf discs of each genotype. The asterisk denotes a significant difference determined by single factor ANOVA (*P*<0.05). (D) Pathogen biomass was measured at the lesion sites by qPCR to determine the ratio of pathogen DNA to cacao DNA 2 d after inoculation. Bars represent four biological replicates, each with three technical replicates. The asterisk denotes a significant difference determined by single factor ANOVA analysis (*P*<0.05).

The NPR1 protein is a key regulator of systemic acquired resistance in plants (Fu and Dong, 2013) and our group has previously demonstrated that expression of cacao TcNPR1 was able to partially restore the phenotype of an Arabidopsis *npr1* mutant (Shi *et al.*, 2010). To examine further the involvement of the systemic acquired resistance pathway in the defence response in cacao, we employed transient transformation of cacao Sca6 leaves followed by a pathogen infection assay (Mejia *et al.*, 2012; Shi *et al.*, 2013). Three days after *P. tropicalis* infection, lesions had formed on both control and *TcNPR1* transgenic leaf sections (Fig. 2A). Using qRT-PCR, we demonstrated that expression of *TcNPR1* was increased ~3-fold compared to tissue transformed with a vector control lacking the *TcNPR1* transgene (Fig. 2B). Average lesion area was significantly smaller in *TcNPR1* overexpressing leaves compared to the control vector (Fig. 2C). Additional quantification of *P. tropicalis* DNA in the lesions by qRT-PCR demonstrated that the growth of the pathogen was also significantly reduced in the transformed tissue (Fig. 2D). These results indicated that *TcNPR1* overexpression results in increased pathogen resistance in our *in vitro* assay and further implicates the SA pathway as a major mechanism of resistance in cacao.

### Differential gene expression detected by microarray analysis

**Fig. 3.**
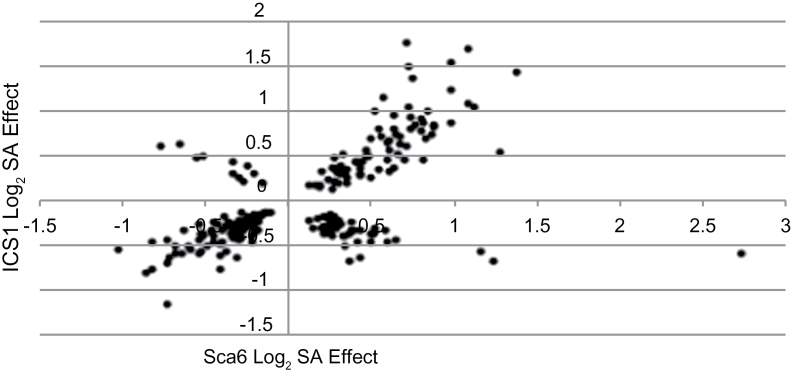
Gene induction differences in Sca6 and ICS1. X-axis represents log_2_ expression change in SA-treated versus water-treated Sca6 leaves, after obtaining the general linear model mean of differences across leaf stages. Y-axis represents log_2_ expression change in SA-treated versus water-treated ICS1 leaves, after obtaining the general linear model mean of differences across leaf stages. Points represent the 234 genes with statistically significant (BH *P*<0.05) expression changes in both genotypes.

To study the responses of the two contrasting genotypes (Sca6 and ICS1) to SA treatment, we used a microarray to measure the transcript levels of over 17 000 cacao genes. By using a general linear model to assess expression differences across leaf stages, 436 and 601 genes were identified as being up- and down-regulated, respectively, in ICS1 (Benjamini-Hochberg (BH) adjusted *P*<0.05) in response to SA (Supplementary Table S1). Although a number of these genes had very low fold changes, they were statistically significant and thus were included in our subsequent analysis. In the Sca6 genotype, 490 and 447 (Supplementary Table S2) genes were detected as up- and down-regulated, respectively (BH-adjusted *P*<0.05) (Table 1). Of all significant genes regulated, 234 genes (Supplementary Table S3) had statistically significant differential regulation in both genotypes. The effect of SA on expression of these genes is plotted in Fig. 3, demonstrating that while many of the genes (173) were regulated in a consistent manner between the genotypes (up- or down-regulated in both genotypes) others (61) responded differently between the genotypes.

**Table 1.** Number of genes up- and down-regulated under SA treatment compared to water treatment for ICS1 and Sca6 genotypes, combining all developmental stages

Genotype	Up	Down
ICS1	436	601
Sca6	490	447

We also compared transcript changes between Sca6 and ICS1 genotypes in basal (water-treated) and induced (SA-treated) states by using a general linear model to assess differences across leaf developmental stages. More than 2000 genes were differentially expressed between the genotypes in both basal and induced states (BH-adjusted *P*<0.05). At the basal state 1124 genes had higher expression in Sca6 than ICS1 and 1121 genes had higher expression ICS1 than Sca6. Similarly, in the induced state we detected 1051 genes with higher expression in Sca6 than ICS1 and 1016 genes with higher expression ICS1 than Sca6.

### Genotypes differ in induction of PR genes

**Fig. 4.**
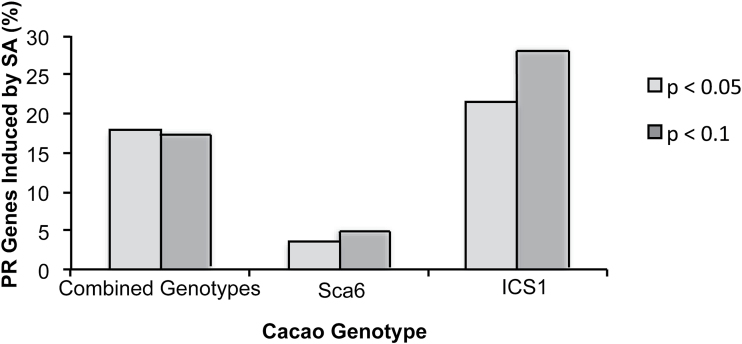
Number of PR genes induced by SA treatment for all leaf stages in Sca6 alone, in ICS1 alone, and across both genotypes. Y-axis shows percentage of PR genes included on the microarray that were induced by SA. Light grey bars represent genes induced with BH *P*<0.05, dark grey bars represent genes induced with BH *P*<0.10.

A hallmark response to SA treatment is the induction of the PR genes, which are families of genes encoding proteins with direct effects on pathogens (Fu and Dong, 2013). Of the 354 PR genes we identified in the *T. cacao* Criollo genome, 136 were represented on the array and 55 passed background normalization (Supplementary Table S4). Unexpectedly, more PR genes were up-regulated by SA in ICS1, the susceptible genotype, than in Sca6 (Fig. 4; cutoffs of BH-adjusted *P*<0.05 and *P*<0.1). At either significance level, only one gene, Tc04_g016440 (a PR-14, putative non-specific lipid transfer protein), was up-regulated in both genotypes, 2.1-fold in Sca6 (BH *P*=0.074) and 4.11-fold in ICS1 (BH *P*=0.005). Of the 16 PR genes induced with BH-adjusted *P*<0.1 in ICS1, seven were class III peroxidase family members (PR-9 family). Conversely, Sca6 had no statistically significantly up-regulated class III peroxidases.

### Functions of most highly up-regulated genes suggest genotype-specificity in response to SA

To further investigate the nature of the response to SA and to identify the functional trends in transcript level changes, we examined the annotations of the genes most highly up-regulated in the two genotypes. The most highly up-regulated genes in Sca6 with available locus IDs for Arabidopsis homologues were encoded in the mitochondrial and chloroplast genomes with functions in the electron transport chains (Supplementary Table S1). Of the 45 most highly up-regulated genes in Sca6, nine are chloroplastic, including subunits of photosystem I and II and RUBISCO, and nine are mitochondrial, several being NADH dehydrogenase subunits. In the ICS1 genotype, none of the 45 most highly up-regulated genes were annotated as being encoded in the chloroplast or mitochondrial genomes (Supplementary Table S2). However, of the top 21 most highly up-regulated genes in ICS1, six encode proteins with predicted functions as PR proteins, including a glucosidase, three different endochitinases, a class III peroxidase, and a non-specific lipid transfer protein, none of which were found to be in the most highly up-regulated genes in Sca6. This suggested that the mechanisms of SA-induced defence response in the two genotypes might differ significantly.

**Fig. 5.**
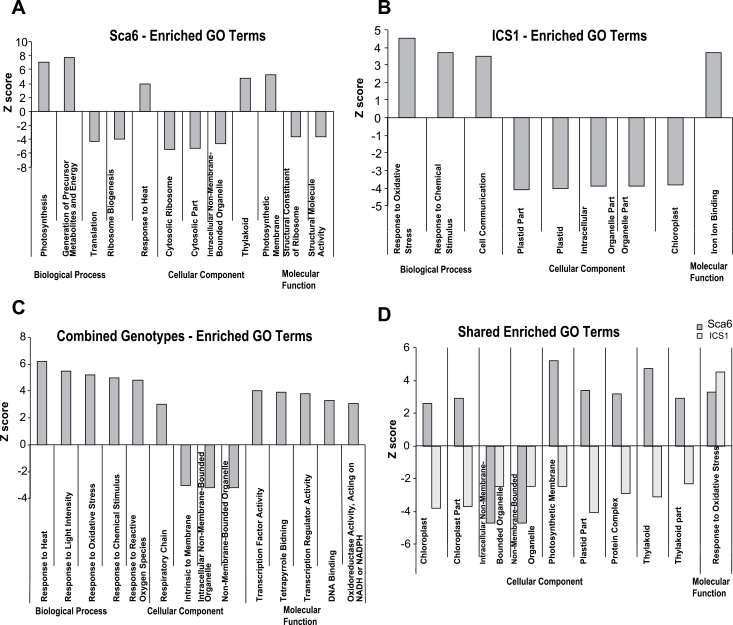
GO enrichment by parametric analysis of gene set enrichment (PAGE). Z scores of select GO terms calculated using PAGE on statistically significantly differentially regulated genes in (A) Sca6, (B) ICS1, and (C) averaging both genotypes across all stages. (D) Z scores of select GO terms with statistically significant enrichment in both genotypes. Detailed data are provided in Supplementary Tables 5−9.

Using locus IDs for Arabidopsis homologues most related to the cacao genes on our microarray, parametric analysis of gene set enrichment (PAGE) was performed using the gene ontology (GO) (Ashburner *et al.*, 2000) annotations of the genes (Kim and Volsky, 2005). This analysis compares the relative proportions of genes represented on the microarray within specific GO annotation classes, with the proportions of the same classes found in sets of up-regulated genes in each comparison performed. This results in the calculation of a Z score that indicates the difference between two experimental groups for a specific annotation class and the statistical significance of that difference. We performed PAGE on three sets of genes: (i) genes significantly up- or down-regulated by SA in the Sca6 genotype, pooling developmental stages (BH-adjusted *P*<0.05) (Supplementary Table S5), (ii) genes significantly up- or down-regulated by SA in the ICS1 genotype, pooling developmental stages (Supplementary Table S6), and (iii) all genes with statistically significant differential regulation by SA after pooling samples from both genotypes and all three developmental stages (Supplementary Table S7). Overall, 37, 34, and 46 GO terms were enriched (Benjamini-Yekutieli adjusted *P*≤0.05) (Benjamini and Yekutieli, 2001) in sets 1, 2 and 3 respectively (Supplementary Tables S5−7). Notably, the enriched categories in sets 1 and 2 differ, highlighting the differences in gene expression profiles between the two genotypes. The majority of categories contributing to the differences are associated with photosynthesis or chloroplastic structures, with up-regulation of the genes in these categories in Sca6 (set 1) and down-regulation in ICS1 (set 2). The most enriched terms in each of the three ontologies are included in Fig. 5.

PAGE analysis was also applied to compare transcript differences between genotypes in their basal and SA induced states (Supplementary Tables S8, 9). Using a general linear model, significant genes were identified (BH-adjusted *P*<0.05) across all leaf stages, for each genotype comparing water-treated Sca6 to water-treated ICS1 (basal state) or SA-treated Sca6 to SA-treated ICS1 (induced state). In the comparison of the basal state of the two genotypes, 39 GO terms were enriched (Supplementary Table S8). Positive Z scores indicate higher expression in Sca6 than in ICS1, and negative Z scores indicate higher expression in ICS1 than in Sca6. Interestingly, several defence-related GO terms, including ‘immune response’ (GO:0006955), ‘response to fungus’ (GO:0009620) and ‘response to chitin’ (GO:0010200), along with ‘cellular respiration’ (GO:0045333) and ‘transcription factor activity’ (GO:0003700), were enriched and had negative Z scores, suggesting that ICS1, despite being more susceptible to disease, expressed more active basal defences. Comparing the induced states, 52 GO terms were enriched (Supplementary Table S9). ‘Response to fungus’ again exhibited a negative Z score, along with ‘response to wounding’ (GO:0009611). The majority of the GO terms with positive Z scores were cellular component terms related to chloroplast or biological process terms related to photosynthesis. As processes of energy generation, particularly the light reactions of photosynthesis and electron transport in the mitochondria are known to produce ROS that could be associated with increases in pathogen resistance (Torres, 2010), this enrichment of chloroplast-related terms may explain the tolerance of Sca6 to a variety of pathogens.

### qRT-PCR validates genotypic differences in gene induction

**Fig. 6.**
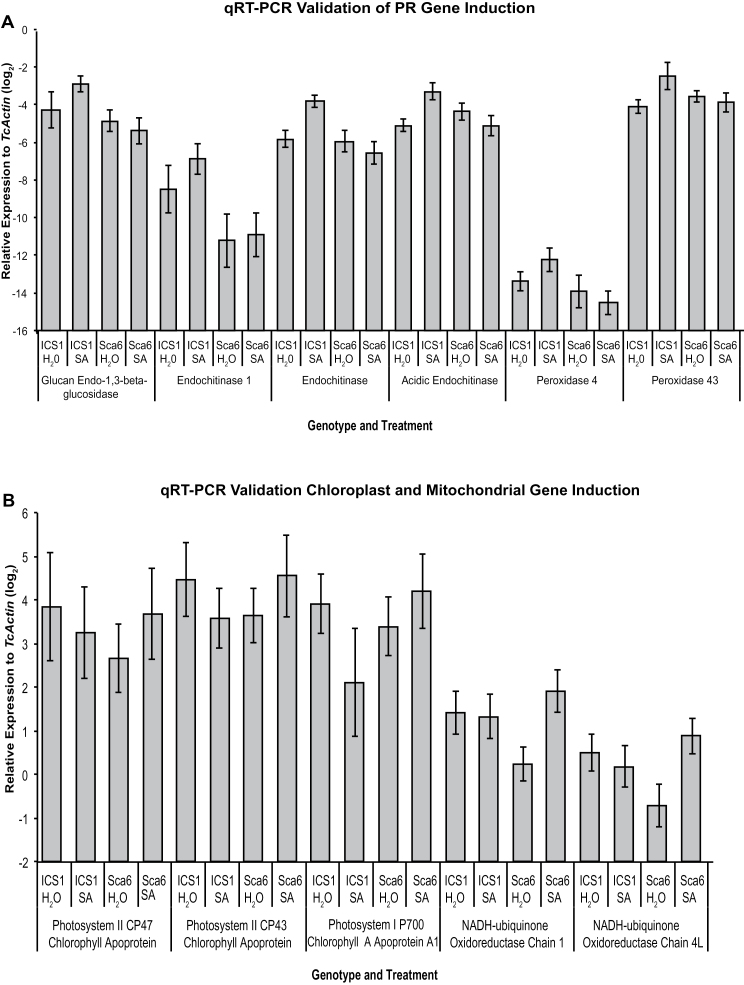
Comparison of transcript levels of select genes from ICS1 and SCA6 genotypes and SA and water (control) treatments as detected by qRT-PCR. Each bar represents the mean of nine samples ±SE (three replicated from each developmental stage). Values are calculated relative to *TcActin*. (A) Effect of SA treatment on cacao PR genes. (B) Effect of SA treatment on cacao genes with Arabidopsis best hits encoded in chloroplasts and mitochondria. Primer sequences are provided in Supplementary Table 10.

qRT-PCR was performed for six selected PR genes to validate the microarray results and obtain more quantitative transcript change measurements. The genes included two class III peroxidases, up-regulated by SA treatment in ICS1, and three chloroplast genes and two mitochondrial genes up-regulated in Sca6 (Fig. 6; Supplementary Table S10). The trends in regulation were consistent between the methods, thus validating the qualitative values from the microarray (Fig. 6A, B). In ICS1, PR genes tended to have higher transcript abundance after SA treatment, while transcript levels for the same genes in Sca6 were more consistent between treatments. Conversely, the three chloroplastic genes showed up-regulation in Sca6 after SA treatment and some down-regulation in ICS1. This is consistent with PAGE analysis resulting in positive Z scores for chloroplast-related GO terms in Sca6 and negative Z scores for the same terms in ICS1. The higher variation observed for the chloroplast genes was due to differences among leaf developmental stages (data not shown). Similarly to the chloroplast genes, the two mitochondrial genes were up-regulated in Sca6, while minor differences were detected in ICS1.

### Sca6 and ICS1 leaf tissues differ in basal and SA induced ROS levels

**Fig. 7.**
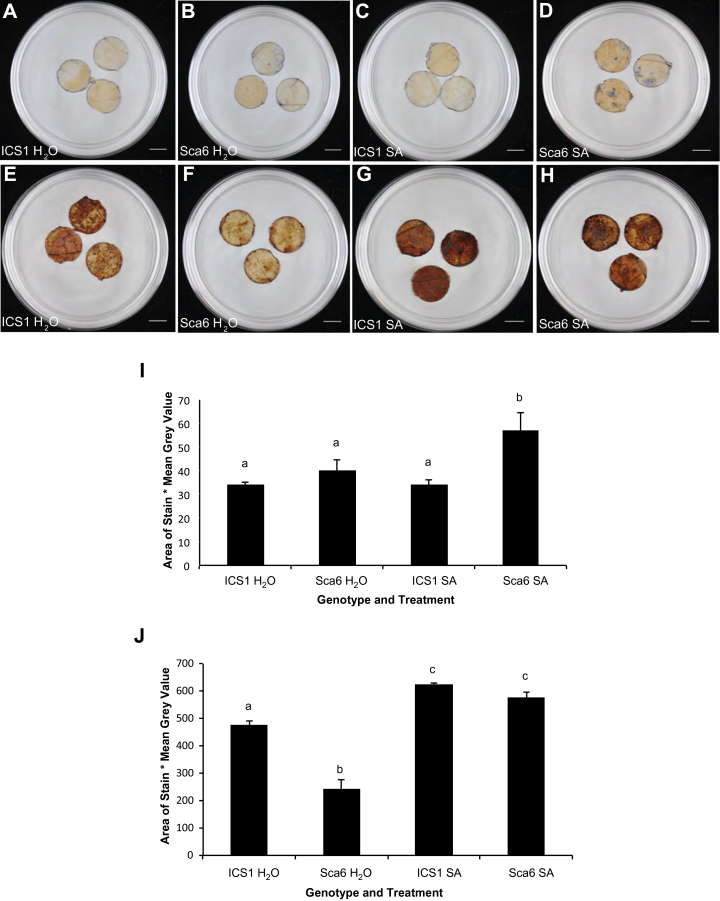
NBT and DAB staining of cacao leaf discs 24h after SA or water treatment. (A) NBT-stained ICS1 treated with water, (B) NBT-stained Sca6 treated with water, (C) NBT-stained ICS1 treated with SA, (D) NBT-stained Sca6 treated with SA, (E) DAB-stained ICS1 treated with water, (F) DAB-stained Sca6 treated with water, (G) DAB-stained ICS1 treated with SA, (H) DAB-stained Sca6 treated with SA. Scale bars, 1cm. (I) Mean product area of leaf disc stained by NBT and mean grey value of stained area for each genotype and treatment. (J) Mean product of area stained by DAB and mean grey value of stained area for each genotype and treatment. In I and J, differences between bars marked with the same letter are not statistically significant (Fisher’s PLSD analysis *P*>0.05), standard errors were calculated from five biological replicates, and each biological replicate is a plate containing three leaf discs.

Some chloroplast and mitochondrial proteins, such as photosystem components and NADH dehydrogenase, function in electron transfer, contributing to the production of ROS (Moller, 2001; Gechev *et al.*, 2006; Noctor *et al.*, 2007; Sharma *et al.*, 2012). As chloroplast and mitochondrial genes were significantly induced in SA-treated Sca6 and several class III peroxidases were induced in SA-treated ICS1, we hypothesized that the production of ROS in the two genotypes could be driven by different mechanisms. This may contribute to Sca6’s greater resistance to certain pathogens and perhaps the faster rate of senescence in ICS1. To test whether accumulation of ROS differed between the two genotypes, we performed nitroblue tetrazolium (NBT) and 3,3′-diaminobenzidine (DAB) staining to quantify the accumulation of superoxide and peroxide, respectively (Fig. 7). NBT reacts with superoxide to form a blue precipitate and DAB reacts with hydrogen peroxide, forming a brown precipitate. Plants of both genotypes were spray treated with 2mM SA or water, then 24h after treatment, stage C leaves were harvested from the trees and leaf discs were stained with DAB or NBT (Fig. 7A−H). ROS staining was quantified in replicated samples as described in ‘Materials and methods’. Superoxide accumulation was significantly higher in SA-treated Sca6 than in any other genotype-treatment pair (Fisher’s PLSD *P*=0.019 for Sca6 H2O vs. Sca6 SA) (Fig. 7I). Hydrogen peroxide accumulation increased in both genotypes with SA treatment (Fig. 7J) (Fisher’s PLSD *P*=0.002 for ICS1 H2O versus ICS1 SA and *P*=0.001 for Sca6 H2O versus Sca6 SA). A significant difference in peroxide accumulation was also detected between water-treated ICS1 and Sca6 (Fisher’s PLSD *P*=0.002). This difference in ROS accumulation in water-treated leaf tissue could be an indication of a true difference in basal ROS levels in the leaves or it could be attributed to a faster or stronger wound response in ICS1 that is associated with an ROS burst after excising the leaf discs.

## Discussion

While the role of SA in model plants has been well studied (Vlot *et al.*, 2009; Fu and Dong, 2013), we sought to verify its role in cacao defence and to explore the molecular basis of the dramatic differences in resistance to pathogens between two cacao genotypes. We focused our analysis on the SA response, as it is the phytohormone thought to be most important for regulation of defence against biotrophs and hemibiotrophs (Fu and Dong, 2013) and on the difference in response between Sca6 and ICS 1, two genotypes strongly contrasting in their resistance phenotypes (Risterucci *et al.*, 2003; Faleiro *et al.*, 2006). Our data showed that treatment of leaves with 1mM SA reduced lesion size and pathogen biomass in both ICS1 (more susceptible) and Sca6 (more tolerant) after inoculation of detached leaves with *P. tropicalis,* and their relative susceptibilities were consistent with the known disease resistance phenotypes of these two varieties. We also demonstrated that the cacao homologue to Arabidopsis NPR1 positively contributes to defence in cacao, reinforcing the importance of the SA response in cacao and supporting our previous results. (Shi *et al.*, 2010). Our microarray analysis revealed statistically significant differential transcript abundance for ∼1000 genes in each genotype in response to SA, close to the number of genes induced by SA in Arabidopsis (Wang *et al.*, 2006). Interestingly, our study also suggested that there is a significant genotype specificity in the cacao defence response pathway.

A hallmark of induction of systemic acquired resistance is the increased expression of PR genes. Our data revealed that in response to SA treatment, more PR gene transcripts levels were elevated in the susceptible genotype ICS1 than in the tolerant genotype Sca6. This suggested that surprisingly, PR transcript levels were not correlated with the higher resistance of Sca6. Our results are in agreement with to those of Teixeira *et al.* (2014), who showed that transcriptional changes in the susceptible cacao cultivar ‘Comum’ in response to WBD infection revealed increased transcript levels of PR genes. The measurements of lesion growth and pathogen biomass with and without SA treatment combined with our microarray data suggest that SA-induced gene expression does partially contribute to resistance in ICS1, but it is unclear if the PR genes contribute directly to the observed resistance.

A second plausible hypothesis to explain these observations is that perhaps the higher basal transcript levels of defence genes mediates the resistance of Sca6 that is consequently enhanced by the SA treatment. However, our analysis of basal gene transcript levels indicated that gene transcript levels within several defence-related terms, including ‘Immune Response’ (GO:0006955), ‘Defence Response’ (GO:0006952), ‘Response to Fungus’ (GO:0009620), and ‘Response to Chitin’ (GO:0010200), were higher in basal ICS1 (Supplementary Table S8). Detection of PR transcripts in the water-treated samples from the genotypes by the microarray and qRT-PCR also provide evidence against this hypothesis.

Interestingly, in our study nearly half of the PR genes induced in ICS1 by SA were class III peroxidase family members, proteins that are secreted into the cell wall or the apoplast and contribute to ROS generation (O’Brien *et al.*, 2012; Baxter *et al.*, 2013). Moreover, the SA treatment of Sca6 leaves induced the transcript levels of genes located in the chloroplast and mitochondrial genomes also known to contribute to ROS production; this increase could have resulted either from an increase in the transcription rate of the genes, or an increase in the number of organelles or content of organellar DNA. Among this set of genes were mitochondrial NADPH dehydrogenases and chloroplastic photosystem I and II components, both known to be involved in the oxidative burst, a major plant defence mechanism (Rao *et al.*, 1997; Torres and Dangl, 2005; Pogany *et al.*, 2009; Dubreuil-Maurizi *et al.*, 2010). Thus we hypothesized that the mechanism of resistance to pathogens in Sca6 involves expression of genes involved in ROS accumulation in the chloroplasts and mitochondria.

The results from GO enrichment analysis generally supported the hypothesis that ROS production strategy differed between the genotypes. While PAGE analysis (Kim and Volsky, 2005) revealed that both genotypes had an general up-regulation of genes annotated with the ‘Response to Oxidative Stress’ term, a major difference between the genotypes was elevation of transcripts of genes annotated with plastid-related GO terms in Sca6 and reduced transcripts of the same classes of genes in ICS1. Additionally, comparing the induced (SA-treated) states of the two genotypes, transcripts of genes annotated with ‘Response to Fungus’ remained higher in ICS1, but gene transcripts annotated with a variety of chloroplast-related terms were again more highly elevated in Sca6. Our data provides strong evidence for the importance of ROS generated in chloroplasts and mitochondria in the Sca6 genotype.

Twenty-four hours after SA treatment, more superoxide accumulated in Sca6, while there was no difference in hydrogen peroxide accumulation between the genotypes. This supports the interpretation of the transcriptome analysis; that higher ROS accumulation in the chloroplast may be unique to Sca6. While SA-treated samples from the genotypes did not differ in hydrogen peroxide accumulation, water-treated Sca6 had less hydrogen peroxide than water-treated ICS1. Thus there was a greater burst of both ROS types resulting from SA treatment in Sca6. The role of ROS in plant defence has been an active area of research (Apel and Hirt, 2004). Production of superoxide has previously been linked to development of hypersensitive response in potato tubers inoculated with *P. infestans* (Doke, 1983), which is consistent with our finding that the resistant cacao genotype had greater superoxide accumulation in response to another *Phytophthora* species, *P. tropicalis*. Further, it is possible that differences in localization of ROS production differentially affect signalling pathways mediating resistance. It has been demonstrated that the elevation of endogenous SA can induce the production of ROS, which will in turn facilitate cell death at the site of infection (Apel and Hirt, 2004; Torres *et al.*, 2006; Mur *et al.*, 2008). As generation of ROS within the chloroplast (Liu *et al.*, 2007) and mitochondria (Cvetkovska and Vanlerberghe, 2012) have been linked to the hypersensitive response, further investigation of these processes in cacao and the differences between induction of these pathways in susceptible and resistant genotypes is needed. Alternatively, ROS production in response to WBD infection has been proposed to accelerate necrosis, as elevated peroxide concentration could lead to cell death, greater nutrient availability, and a more rapid progression to the pathogen’s necrotrophic stage (de Oliveira Ceita *et al.*, 2007). As we detected higher peroxide concentrations in water-treated ICS1 leaves than in water-treated Sca6, it is possible that higher basal ROS levels in the susceptible genotype accelerate its infection by *Phytophthora* as has been proposed with WBD.

Our results reveal several important defence-related physiological differences between the two cacao genotypes. Further research is needed to explore more comprehensively the role of PR gene expression and ROS production in the immune response of cacao. Ultimately, this knowledge can be used to benefit cacao farmers and breeders by providing molecular strategies and markers for accelerated and efficient plant breeding.

## Supplementary data

Supplementary data are available at *JXB* online.

Supplementary Fig. S1. Vector map for the binary plasmid used for transient overexpression of TcNPR1.

Supplementary Table S1. All genes in the Sca6 genotype with differential regulation from SA treatment.

Supplementary Table S2. All genes in the ICS1 genotype with differential regulation from SA treatment.

Supplementary Table S3. Genes showing statistically significant differential regulation in both Sca6 and ICS1 genotypes.

Supplementary Table S4. Differential regulation of PR genes according to genotype, as detected by the microarray.

Supplementary Table S5. Enriched GO terms as detected by PAGE for Sca6.

Supplementary Table S6. Enriched GO terms as detected by PAGE for ICS1.

Supplementary Table S7. Enriched GO terms for SA effect when averaging all samples, across genotypes.

Supplementary Table S8. GO term enrichment by PAGE comparing basal (water-treated) and induced (SA-treated) states of Sca6.

Supplementary Table S9. GO term enrichment by PAGE comparing basal (water-treated) and induced (SA-treated) states of ICS1.

Supplementary Table S10. Primers used for qRT-PCR.

Supplementary Data
