# Supplementary material for: Two Theobroma cacao genotypes with contrasting pathogen tolerance show aberrant transcriptional and ROS responses after salicylic acid treatment
Source: J Exp Bot. 2015 Jul 10;66(20):6245–58. doi: 10.1093/jxb/erv334 (PMC4588882; doi:10.1093/jxb/erv334)
Supplement: Supplementary Data [file supp_66_20_6245__index.html]

Two Theobroma cacao genotypes with contrasting pathogen tolerance show aberrant transcriptional and ROS responses after salicylic acid treatment — Two Theobroma cacao genotypes with contrasting pathogen tolerance show aberrant transcriptional and ROS responses after salicylic acid treatment — Supplementary Data 

# Two *Theobroma cacao* genotypes with contrasting pathogen tolerance show aberrant transcriptional and ROS responses after salicylic acid treatment

## Supplementary Data

Data files

- Supplementary Data - Supplementary Data
