# Supplementary material for: Two Theobroma cacao genotypes with contrasting pathogen tolerance show aberrant transcriptional and ROS responses after salicylic acid treatment
Source: J Exp Bot. 2015 Jul 10;66(20):6245–58. doi: 10.1093/jxb/erv334 (PMC4588882; doi:10.1093/jxb/erv334)
Supplement: Supplementary Data [file supp_erv334_jexbot149799_file001.pdf]

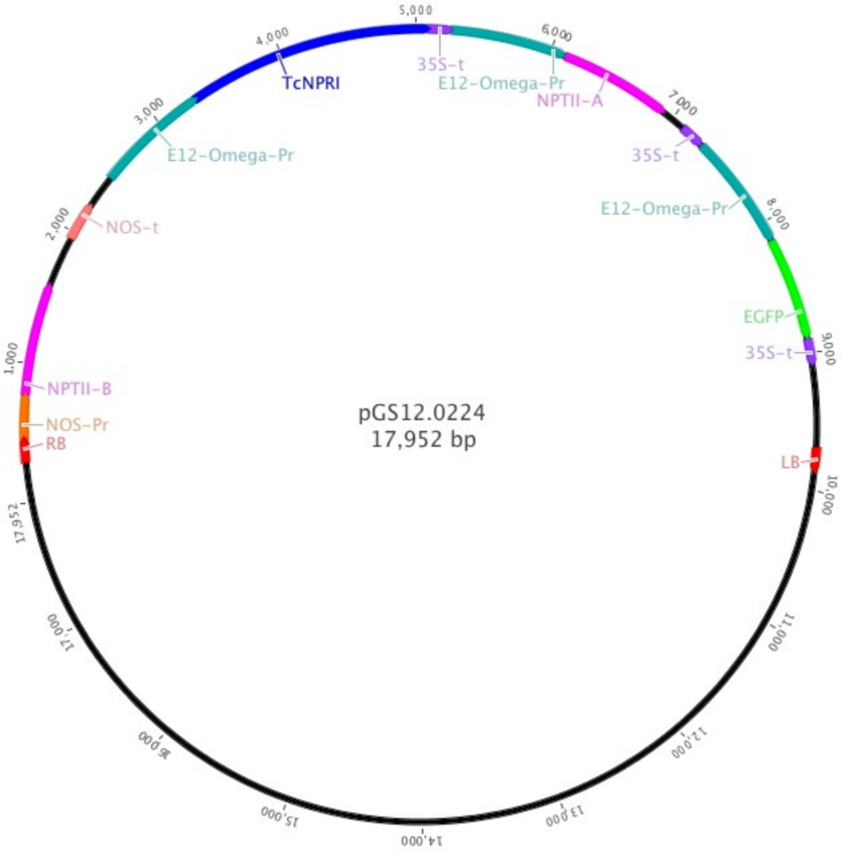

Supplemental Figure 1 - Illustration of binary plasmid pGS12.0224 containing the *TcNPR1* transgene (GenBank: KP844565). Components of this vector include: E12-Ω-CaMV-35S promoter (E12-Omega-Pr) (Mitsuhara et al. 1996), the CaMV-35S terminator sequence (35S-t), the cacao cDNA sequence encoding *TcNPR1,* cloned from genotype Scavina 6, the *NPTII* kanamycin selectable marker gene (*NPTII-A*)(De-Block et al. 1984), the NOS promoter (Nos-Pr), the NOS terminator (NOS-t) (Lichtenstein and Fuller, 1987), and the EGFP gene encoding green fluorescent protein (Clontech). RB and LB indicate the right and left borders of the Ti plasmid T-DNA region, respectively. Position in base pairs is annotated outside of the vector.

Supplemental Table 1 - Genes with statistically significant differential regulation in genotype Sca6

TC ID	Log_2 Fold Change ($SA-H_2O$)[a]	Actual Fold Change ($SA-H_2O$)[a]	p-value[a]	BH-adjusted p-value[a]	Arabidopsis Annotation	AThal BestHitID
TC04G003810	2.73	6.63	0.00	0.000	Photosystem_II_CP47_chlorophyll_apoprotein	ATCG00680
TC09G005040	2.54	5.81	0.00	0.006	NA	NA
TC09G030710	2.29	4.90	0.00	0.033	18.2_kDa_class_I_heat_shock_protein	NA
TC05G000850	1.97	3.93	0.00	0.003	Hypothetical_protein	ATMG00285
TC04G003660	1.94	3.84	0.00	0.000	Maturase_K	ATCG00020
TC04G003910	1.93	3.81	0.00	0.000	Photosystem_I_P700_chlorophyll_a_apoprotein_A1	ATCG00340
TC05G000830	1.75	3.36	0.00	0.004	Hypothetical_protein	ATMG01320
TC05G000660	1.73	3.32	0.00	0.005	60S_ribosomal_protein_L2,_mitochondrial	ATMG00560
TC04G003680	1.63	3.10	0.00	0.011	Os04g0234750_protein_(Fragment)	ATCG01240
TC09G030700	1.61	3.05	0.00	0.018	17.3_kDa_class_I_heat_shock_protein	AT1G53540
TC08G010170	1.59	3.01	0.00	0.017	NA	NA
TC06G002600	1.57	2.97	0.00	0.011	Putative_Cytoskeleton-associated_protein_5	AT2G35630
TC00G010710	1.52	2.87	0.00	0.000	NAD(P)H-quinone_oxidoreductase_subunit_1,_chloroplastic	ATCG01090
TC02G003030	1.50	2.83	0.00	0.007	Putative_uncharacterized_protein	NA
TC00G058430	1.48	2.79	0.00	0.000	Putative uncharacterized protein	NA
TC00G053610	1.44	2.72	0.00	0.000	Hypothetical_protein	NA
TC00G007720	1.44	2.71	0.00	0.001	NADH-ubiquinone_oxidoreductase_chain_4L	ATMG00650
TC04G003900	1.42	2.68	0.00	0.007	Photosystem_II_CP43_chlorophyll_apoprotein	ATCG00280
TC05G000740	1.40	2.65	0.00	0.001	NADH-ubiquinone_oxidoreductase_chain_1	ATMG01275
TC06G015300	1.38	2.61	0.00	0.006	Homeobox_protein,_putative	AT1G28420
TC06G001520	1.38	2.60	0.00	0.018	NA	NA
TC07G017570	1.36	2.57	0.00	0.004	NA	NA
TC04G003850	1.35	2.55	0.00	0.000	Ribulose_bisphosphate_carboxylase_large_chain	ATCG00490
TC08G015000	1.34	2.53	0.00	0.015	Synaptotagmin,_putative	AT1G22610
TC05G001020	1.33	2.52	0.00	0.000	NADH-ubiquinone_oxidoreductase_chain_4	ATMG00580
TC05G001190	1.32	2.50	0.00	0.002	Hypothetical_protein	AT2G34520
TC03G024630	1.31	2.49	0.00	0.001	E3_ubiquitin-protein_ligase_HOS1	AT2G39810
TC05G000960	1.28	2.44	0.00	0.000	Cytochrome_c_oxidase_subunit_2	ATMG00160
TC09G031960	1.28	2.42	0.00	0.000	Heat_shock_cognate_70_kDa_protein	AT3G12580
TC09G013930	1.27	2.41	0.00	0.002	Putative_Phospholipase_A-2-activating_protein	AT3G18860
TC03G018090	1.26	2.39	0.00	0.034	23.6_kDa_heat_shock_protein,_mitochondrial	AT4G25200

TC03G025570	1.25	2.37	0.00	0.001	Heat_shock_protein_STI	AT1G62740
TC05G000940	1.25	2.37	0.00	0.000	Ribosomal_protein_S10,_mitochondrial	ATMG00640
TC00G068130	1.25	2.37	0.00	0.005	Hypothetical_protein	ATCG00180
TC05G000640	1.24	2.36	0.00	0.029	Ribosomal_protein_S7,_mitochondrial	ATMG01270
TC01G031600	1.23	2.35	0.00	0.000	NA	NA
TC00G084770	1.22	2.33	0.00	0.004	Putative_uncharacterized_protein	AT5G16780
TC09G006300	1.19	2.29	0.00	0.036	Phosphoinositide_5-phosphatase,_putative	NA
TC05G001380	1.18	2.27	0.00	0.014	Cytochrome_c_oxidase_subunit_1	AT1G77750
TC04G003970	1.18	2.26	0.00	0.000	Cytochrome_b6	ATCG00710
TC04G003690	1.17	2.26	0.00	0.008	Putative_uncharacterized_protein	NA
TC04G003740	1.16	2.23	0.00	0.000	Unknow_protein	NA
TC04G003840	1.15	2.23	0.00	0.000	Apocytochrome_f	ATCG00540
TC04G022750	1.14	2.21	0.00	0.010	NA	NA
TC00G028390	1.13	2.19	0.00	0.002	Cohesin_subunit_rad21,_putative	NA
TC00G057900	1.12	2.18	0.00	0.001	Transportin-3,_putative	AT1G12930
TC06G015310	1.11	2.16	0.00	0.012	Putative_DnaJ_homolog_subfamily_B_member_4	AT2G20560
TC09G018700	1.09	2.13	0.00	0.005	Pumilio,_putative	AT3G20250
TC00G089300	1.09	2.12	0.00	0.005	Heat_shock_protein_101	AT1G74310
TC05G003590	1.09	2.12	0.00	0.038	Putative_uncharacterized_protein	AT5G02020
TC04G023080	1.08	2.12	0.00	0.005	NEDD8-activating_enzyme_E1_catalytic_subunit	AT5G19180
TC04G003830	1.08	2.11	0.00	0.018	Cytochrome_b559_subunit_alpha	ATCG00550
TC04G003720	1.08	2.11	0.00	0.005	NAD(P)H-quinone_oxidoreductase_subunit_5,_chloroplastic_(Fragment)	ATCG01010
TC09G014710	1.05	2.07	0.00	0.029	NA	NA
TC05G000880	1.04	2.06	0.00	0.002	Hypothetical_protein	NA
TC01G033560	1.01	2.02	0.00	0.028	Putative_Protein_LHY	AT1G01060
TC02G009700	1.01	2.01	0.00	0.014	Putative_uncharacterized_protein	AT2G15020
TC00G080910	1.00	2.01	0.00	0.006	Bifunctional_protein_folD	NA
TC09G016580	1.00	2.00	0.00	0.010	STS14_protein,_putative	AT4G25780
TC09G012660	0.98	1.98	0.00	0.016	NA	NA
TC00G035130	0.98	1.97	0.00	0.031	Putative_Two-component_response_regulator-like_APRR2	AT5G44190
TC03G022890	0.98	1.97	0.00	0.008	Putative_Methionine_gamma-lyase	AT1G64660
TC03G016040	0.97	1.96	0.00	0.000	Predicted_protein	AT5G61820
TC04G003920	0.97	1.96	0.01	0.040	30S_ribosomal_protein_S4,_chloroplastic	ATCG00380
TC04G003730	0.95	1.94	0.01	0.039	Uncharacterized_protein_ycf68	NA
TC09G004830	0.95	1.94	0.00	0.000	Glycine_dehydrogenase_[decarboxylating],_mitochondrial	AT4G33010
TC01G021030	0.95	1.93	0.00	0.004	70_kDa_peptidyl-prolyl_isomerase	AT3G25230
TC03G021040	0.94	1.92	0.00	0.004	Predicted_protein	AT5G53020

TC09G019330	0.94	1.92	0.00	0.000	Putative_uncharacterized_protein	AT5G24710
TC05G023590	0.94	1.92	0.00	0.006	Putative_Speckle-type_POZ_protein	AT3G03740
TC00G032610	0.92	1.90	0.00	0.035	Putative_Subtilisin-like_protease	AT5G59810
TC03G012370	0.91	1.88	0.00	0.024	Hypothetical_protein	AT4G26190
TC06G011310	0.90	1.87	0.00	0.000	Putative_Transforming_growth_factor-beta_receptor-associated_protein_1_homolog	AT1G22860
TC01G003880	0.90	1.87	0.00	0.002	BEL1-like_homeodomain_protein_2	NA
TC02G030610	0.90	1.86	0.00	0.008	Glutamate_receptor_3.7	NA
TC04G002800	0.88	1.84	0.00	0.038	NA	NA
TC04G003880	0.88	1.84	0.00	0.010	DNA-directed_RNA_polymerase_subunit_beta	ATCG00180
TC04G015080	0.88	1.84	0.00	0.004	NA	NA
TC01G033460	0.88	1.83	0.00	0.008	Hypothetical_protein	AT1G33060
TC02G029930	0.88	1.83	0.00	0.000	Predicted_protein	AT1G67960
TC05G000780	0.87	1.83	0.00	0.000	Hypothetical_protein	ATMG00030
TC05G001060	0.86	1.82	0.00	0.036	Hypothetical_protein	NA
TC10G003040	0.86	1.81	0.00	0.000	Putative_Probable_E3_ubiquitin-protein_ligase_HERC2	AT3G55580
TC09G006720	0.86	1.81	0.00	0.010	Inter-alpha-trypsin_inhibitor_heavy_chain,_putative	AT1G19110
TC01G026000	0.86	1.81	0.00	0.001	Putative_Pre-mRNA-processing_factor_39	AT1G04080
TC03G027270	0.84	1.79	0.00	0.011	Putative_peroxisomal-coenzyme_A_synthetase	AT3G48990
TC02G006670	0.84	1.79	0.00	0.035	NA	NA
TC05G001010	0.83	1.78	0.00	0.002	Hypothetical_protein	ATMG00580
TC04G010170	0.83	1.78	0.00	0.011	Alpha-1,4_glucan_phosphorylase_L_isozyme,_chloroplastic/amyloplastic	AT3G29320
TC02G031640	0.83	1.78	0.00	0.024	Putative_Nucleolar_protein_14	AT1G69070
TC07G007410	0.83	1.77	0.00	0.000	Transcription_factor,_putative	AT5G54470
TC08G006100	0.82	1.77	0.00	0.003	Putative_uncharacterized_protein	AT1G58230
TC04G026780	0.81	1.76	0.00	0.018	Beta-amylase_1,_chloroplastic	AT3G23920
TC00G061020	0.81	1.76	0.00	0.003	Putative_uncharacterized_protein	NA
TC00G080340	0.81	1.75	0.00	0.036	Putative_Predicted_protein	AT4G15248
TC10G016440	0.80	1.75	0.00	0.023	NA	NA
TC05G031900	0.80	1.74	0.00	0.015	Phytochrome_A	AT1G09570
TC08G009450	0.80	1.74	0.00	0.030	Putative_Acetyl-CoA_carboxylase	AT1G36160
TC06G012620	0.80	1.74	0.00	0.029	Putative_Calcyclin-binding_protein	AT1G30070
TC00G057520	0.79	1.73	0.00	0.000	Putative_uncharacterized_protein	AT3G54500
TC03G029250	0.79	1.73	0.00	0.007	Electron_transporter,_putative	AT1G64500
TC02G023370	0.79	1.73	0.00	0.007	Putative_uncharacterized_protein	AT1G60200
TC09G006990	0.78	1.72	0.00	0.022	NA	NA
TC02G012180	0.78	1.72	0.00	0.002	Probable_WRKY_transcription_factor_19	AT5G64550
TC07G004100	0.78	1.72	0.00	0.000	Predicted_protein	AT4G27030

TC01G033080	0.77	1.71	0.01	0.047	Predicted_protein	AT3G62070
TC03G015700	0.77	1.71	0.00	0.003	ATP-dependent_Clp_protease_ATP-binding_subunit_clpC_homolog,_chloroplastic	AT5G50920
TC09G013650	0.77	1.70	0.00	0.002	Bromodomain-containing_protein,_putative	AT5G65630
TC05G019340	0.77	1.70	0.00	0.017	Aspartic_proteinase-like_protein_2	NA
TC09G033330	0.76	1.69	0.00	0.001	4-hydroxy-3-methylbut-2-en-1-yl_diphosphate_synthase	AT5G60600
TC03G013700	0.76	1.69	0.01	0.046	Cytochrome_P450_71A25	AT3G48280
TC08G012200	0.76	1.69	0.00	0.013	Putative_Pre-rRNA-processing_protein_esf1	AT3G01160
TC01G034610	0.76	1.69	0.01	0.049	Abscisic_acid_receptor_PYL9	AT1G01360
TC09G032580	0.75	1.69	0.01	0.049	18.5_kDa_class_I_heat_shock_protein	AT2G29500
TC00G006890	0.75	1.69	0.00	0.004	Putative_uncharacterized_protein	AT5G05140
TC09G001070	0.75	1.69	0.00	0.028	Zinc_finger_protein_CONSTANS-LIKE_4	AT5G24930
TC09G018460	0.75	1.68	0.00	0.005	Predicted_protein	AT5G56360
TC05G003840	0.75	1.68	0.00	0.000	Delta-1-pyrroline-5-carboxylate_synthase	AT2G39800
TC06G003440	0.74	1.67	0.00	0.015	Putative_F-box/LRR-repeat_protein_14	AT1G15740
TC03G006290	0.73	1.66	0.00	0.000	Predicted_protein	AT5G17300
TC00G012490	0.73	1.66	0.00	0.001	Digalactosyldiacylglycerol_synthase_1,_chloroplastic	AT3G11670
TC05G006130	0.73	1.66	0.01	0.045	Putative_uncharacterized_protein	AT2G42760
TC01G018530	0.73	1.66	0.00	0.005	Putative_Heat_shock_protein_90	AT3G07770
TC03G030500	0.72	1.65	0.00	0.000	Putative_uncharacterized_protein	AT1G72030
TC08G011870	0.72	1.65	0.00	0.008	2-oxoisovalerate_dehydrogenase_subunit_alpha,_mitochondrial	AT1G21400
TC06G019780	0.72	1.65	0.00	0.014	Protein_STRUBBELIG-RECEPTOR_FAMILY_8	NA
TC01G019270	0.71	1.64	0.00	0.000	Putative_uncharacterized_protein	AT2G04240
TC05G001160	0.71	1.64	0.00	0.038	NA	NA
TC04G024360	0.71	1.64	0.00	0.003	Early_light-induced_protein,_chloroplastic	AT4G14690
TC10G002920	0.70	1.63	0.00	0.020	Ribulose_bisphosphate_carboxylase/oxygenase_activase_1,_chloroplastic	AT2G39730
TC05G020320	0.70	1.62	0.00	0.008	Serine/arginine_rich_splicing_factor,_putative	AT3G13570
TC04G030230	0.70	1.62	0.00	0.036	NA	NA
TC05G007180	0.70	1.62	0.00	0.009	NA	NA
TC09G000610	0.69	1.61	0.00	0.033	Plasma_membrane_ATPase_4	AT2G24520
TC04G003790	0.68	1.61	0.00	0.002	30S_ribosomal_protein_S8,_chloroplastic	ATCG00740
TC07G000690	0.68	1.60	0.00	0.011	Putative_uncharacterized_protein	AT3G13225
TC01G020510	0.68	1.60	0.00	0.000	Uncharacterized_UDP-glucosyltransferase_At1g05670	AT1G05680
TC01G025400	0.68	1.60	0.00	0.020	DNA_repair_and_recombination_protein_RAD26,_putative	AT2G18760
TC04G016790	0.67	1.60	0.00	0.003	Protein_transporter,_putative	AT2G38410
TC06G014190	0.67	1.59	0.01	0.040	CBL-interacting_protein_kinase_5	AT5G45820
TC01G028390	0.67	1.59	0.01	0.042	NA	NA

TC01G014610	0.67	1.59	0.00	0.038	AML1	AT1G29400
TC00G017110	0.67	1.59	0.00	0.006	Hypothetical_protein	AT4G03420
TC08G010800	0.67	1.59	0.00	0.000	Putative_Protein_tolB	AT1G21680
TC01G022340	0.67	1.59	0.00	0.000	Putative_PPPDE_peptidase_domain-containing_protein_2	AT3G07090
TC04G020820	0.66	1.58	0.00	0.003	Predicted_protein	AT4G00440
TC03G015720	0.66	1.58	0.00	0.000	Auxin_response_factor_2	AT5G62000
TC04G021580	0.66	1.58	0.00	0.021	Protein_ABC1,_mitochondrial,_putative	AT3G24190
TC09G023620	0.66	1.58	0.00	0.000	Serine--glyoxylate_aminotransferase	AT2G13360
TC01G001040	0.65	1.57	0.00	0.011	Phospholipase_D_delta	AT4G35790
TC04G001640	0.65	1.57	0.00	0.001	Hypothetical_protein	AT5G40450
TC01G017530	0.65	1.57	0.00	0.001	NA	NA
TC04G015630	0.65	1.57	0.00	0.014	NA	NA
TC05G014280	0.65	1.57	0.00	0.002	NA	NA
TC05G015130	0.65	1.56	0.01	0.047	N-acylneuraminate-9-phosphatase,_putative	AT2G41250
TC03G019930	0.64	1.56	0.00	0.002	Beta-carotene_hydroxylase	AT5G52570
TC02G011760	0.64	1.56	0.00	0.000	Putative_uncharacterized_protein	AT5G64170
TC03G015550	0.64	1.56	0.00	0.000	Putative_uncharacterized_protein	AT3G26890
TC09G027160	0.64	1.56	0.00	0.005	Ubiquitin_carboxyl-terminal_hydrolase_12	AT3G11910
TC09G035070	0.64	1.55	0.00	0.003	Probable_serine/threonine-protein_kinase_WNK4	AT3G51630
TC04G003870	0.63	1.55	0.00	0.027	Photosystem_I_assembly_protein_ycf3	ATCG00360
TC09G034390	0.63	1.55	0.00	0.035	Ubiquitin-activating_enzyme_E1_2	AT2G30110
TC09G030000	0.63	1.54	0.01	0.044	Predicted_protein_(Fragment)	AT5G58770
TC06G012200	0.62	1.54	0.00	0.010	CBL-interacting_serine/threonine-protein_kinase_23	AT1G30270
TC06G014280	0.62	1.54	0.00	0.013	Putative_Cysteine_proteinase_RD21a	AT1G09850
TC02G031310	0.62	1.54	0.00	0.000	Inositol_or_phosphatidylinositol_kinase,_putative	AT1G26270
TC03G029940	0.62	1.53	0.00	0.002	Predicted_protein	AT1G32230
TC05G005790	0.62	1.53	0.00	0.024	Putative_Myb-like_protein_G	AT3G09600
TC01G017060	0.62	1.53	0.00	0.000	Putative_Predicted_protein_(Fragment)	AT1G05320
TC03G029370	0.61	1.53	0.00	0.000	Transcription_factor,_putative	AT1G64530
TC00G036580	0.61	1.53	0.00	0.014	Prolyl_4-hydroxylase_alpha_subunit,_putative	AT3G28480
TC04G003820	0.61	1.53	0.00	0.000	ATP-dependent_Clp_protease_proteolytic_subunit	ATCG00660
TC09G005690	0.61	1.52	0.00	0.010	Putative_copper-transporting_ATPase_PAA1	AT5G21930
TC03G021560	0.61	1.52	0.00	0.002	Putative_uncharacterized_protein	AT5G53620
TC10G007380	0.61	1.52	0.00	0.001	Putative_RING_finger_protein_B	NA
TC00G054350	0.61	1.52	0.00	0.011	Putative_Protein_transport_protein_SEC23	AT2G27460
TC09G034240	0.61	1.52	0.00	0.006	Putative_uncharacterized_protein	AT5G26760
TC03G022970	0.61	1.52	0.00	0.021	Metal-nicotianamine_transporter_YSL1	AT5G53550

TC08G002840	0.60	1.51	0.00	0.003	NA	NA
TC02G015800	0.60	1.51	0.00	0.013	Putative_Pre-mRNA-processing_factor_6	AT4G03430
TC09G003520	0.59	1.51	0.00	0.005	Predicted_protein	AT5G11490
TC00G092080	0.59	1.51	0.01	0.042	Putative_uncharacterized_protein	NA
TC01G033650	0.59	1.50	0.00	0.000	Putative_Two-component_response_regulator-like_PRR95	AT5G24470
TC05G005640	0.58	1.50	0.00	0.001	Putative_SAP_domain-containing_ribonucleoprotein	AT5G02770
TC07G004210	0.58	1.50	0.00	0.036	DNA_polymerase_I,_putative	AT1G50840
TC03G017540	0.58	1.49	0.00	0.000	RNA_polymerase_sigma_factor_rpoD,_putative	AT5G24120
TC02G034270	0.58	1.49	0.01	0.043	Prp4,_putative	AT3G16460
TC03G002220	0.58	1.49	0.00	0.001	Zinc_ion_binding_protein,_putative	AT4G24690
TC01G029220	0.57	1.49	0.00	0.000	Probable_gibberellin_receptor_GID1L2	AT3G63010
TC03G016980	0.57	1.49	0.00	0.000	NA	NA
TC02G006330	0.57	1.49	0.00	0.008	Arginine_decarboxylase	AT2G16500
TC03G017610	0.57	1.49	0.00	0.011	Putative_Solute_carrier_family_25_member_39	AT4G27940
TC06G004130	0.57	1.48	0.00	0.030	Putative_uncharacterized_protein	AT1G15910
TC09G002820	0.57	1.48	0.00	0.001	Putative_Ubiquitin_carboxyl-terminal_hydrolase	AT5G57990
TC04G012450	0.56	1.48	0.00	0.013	Zinc_finger_protein_CONSTANS-LIKE_2	AT3G02380
TC00G035340	0.56	1.47	0.00	0.002	F-box/ankyrin_repeat_protein_SKIP35	AT3G59910
TC08G004140	0.56	1.47	0.00	0.028	Putative_Nucleoredoxin	AT1G60420
TC01G012570	0.56	1.47	0.00	0.004	Putative_Probable_E3_ubiquitin-protein_ligase_HERC4	AT5G63860
TC06G019980	0.56	1.47	0.00	0.006	E3_ubiquitin-protein_ligase_COP1	AT2G32950
TC02G033820	0.55	1.47	0.00	0.002	Putative_UDP-galactose_transporter_homolog_1	AT1G14360
TC09G024910	0.55	1.46	0.00	0.001	Transcription_factor,_putative	AT2G26770
TC09G026080	0.55	1.46	0.00	0.003	Aluminum-activated_citrate_transporter	AT1G51340
TC03G021650	0.55	1.46	0.00	0.003	RNA-binding_protein,_putative	AT3G49390
TC09G033190	0.54	1.46	0.00	0.038	NA	NA
TC00G035620	0.54	1.45	0.00	0.036	Hypothetical_protein	NA
TC00G001500	0.54	1.45	0.01	0.042	NBS_resistance_protein	NA
TC00G030570	0.53	1.45	0.00	0.021	Probable_UDP-N-acetylglucosamine--peptide_N-acetylglucosaminyltransferase_SPINDLY	NA
TC04G000980	0.53	1.45	0.00	0.017	Carbonic_anhydrase,_chloroplastic	AT5G14740
TC09G019460	0.53	1.45	0.00	0.001	Putative_mRNA-capping_enzyme	AT3G09100
TC00G089850	0.53	1.45	0.01	0.048	Putative_uncharacterized_protein	AT4G29950
TC02G002350	0.53	1.44	0.00	0.018	Pinin,_putative	AT4G33740
TC08G011380	0.53	1.44	0.01	0.049	Photosystem_II_22_kDa_protein,_chloroplastic	AT1G44575
TC04G024350	0.53	1.44	0.00	0.014	Putative_Dymeclin	AT1G04200
TC04G024900	0.52	1.44	0.00	0.036	Predicted_protein	AT1G04300
TC05G006380	0.52	1.44	0.00	0.000	Aquaporin_PIP2-2	AT2G37170

TC02G006400	0.52	1.44	0.00	0.000	Probable_fructose-bisphosphate_aldolase_1,_chloroplastic	AT4G38970
TC01G037190	0.52	1.44	0.00	0.005	ATP_binding_protein,_putative	AT2G45590
TC07G013070	0.52	1.44	0.00	0.003	Molybdenum_cofactor_sulfurase	AT1G16540
TC04G010580	0.52	1.44	0.00	0.008	Protein_RIK	AT3G29390
TC02G024970	0.52	1.43	0.00	0.026	Protein_binding_protein,_putative	AT1G24580
TC01G030930	0.52	1.43	0.00	0.003	Predicted_protein	AT4G02260
TC09G026580	0.51	1.43	0.00	0.037	At3g45600	AT3G45600
TC08G013850	0.51	1.43	0.00	0.002	Putative_Far_upstream_element-binding_protein_3	AT1G33680
TC01G001330	0.51	1.42	0.00	0.003	Putative_Vacuolar_protein_sorting-associated_protein_35	AT2G17790
TC05G002580	0.51	1.42	0.00	0.017	Predicted_protein	AT5G01520
TC06G011960	0.51	1.42	0.00	0.036	Putative_uncharacterized_protein	NA
TC05G020250	0.51	1.42	0.00	0.001	Eukaryotic_translation_initiation_factor_3_subunit_C	AT3G56150
TC07G000190	0.50	1.42	0.00	0.013	Probable_WRKY_transcription_factor_2	NA
TC00G061630	0.50	1.42	0.00	0.006	Putative_DNA-binding_protein_SMUBP-2	AT2G03270
TC02G003500	0.50	1.41	0.00	0.000	Putative_uncharacterized_protein	AT4G35270
TC04G016190	0.50	1.41	0.00	0.006	Vacuolar-sorting_receptor_1	AT2G30290
TC01G037820	0.49	1.41	0.00	0.008	Transketolase,_chloroplastic	AT2G45290
TC09G001560	0.49	1.41	0.00	0.012	Nucleic_acid_binding_protein,_putative	AT4G31880
TC09G025680	0.49	1.41	0.00	0.005	Nucleic_acid_binding_protein,_putative	AT5G66730
TC08G007190	0.49	1.41	0.01	0.043	Putative_Group_XV_phospholipase_A2	AT1G27480
TC02G014240	0.49	1.41	0.01	0.041	Predicted_protein	AT3G21890
TC10G009100	0.49	1.40	0.00	0.004	Putative_uncharacterized_protein	NA
TC02G034460	0.49	1.40	0.00	0.000	ATP-dependent_Clp_protease_adapter_protein_clpS	AT1G68660
TC03G001040	0.49	1.40	0.00	0.008	Putative_Protein_EXECUTER_1,_chloroplastic	AT1G27510
TC00G089400	0.49	1.40	0.00	0.001	Isoamylase_1,_chloroplastic	AT2G39930
TC03G003190	0.48	1.40	0.00	0.001	Putative_uncharacterized_protein	AT1G68490
TC01G037720	0.48	1.40	0.00	0.000	Predicted_protein	AT2G45380
TC05G029630	0.48	1.40	0.00	0.035	Putative_Cation/calcium_exchanger_4	AT2G24960
TC02G030700	0.48	1.39	0.00	0.010	Aconitate_hydratase_2,_mitochondrial	AT2G05710
TC05G028270	0.48	1.39	0.00	0.033	Acyltransferase-like_protein_At1g54570,_chloroplastic	AT1G54570
TC08G008390	0.48	1.39	0.01	0.042	Hypothetical_protein	NA
TC01G010150	0.47	1.39	0.00	0.014	ABC_transporter_F_family_member_5	AT5G64840
TC03G012980	0.47	1.39	0.00	0.018	Putative_Dof_zinc_finger_protein_DOF5.4	AT5G60850
TC02G010610	0.47	1.39	0.00	0.001	Protein_EXECUTER_1,_chloroplastic	AT4G33630
TC05G028260	0.47	1.39	0.00	0.003	Putative_WD_repeat-containing_protein_48	AT3G05090
TC05G023570	0.47	1.39	0.00	0.020	Putative_High_affinity_cationic_amino_acid_transporter_1	AT1G58030
TC05G001000	0.47	1.39	0.00	0.000	Uncharacterized_tatC-like_protein_ymf16	ATMG00070

TC02G020210	0.47	1.39	0.00	0.000	REF/SRPP-like_protein_At1g67360	AT1G67360
TC08G003310	0.47	1.38	0.00	0.013	Putative_Protein_IQ-DOMAIN_32	AT1G19870
TC01G002070	0.47	1.38	0.00	0.004	Putative_uncharacterized_protein	AT4G38960
TC02G015630	0.47	1.38	0.00	0.036	Putative_uncharacterized_protein	AT4G28260
TC04G015180	0.47	1.38	0.00	0.000	ERD15	AT2G41430
TC06G009310	0.47	1.38	0.01	0.048	Glutamate_dehydrogenase,_putative	AT1G51720
TC10G003400	0.46	1.38	0.01	0.044	Heat_shock_cognate_70_kDa_protein	AT3G09440
TC09G028840	0.46	1.38	0.00	0.028	Arogenate_dehydratase/prephenate_dehydratase_6,_chloroplastic	AT1G08250
TC09G002180	0.46	1.37	0.00	0.012	Putative_clathrin_assembly_protein_At2g25430	AT2G25430
TC06G019930	0.46	1.37	0.00	0.029	Plastid-lipid-associated_protein,_chloroplastic	AT4G22240
TC03G003480	0.45	1.37	0.00	0.002	Putative_Protein_sel-1_homolog_2	AT1G18260
TC10G002710	0.45	1.37	0.00	0.037	Putative_Telomere-binding_protein_1	AT5G13820
TC00G035750	0.45	1.37	0.01	0.048	Putative_ALG-2_interacting_protein_X	AT1G15130
TC04G003700	0.45	1.36	0.00	0.021	Putative_membrane_protein_ycf1	ATCG01130
TC08G004940	0.45	1.36	0.00	0.000	1,4-alpha-glucan-branching_enzyme	AT2G36390
TC00G081420	0.44	1.36	0.00	0.039	Remorin,_putative	AT2G41870
TC01G004540	0.44	1.36	0.00	0.030	Putative_uncharacterized_protein	AT2G03500
TC09G034320	0.44	1.36	0.01	0.049	Pentatricopeptide_repeat-containing_protein_At2g30100,_chloroplastic	AT2G30100
TC02G014630	0.44	1.36	0.00	0.010	Putative_uncharacterized_protein	AT4G27310
TC06G016270	0.44	1.36	0.00	0.036	NA	NA
TC09G007820	0.44	1.35	0.01	0.044	NA	NA
TC02G000970	0.44	1.35	0.00	0.000	DNA_damage-binding_protein_1	AT4G05420
TC02G027730	0.44	1.35	0.00	0.029	Myosin_heavy_chain,_clone,_putative	AT1G14840
TC02G034700	0.43	1.35	0.00	0.019	Predicted_protein	NA
TC04G004120	0.43	1.35	0.00	0.003	Probable_metal-nicotianamine_transporter_YSL6	AT3G27020
TC01G002780	0.43	1.35	0.00	0.013	Putative_uncharacterized_protein	AT5G66450
TC01G040610	0.43	1.35	0.00	0.000	Carotenoid_9,10(9',10')-cleavage_dioxygenase_1	AT3G63520
TC08G002030	0.43	1.34	0.00	0.000	Predicted_protein	AT1G75180
TC04G010110	0.42	1.34	0.00	0.027	Chaperone_protein_clpB_2	AT5G15450
TC00G064020	0.42	1.34	0.00	0.000	NA	NA
TC05G020530	0.42	1.34	0.00	0.019	DEAD-box_ATP-dependent_RNA_helicase_37	AT2G42520
TC09G012470	0.42	1.34	0.01	0.043	NA	NA
TC09G033150	0.42	1.34	0.00	0.000	Protein_ETHYLENE_INSENSITIVE_3	AT3G20770
TC10G014650	0.42	1.34	0.00	0.001	NA	NA
TC00G062210	0.42	1.34	0.00	0.035	Predicted_protein	AT1G73460
TC03G018200	0.42	1.34	0.01	0.048	Beta-fructofuranosidase,_soluble_isoenzyme_I	AT1G12240
TC01G036980	0.42	1.33	0.00	0.015	Putative_Peroxisomal_membrane_protein_PEX16	NA

TC05G020130	0.42	1.33	0.00	0.035	Ubiquitin_carboxyl-terminal_hydrolase_12	AT3G11910
TC06G003340	0.42	1.33	0.00	0.003	Pyrophosphate-energized_vacuolar_membrane_proton_pump	AT1G15690
TC09G022990	0.42	1.33	0.00	0.000	Predicted_protein	AT5G12950
TC03G010330	0.41	1.33	0.00	0.029	Probable_methyltransferase_PMT2	AT1G26850
TC04G019150	0.41	1.33	0.00	0.003	Putative_uncharacterized_protein	AT3G59430
TC02G006270	0.41	1.33	0.00	0.011	DNA_binding_protein,_putative	AT4G38900
TC06G019040	0.41	1.33	0.00	0.025	UPF0051_protein_ABCI8,_chloroplastic	AT4G04770
TC02G010520	0.41	1.33	0.00	0.000	Putative_glutamine_amidotransferase_YLR126C	AT2G23970
TC07G000930	0.41	1.32	0.00	0.035	Putative_uncharacterized_protein	AT4G03090
TC01G000560	0.40	1.32	0.00	0.023	Catalase_isozyme_2	AT4G35090
TC08G005260	0.40	1.32	0.00	0.001	Glyceraldehyde-3-phosphate_dehydrogenase_B,_chloroplastic	AT1G42970
TC01G006260	0.40	1.32	0.00	0.000	Putative_BTB/POZ_domain-containing_protein_At5g48800	AT5G67385
TC06G019120	0.40	1.32	0.00	0.018	Predicted_protein	AT1G11700
TC09G014930	0.40	1.32	0.00	0.002	Peptidyl-prolyl_cis-trans_isomerase_cyp5	AT4G32420
TC04G024320	0.40	1.32	0.00	0.002	1,2-dihydroxy-3-keto-5-methylthiopentene_dioxygenase_4	AT5G43850
TC01G027890	0.39	1.31	0.00	0.024	Putative_Protein_of_unknown_function_DUF926	AT4G02720
TC04G017390	0.39	1.31	0.00	0.020	Stromal_70_kDa_heat_shock-related_protein,_chloroplastic	AT5G49910
TC07G014720	0.39	1.31	0.00	0.009	Predicted_protein	AT1G79090
TC02G023910	0.38	1.31	0.00	0.008	Naphthoate_synthase	AT1G60550
TC03G020070	0.38	1.30	0.00	0.009	Scarecrow-like_protein_8	AT5G52510
TC03G006490	0.38	1.30	0.00	0.023	Transcription_factor_Myb1	AT1G19000
TC00G017640	0.38	1.30	0.00	0.000	Putative_97_kDa_heat_shock_protein	AT1G79930
TC07G017290	0.38	1.30	0.00	0.004	Succinate-semialdehyde_dehydrogenase,_mitochondrial	AT1G79440
TC09G033490	0.38	1.30	0.00	0.000	Putative_uncharacterized_protein	AT1G07990
TC09G001730	0.38	1.30	0.01	0.041	Probable_inosine-5'-monophosphate_dehydrogenase	AT1G16350
TC08G004960	0.38	1.30	0.00	0.008	Predicted_protein	AT2G30600
TC03G011030	0.38	1.30	0.01	0.043	Geranylgeranyl_diphosphate_reductase,_chloroplastic	AT1G74470
TC04G023490	0.38	1.30	0.00	0.000	Lipoxygenase_5,_chloroplastic	AT3G22400
TC01G027440	0.38	1.30	0.00	0.012	DNA_repair_helicase_UVH6	AT1G03190
TC01G008030	0.37	1.29	0.00	0.001	Putative_uncharacterized_protein	AT5G10490
TC02G032380	0.37	1.29	0.01	0.047	Probable_inactive_purple_acid_phosphatase_2	AT1G13900
TC04G014850	0.37	1.29	0.00	0.012	Putative_KH_domain-containing_protein_At4g18375	NA
TC07G010350	0.37	1.29	0.01	0.043	Predicted_protein	AT5G54830
TC09G022300	0.36	1.29	0.00	0.000	Lipoxygenase_2,_chloroplastic	AT3G45140
TC06G001640	0.36	1.29	0.00	0.002	Phospholipase_D_alpha_1	AT1G52570

TC03G025820	0.36	1.29	0.00	0.028	Primary_amine_oxidase	AT4G12290
TC08G001240	0.36	1.29	0.00	0.016	Molybdopterin_cofactor_sulfurase,_putative	AT2G23520
TC04G029780	0.36	1.29	0.01	0.047	Transcription_factor_bHLH148	AT3G06590
TC09G022580	0.36	1.28	0.00	0.036	Lipoxygenase_2,_chloroplastic	AT1G17420
TC01G030890	0.36	1.28	0.00	0.001	Sucrose_synthase_2	AT4G02280
TC05G000810	0.36	1.28	0.00	0.011	Putative_uncharacterized_protein	NA
TC03G023110	0.36	1.28	0.00	0.005	Transcription_factor_MYC2	AT4G17880
TC09G022240	0.36	1.28	0.00	0.001	Actin-related_protein_4	AT1G18450
TC09G000280	0.36	1.28	0.01	0.042	Galactokinase,_putative	AT4G16130
TC04G015710	0.36	1.28	0.01	0.049	Protein_binding_protein,_putative	AT5G01960
TC02G012930	0.36	1.28	0.00	0.012	Sphingoid_long-chain_bases_kinase_1	AT5G23450
TC05G010830	0.36	1.28	0.00	0.021	BEL1-like_homeodomain_protein_1	AT2G35940
TC06G016740	0.36	1.28	0.01	0.049	Pre-mRNA-splicing_factor_SYF1	AT5G28740
TC00G033720	0.35	1.28	0.00	0.000	Putative_Protein_thiJ	AT1G53280
TC02G028560	0.35	1.28	0.00	0.015	Putative_Probable_ADP-ribosylation_factor_GTPase-activating_protein_AGD11	AT1G70790
TC10G011790	0.35	1.27	0.00	0.000	DnaJ_protein_homolog	AT3G44110
TC09G013050	0.35	1.27	0.01	0.048	Serine/threonine_protein_phosphatase_2A_57_kDa_regulatory_subunit_B'_iota_isoform	AT5G25510
TC00G009420	0.35	1.27	0.00	0.027	Putative_uncharacterized_protein	AT5G22120
TC00G037930	0.35	1.27	0.00	0.001	Hypothetical_protein	AT4G25340
TC09G000780	0.34	1.27	0.01	0.048	Probable_WRKY_transcription_factor_11	AT2G24570
TC00G033840	0.34	1.27	0.00	0.009	Predicted_protein	AT5G16260
TC09G032890	0.34	1.27	0.00	0.000	Phototropin-1	AT3G45780
TC02G032180	0.34	1.27	0.00	0.008	Predicted_protein	AT4G09350
TC02G012690	0.34	1.26	0.01	0.041	NA	NA
TC00G089630	0.34	1.26	0.00	0.002	ATPase_11,_plasma_membrane-type	AT5G62670
TC00G051020	0.34	1.26	0.00	0.000	Putative_uncharacterized_protein	AT1G43700
TC01G040260	0.33	1.26	0.00	0.008	Gd2b,_putative	AT5G13760
TC04G026120	0.33	1.26	0.00	0.017	Putative_uncharacterized_protein	AT5G13890
TC04G018640	0.33	1.26	0.01	0.047	NA	NA
TC09G029150	0.33	1.26	0.00	0.010	At5g22830	AT5G22830
TC09G017990	0.33	1.25	0.00	0.000	Adagio_protein_1	AT5G57360
TC05G031960	0.33	1.25	0.00	0.011	NA	NA
TC01G009250	0.32	1.25	0.00	0.038	Putative_Ethylene-responsive_transcription_factor_ERF060	AT4G39780
TC05G026200	0.32	1.25	0.00	0.005	Predicted_protein	AT1G54450
TC07G002930	0.32	1.25	0.00	0.033	Aspartic_proteinase	AT1G11910
TC04G023040	0.32	1.25	0.00	0.012	Putative_Stem-specific_protein_TSJT1	AT5G19140
TC09G013770	0.32	1.25	0.00	0.005	Polyol_transporter_5	AT3G18830
TC01G000990	0.31	1.24	0.00	0.025	Predicted_protein	AT4G35780

TC03G020770	0.31	1.24	0.00	0.004	Putative_uncharacterized_protein	AT5G52230
TC01G018610	0.31	1.24	0.00	0.028	Zinc_finger_CCCH_domain-containing_protein_18	AT2G05160
TC06G001480	0.31	1.24	0.00	0.000	Putative_uncharacterized_protein	AT5G49820
TC01G040090	0.31	1.24	0.00	0.017	Predicted_protein	AT3G63460
TC02G022530	0.31	1.24	0.00	0.028	Probable_beta-1,3-galactosyltransferase_19	AT5G62620
TC09G009310	0.31	1.24	0.01	0.047	Hypothetical_protein	AT1G65660
TC04G029190	0.31	1.24	0.00	0.021	Calmodulin-binding_transcription_activator_5	AT3G16940
TC02G011710	0.31	1.24	0.00	0.000	Serine/arginine_rich_splicing_factor,_putative	AT5G64200
TC09G010360	0.31	1.24	0.00	0.009	Myristoyl-acyl_carrier_protein_thioesterase,_chloroplastic	AT1G08510
TC02G009740	0.31	1.24	0.00	0.028	Predicted_protein	NA
TC01G006960	0.31	1.24	0.01	0.049	Squalene_monooxygenase	AT4G37760
TC01G003540	0.31	1.24	0.00	0.001	Probable_protein_phosphatase_2C_80	AT4G16580
TC09G002870	0.31	1.24	0.00	0.020	CBL-interacting_serine/threonine-protein_kinase_6	AT4G30960
TC01G030450	0.31	1.24	0.00	0.009	DNA_binding_protein,_putative	AT2G47700
TC09G014720	0.30	1.24	0.01	0.041	Eukaryotic_initiation_factor_iso-4F_subunit_p82-34	AT2G24050
TC09G017350	0.30	1.23	0.00	0.038	NADPH--cytochrome_P450_reductase	AT4G24520
TC01G040660	0.30	1.23	0.00	0.004	Zinc_finger_CCCH_domain-containing_protein_30	AT2G41900
TC03G021030	0.30	1.23	0.00	0.000	PP2A_regulatory_subunit_TAP46	AT5G53000
TC01G020450	0.30	1.23	0.00	0.000	Putative_uncharacterized_protein	AT5G48385
TC01G021920	0.30	1.23	0.00	0.008	Putative_G3BP-like_protein	NA
TC05G020520	0.30	1.23	0.00	0.000	Serine/threonine-protein_phosphatase_PP2A-3_catalytic_subunit	AT3G58500
TC09G033080	0.30	1.23	0.00	0.035	Serrate_RNA_effector_molecule	AT2G27100
TC09G004540	0.30	1.23	0.01	0.048	Putative_UPF0587_protein_C1orf123_homolog	AT4G32930
TC04G020340	0.29	1.23	0.00	0.001	Putative_selenium-binding_protein	AT4G14030
TC07G011910	0.29	1.23	0.00	0.029	Secologanin_synthase	AT3G14690
TC05G002480	0.29	1.23	0.00	0.011	Ubiquitin-protein_ligase,_putative	AT5G01450
TC03G030630	0.29	1.22	0.00	0.016	Uncharacterized_protein_At4g19112.1	AT4G19110
TC01G038370	0.29	1.22	0.00	0.008	Transcription_factor_UNE10	AT4G00050
TC01G015990	0.29	1.22	0.00	0.012	Putative_Allene_oxide_synthase,_chloroplastic	AT4G15440
TC00G088660	0.29	1.22	0.01	0.040	Putative_Intracellular_protease_1	AT3G02720
TC03G014790	0.29	1.22	0.00	0.030	Putative_uncharacterized_protein	AT5G50380
TC09G008570	0.29	1.22	0.00	0.000	6-phosphofructokinase_3	AT4G26270
TC00G016810	0.29	1.22	0.00	0.000	Ring_finger_protein,_putative	NA
TC09G005830	0.29	1.22	0.00	0.000	Predicted_protein	AT2G26280
TC03G014110	0.28	1.22	0.00	0.036	Zinc_finger_CCCH_domain-containing_protein_43	AT5G63260
TC08G011990	0.28	1.22	0.00	0.000	F-box/LRR-repeat_protein_5	AT1G21410

TC05G014010	0.28	1.21	0.00	0.000	Putative_TGACG-sequence-specific_DNA-binding_protein_TGA-1B_(Fragment)	AT2G40950
TC09G033720	0.28	1.21	0.00	0.026	RuBisCO_large_subunit-binding_protein_subunit_alpha,_chloroplastic	AT2G28000
TC01G029580	0.28	1.21	0.00	0.000	Tubby-like_F-box_protein_3	AT2G47900
TC05G031720	0.28	1.21	0.00	0.001	Predicted_protein	AT3G05830
TC04G030560	0.27	1.21	0.00	0.007	Putative_[Pyruvate_dehydrogenase_[lipoamide]]_kinase,_mitochondrial	AT3G06483
TC01G037650	0.27	1.21	0.00	0.000	Putative_Dihydroflavonol-4-reductase	AT2G45400
TC00G035090	0.27	1.21	0.01	0.045	26S_proteasome_non-ATPase_regulatory_subunit_2	AT2G20580
TC08G010740	0.27	1.21	0.01	0.044	Predicted_protein	AT1G21660
TC01G013340	0.27	1.21	0.00	0.011	PHD_finger_protein_At1g33420	AT1G33420
TC01G021990	0.27	1.21	0.00	0.037	Predicted_protein	AT3G25120
TC02G029750	0.27	1.21	0.00	0.032	Probable_xyloglucan_endotransglucosylase/hydrolase_protein_28	AT1G14720
TC06G010070	0.27	1.20	0.00	0.032	Protein_phosphatase_2C_16	AT1G72770
TC00G092360	0.27	1.20	0.00	0.016	Putative_uncharacterized_protein	AT3G22970
TC10G007040	0.26	1.20	0.00	0.001	NAC_domain-containing_protein_78	AT5G04410
TC08G001680	0.26	1.20	0.00	0.000	Metalloendopeptidase,_putative	AT5G42620
TC06G019520	0.26	1.20	0.00	0.000	Aspartic_proteinase	AT1G11910
TC09G002670	0.26	1.20	0.00	0.011	Probable_cyclic_nucleotide-gated_ion_channel_5	AT2G23980
TC08G001250	0.26	1.20	0.00	0.036	Putative_B3_domain-containing_protein_Os11g0197600	AT4G34400
TC03G030120	0.26	1.19	0.00	0.002	Pentatricopeptide_repeat-containing_protein_At1g31920	AT1G31920
TC00G056860	0.26	1.19	0.00	0.001	Putative_Zinc_finger_protein_CONSTANS-LIKE_16	AT1G25440
TC05G019370	0.25	1.19	0.00	0.011	Thioredoxin_F-type_2,_chloroplastic	AT3G02730
TC01G022350	0.25	1.19	0.00	0.021	Transcriptional_corepressor_LEUNIG	AT2G32700
TC03G017290	0.25	1.19	0.00	0.001	Putative_Splicing_factor_3A_subunit_3	AT5G06160
TC04G008190	0.25	1.19	0.00	0.003	Probable_inactive_receptor_kinase_At1g27190	AT1G27190
TC08G008900	0.25	1.19	0.00	0.031	Probable_pectin_methyltransferase_QUA2	AT1G78240
TC08G002400	0.25	1.19	0.00	0.000	Mannan_endo-1,4-beta-mannosidase_7	AT5G66460
TC02G002990	0.25	1.19	0.00	0.000	Predicted_protein	AT5G24690
TC00G028320	0.25	1.19	0.00	0.036	Beta-glucosidase_42	AT5G36890
TC00G029800	0.25	1.19	0.00	0.001	Serine/threonine-protein_phosphatase_PP2A_catalytic_subunit	AT3G58500
TC06G007960	0.25	1.19	0.00	0.015	Probable_esterase_At1g33990	AT1G33990
TC04G030270	0.25	1.19	0.00	0.012	Yth_domain-containing_protein,_putative	AT1G48110
TC00G075330	0.25	1.19	0.00	0.029	Serine/threonine-protein_phosphatase_PP2A_catalytic_subunit	AT1G10430
TC06G015290	0.24	1.18	0.00	0.000	Triose_phosphate/phosphate_translocator,_chloroplastic	AT5G46110
TC09G025520	0.24	1.18	0.00	0.004	CBL-interacting_serine/threonine-protein_kinase_3	AT2G26980
TC05G015330	0.24	1.18	0.00	0.020	Putative_uncharacterized_protein	AT3G10250

TC01G005040	0.24	1.18	0.00	0.038	Splicing_factor,_arginine/serine-rich,_putative	AT4G36980
TC04G025230	0.24	1.18	0.00	0.022	NA	NA
TC03G027640	0.24	1.18	0.00	0.000	Putative_Aminopeptidase_N	AT1G63770
TC01G012600	0.24	1.18	0.00	0.006	Putative_Neutral_alpha-glucosidase_AB	AT5G63840
TC00G023330	0.23	1.18	0.00	0.036	37_kDa_inner_envelope_membrane_protein,_chloroplastic	AT3G63410
TC04G022640	0.23	1.18	0.00	0.009	Catalytic,_putative	AT4G14290
TC03G025700	0.23	1.17	0.00	0.013	Putative_Hsc70-interacting_protein	AT4G22670
TC09G000110	0.23	1.17	0.00	0.009	Putative_uncharacterized_protein	AT2G24360
TC06G007940	0.23	1.17	0.00	0.027	VHS_domain-containing_protein_At3g16270	AT3G16270
TC04G028120	0.23	1.17	0.00	0.038	Probable_histone-arginine_methyltransferase_CARM1B	AT3G06930
TC05G000050	0.23	1.17	0.00	0.024	Lupus_la_ribonucleoprotein,_putative	AT2G43970
TC05G028520	0.23	1.17	0.00	0.022	Casein_kinase,_putative	AT3G03940
TC02G034690	0.23	1.17	0.01	0.042	Protein_CHUP1,_chloroplastic	AT3G25690
TC00G044800	0.23	1.17	0.00	0.001	F-box_protein_At1g47056	AT3G50080
TC07G000040	0.23	1.17	0.01	0.040	Probable_calcium-binding_protein_CML21	AT4G26470
TC02G014990	0.23	1.17	0.00	0.005	Protein_THYLAKOID_FORMATION_1,_chloroplastic	AT2G20890
TC00G019720	0.22	1.17	0.00	0.031	Putative_serine/threonine-protein_kinase_receptor	AT2G19130
TC04G023260	0.22	1.16	0.00	0.031	Protein_phosphatase_2C_70	AT5G19280
TC01G003290	0.22	1.16	0.00	0.000	Scarecrow-like_protein_4	AT5G66770
TC09G008250	0.21	1.16	0.00	0.023	Xaa-Pro_dipeptidase	AT4G29490
TC10G001470	0.21	1.16	0.01	0.041	Phosphoinositide_phospholipase_C_2	AT3G08510
TC08G013910	0.21	1.16	0.01	0.049	Putative_TVP38/TMEM64_family_membrane_protein_slr0305	AT1G22850
TC03G002290	0.21	1.16	0.00	0.000	Elongation_factor_Tu,_chloroplastic	AT4G20360
TC09G010660	0.21	1.15	0.00	0.003	Serine-threonine_kinase_receptor-associated_protein	AT1G15470
TC09G025090	0.20	1.15	0.00	0.010	F-box_protein_At2g32560	AT2G26850
TC05G014020	0.20	1.15	0.00	0.005	Predicted_protein	AT2G40960
TC03G014050	0.20	1.15	0.01	0.043	Putative_Programmed_cell_death_protein_4	AT4G24800
TC03G019640	0.20	1.15	0.01	0.047	Predicted_protein	AT4G25520
TC04G023960	0.20	1.15	0.01	0.048	Transporter,_putative	AT2G32040
TC02G005290	0.20	1.15	0.01	0.042	Protein_translocase,_putative	AT3G16620
TC00G022300	0.20	1.15	0.00	0.039	Putative_uncharacterized_protein	AT2G07360
TC08G001020	0.20	1.15	0.00	0.015	Predicted_protein	AT5G36210
TC06G013220	0.20	1.15	0.01	0.050	Putative_uncharacterized_protein	AT1G29370
TC08G001760	0.20	1.15	0.00	0.007	Predicted_protein	AT1G80950
TC06G001720	0.20	1.15	0.00	0.021	Nucleosome-binding_protein,_putative	AT1G15340
TC00G004350	0.20	1.15	0.01	0.048	Putative_Protein_CHLOROPLAST_IMPORT_APPARATUS_2	AT4G25990
TC00G037090	0.20	1.15	0.00	0.000	Putative_uncharacterized_protein	AT1G70160
TC10G000200	0.20	1.15	0.00	0.008	Putative_Zinc_finger_CCCH_domain-	AT3G51950

					containing_protein_53	
TC01G002900	0.19	1.14	0.01	0.041	Putative_uncharacterized_protein	AT3G50910
TC00G014550	0.19	1.14	0.00	0.017	Predicted_protein	AT5G61960
TC00G059940	0.19	1.14	0.00	0.002	Cytochrome_c1-1,_heme_protein,_mitochondrial	AT5G40810
TC01G006660	0.19	1.14	0.00	0.028	Zinc_finger_CCCH_domain-containing_protein_49	AT1G07360
TC04G004860	0.19	1.14	0.00	0.032	Protein_AUXIN_SIGNALING_F-BOX_2	AT1G12820
TC09G029990	0.18	1.14	0.00	0.013	NA	NA
TC07G000110	0.18	1.13	0.00	0.013	Putative_Uncharacterized_WD_repeat-containing_protein_C2A9.03	AT1G55680
TC08G005720	0.18	1.13	0.00	0.007	Glucose-6-phosphate_isomerase	AT4G24620
TC03G020210	0.17	1.12	0.00	0.035	Putative_transporter_arsB	AT1G02260
TC04G014440	0.16	1.12	0.00	0.020	Plastidic_glucose_transporter_4	AT5G16150
TC03G028900	0.16	1.12	0.00	0.022	Putative_Fatty_acyl-CoA_synthetase_A	AT4G23850
TC04G022810	0.16	1.12	0.00	0.018	Predicted_protein	AT4G14240
TC02G008970	0.16	1.12	0.00	0.001	Protein_binding_protein,_putative	AT4G34040
TC01G018000	0.16	1.12	0.00	0.004	Putative_Ubiquitin_and_WLM_domain-containing_protein_C1442.07c	AT5G35690
TC02G034730	0.15	1.11	0.00	0.000	Predicted_protein	AT1G26110
TC04G013930	0.15	1.11	0.00	0.039	Putative_L-Ala-D/L-Glu_epimerase	AT3G18270
TC05G005830	0.13	1.09	0.00	0.010	Predicted_protein	NA
TC01G026730	0.13	1.09	0.00	0.033	Transcription_factor_bHLH3	AT4G16430
TC01G034790	0.10	1.07	0.01	0.048	Putative_Mitochondrial_substrate_carrier_family_protein_B	AT4G01100
TC00G080870	-0.11	-1.08	0.01	0.040	Putative_uncharacterized_protein	AT5G01010
TC03G014720	-0.12	-1.08	0.00	0.009	Putative_uncharacterized_protein	AT5G63440
TC03G014630	-0.12	-1.09	0.00	0.024	Argininosuccinate_synthase,_chloroplastic	AT4G24830
TC04G019020	-0.13	-1.10	0.00	0.018	Putative_Probable_importin-7_homolog	AT2G31660
TC02G011840	-0.13	-1.10	0.00	0.020	Putative_uncharacterized_protein	AT5G64160
TC00G019560	-0.14	-1.10	0.01	0.041	Predicted_protein_(Fragment)	AT1G15420
TC09G007780	-0.14	-1.10	0.01	0.043	Putative_Translation_machinery-associated_protein_22	AT5G11900
TC04G024750	-0.14	-1.10	0.00	0.035	40S_ribosomal_protein_S15	AT5G09510
TC04G002630	-0.15	-1.11	0.00	0.015	Putative_uncharacterized_protein	AT5G14310
TC08G013960	-0.15	-1.11	0.00	0.024	Bifunctional_aspartokinase/homoserine_dehydrogenase_2,_chloroplastic	AT1G31230
TC00G092380	-0.15	-1.11	0.00	0.030	ADP-ribosylation_factor-like_protein_5	AT3G22950
TC09G004320	-0.15	-1.11	0.01	0.045	Mitochondrial_import_inner_membrane_translocase_subunit_Tim17	AT2G37410
TC02G023160	-0.15	-1.11	0.00	0.031	Putative_Protein_LSM12_homolog	AT1G24050
TC01G009540	-0.16	-1.11	0.01	0.050	Putative_U-box_domain-containing_protein_13	AT2G01490
TC05G018910	-0.16	-1.11	0.00	0.036	Putative_uncharacterized_protein	AT5G13020
TC04G009070	-0.16	-1.11	0.01	0.045	AT3g57280/F28O9_130	AT3G57280
TC08G010320	-0.16	-1.12	0.00	0.030	Predicted_protein	AT4G08240
TC05G018850	-0.16	-1.12	0.00	0.001	Zinc_finger_CCCH_domain-containing_protein_14	AT3G12130

TC08G006080	-0.16	-1.12	0.00	0.018	Putative_uncharacterized_protein	AT5G65520
TC05G017360	-0.16	-1.12	0.00	0.029	Uncharacterized_protein_At2g41620	AT2G41620
TC10G001890	-0.17	-1.12	0.00	0.030	Syntaxin-31	AT5G05760
TC03G024520	-0.17	-1.12	0.00	0.032	UPF0326_protein_At4g17486	AT5G47310
TC04G028190	-0.17	-1.12	0.00	0.011	Kif4,_putative	AT4G15885
TC04G029650	-0.17	-1.12	0.01	0.043	Putative_3-hydroxybutyryl-CoA_dehydratase	AT4G16210
TC05G020730	-0.17	-1.13	0.01	0.039	Pentatricopeptide_repeat-containing_protein,_putative	AT5G36290
TC09G006690	-0.17	-1.13	0.00	0.030	Lsm1,_putative	AT3G14080
TC10G016730	-0.17	-1.13	0.00	0.023	60S_ribosomal_protein_L10	AT1G26910
TC05G017270	-0.17	-1.13	0.00	0.026	Transcription_initiation_factor_IIB-2	AT3G10330
TC04G002110	-0.17	-1.13	0.00	0.038	Putative_Pre-rRNA-processing_protein_ESF2	AT3G56510
TC07G012610	-0.18	-1.13	0.00	0.017	Putative_Ubiquitin_carboxyl-terminal_hydrolase_15	AT1G17110
TC07G012770	-0.18	-1.13	0.00	0.035	Ethylene_receptor	AT1G66340
TC03G019870	-0.18	-1.13	0.00	0.025	40S_ribosomal_protein_S10-1	AT4G25740
TC00G020830	-0.18	-1.13	0.00	0.022	Putative_Porin%2C_eukaryotic_type	AT5G57490
TC09G001360	-0.18	-1.13	0.00	0.035	Probable_protein_phosphatase_2C_60	AT4G31860
TC00G061280	-0.18	-1.13	0.00	0.036	Putative_Probable_methionyl-tRNA_synthetase	AT2G40660
TC09G008410	-0.18	-1.13	0.00	0.011	Asparaginyl-tRNA_synthetase,_cytoplasmic_1	AT1G70980
TC09G032010	-0.18	-1.13	0.01	0.048	Putative_Sec14_cytosolic_factor	AT3G46450
TC02G029990	-0.18	-1.13	0.00	0.027	Probable_small_nuclear_ribonucleoprotein_Sm_D2	AT3G62840
TC09G011850	-0.18	-1.13	0.00	0.018	Putative_uncharacterized_protein	AT5G32440
TC01G030330	-0.18	-1.13	0.00	0.015	Putative_Thiamin_pyrophosphokinase_1	AT2G44750
TC00G026740	-0.18	-1.13	0.00	0.021	Glutaminyl-tRNA_synthetase	AT1G25350
TC09G025160	-0.18	-1.13	0.00	0.002	Probable_diphthine_synthase	AT4G31790
TC06G014610	-0.18	-1.13	0.00	0.007	Predicted_protein	AT1G29040
TC09G003510	-0.18	-1.14	0.00	0.002	GTP-binding_protein_At2g22870	AT5G11480
TC08G008260	-0.18	-1.14	0.00	0.033	Squamosa_promoter-binding-like_protein_14	NA
TC04G024770	-0.18	-1.14	0.00	0.038	Xpa-binding_protein,_putative	AT4G21800
TC03G029930	-0.19	-1.14	0.00	0.036	Shikimate_kinase-like_protein	AT2G35500
TC03G024980	-0.19	-1.14	0.00	0.037	Asparaginyl-tRNA_synthetase,_chloroplastic/mitochondrial	AT4G17300
TC00G054340	-0.19	-1.14	0.00	0.011	116_kDa_U5_small_nuclear_ribonucleoprotein_component	AT5G25230
TC00G000380	-0.19	-1.14	0.00	0.003	Putative_uncharacterized_protein	NA
TC09G000140	-0.19	-1.14	0.00	0.026	Putative_endonuclease_FLJ39025	AT4G31150
TC00G012470	-0.19	-1.14	0.01	0.043	Ribosome_biogenesis_protein_NSA2_homolog	AT5G06360
TC01G035200	-0.19	-1.14	0.00	0.012	Golgin_candidate_4	AT2G46180
TC04G021120	-0.19	-1.14	0.01	0.049	ER_lumen_protein_retaining_receptor	AT3G25040
TC03G002590	-0.19	-1.14	0.00	0.019	Protein_CASP	AT3G18480

TC03G017430	-0.19	-1.14	0.01	0.048	Cyclic_nucleotide-gated_ion_channel_1	AT5G53130
TC08G008960	-0.20	-1.14	0.00	0.001	14-3-3_protein_7	AT1G34760
TC02G024000	-0.20	-1.14	0.00	0.036	Nucleic_acid_binding_protein,_putative	AT2G27790
TC01G033270	-0.20	-1.15	0.01	0.048	Putative_Cathepsin_B	AT1G02305
TC10G006430	-0.20	-1.15	0.00	0.028	Probable_exocyst_complex_component_4	AT3G10380
TC05G002830	-0.20	-1.15	0.00	0.001	Probable_26S_proteasome_non-ATPase_regulatory_subunit_7	AT3G11270
TC00G023340	-0.20	-1.15	0.00	0.000	Putative_uncharacterized_protein	AT3G63400
TC03G000380	-0.20	-1.15	0.00	0.010	Putative_Mannose-1-phosphate_guanyltransferase_alpha	AT1G74910
TC04G028720	-0.20	-1.15	0.00	0.014	Predicted_protein	AT5G11240
TC00G045330	-0.20	-1.15	0.01	0.047	Callose_synthase_9	AT3G07160
TC01G003610	-0.20	-1.15	0.00	0.001	Minor_allergen_Alt_a_7	AT4G36750
TC00G069990	-0.20	-1.15	0.00	0.000	Predicted_protein	AT2G26590
TC01G035490	-0.20	-1.15	0.01	0.045	CAAX_prenyl_protease_1_homolog	NA
TC09G008850	-0.20	-1.15	0.00	0.029	Hexokinase-1	AT2G19860
TC03G024770	-0.20	-1.15	0.01	0.045	NA	NA
TC10G010860	-0.20	-1.15	0.01	0.049	Nicastrin	AT3G52640
TC05G006520	-0.20	-1.15	0.00	0.005	Ribonucleoprotein_At2g37220,_chloroplastic	AT2G37220
TC02G006750	-0.20	-1.15	0.00	0.002	Putative_Isoflavone_reductase_homolog_A622	AT4G34540
TC01G010180	-0.20	-1.15	0.00	0.035	Putative_Cytochrome_P450_86B1	AT4G39490
TC02G034470	-0.20	-1.15	0.00	0.027	NA	NA
TC01G036550	-0.20	-1.15	0.00	0.005	Putative_Methionine_adenosyltransferase_2_subunit_beta	AT4G00560
TC00G035490	-0.20	-1.15	0.00	0.003	Putative_Uncharacterized_protein_RP120	AT5G22350
TC09G003050	-0.20	-1.15	0.00	0.009	Predicted_protein	AT4G30996
TC09G009380	-0.20	-1.15	0.00	0.035	Methyltransferase-like_protein_5	AT4G28830
TC05G008450	-0.21	-1.15	0.00	0.011	Fructose-bisphosphate_aldolase_cytoplasmic_isozyme	AT2G36460
TC03G029180	-0.21	-1.15	0.00	0.000	Predicted_protein	AT4G23940
TC05G015710	-0.21	-1.15	0.00	0.022	Predicted_protein	AT3G13540
TC01G039970	-0.21	-1.16	0.01	0.048	Acyl_carrier_protein,_mitochondrial	AT2G44620
TC04G019550	-0.21	-1.16	0.00	0.034	Putative_Flowering_time_control_protein_FPA	NA
TC10G004930	-0.21	-1.16	0.00	0.023	Putative_UPF0363_protein	AT5G63220
TC00G089790	-0.21	-1.16	0.00	0.030	tRNA-specific_adenosine_deaminase_2	AT1G48175
TC02G029760	-0.21	-1.16	0.01	0.039	NA	NA
TC08G007130	-0.21	-1.16	0.01	0.043	Predicted_protein	AT1G59520
TC09G002940	-0.21	-1.16	0.00	0.001	Probable_CCR4-associated_factor_1_homolog_7	AT1G80780
TC05G002500	-0.21	-1.16	0.00	0.014	GDSL_esterase/lipase_CPRD49	AT2G38180
TC01G028010	-0.21	-1.16	0.00	0.000	WD-repeat_protein,_putative	AT1G03110
TC01G027040	-0.21	-1.16	0.00	0.016	Ring_finger_protein,_putative	AT1G06770

TC02G011700	-0.21	-1.16	0.00	0.038	Potassium_transporter_7	AT5G09400
TC05G001470	-0.21	-1.16	0.00	0.035	F-box/LRR-repeat_protein_3	AT5G01720
TC00G014270	-0.21	-1.16	0.01	0.040	NA	NA
TC02G009820	-0.21	-1.16	0.00	0.033	Thioredoxin_domain-containing_protein_9_homolog	AT2G18990
TC09G013700	-0.21	-1.16	0.00	0.022	Hypothetical_protein	AT1G11750
TC00G011720	-0.21	-1.16	0.00	0.038	Probable_N(2),N(2)-dimethylguanosine_tRNA_methyltransferase_2	AT5G15810
TC06G016460	-0.22	-1.16	0.00	0.024	UPF0559_protein	AT1G61150
TC01G006480	-0.22	-1.16	0.00	0.018	Mitochondrial_outer_membrane_protein_porin_of_34_kDa	AT5G67500
TC01G039860	-0.22	-1.16	0.00	0.036	Putative_Eukaryotic_translation_initiation_factor_4G	AT3G60240
TC09G026930	-0.22	-1.16	0.00	0.002	Tryptophanyl-tRNA_synthetase,_cytoplasmic	AT3G04600
TC09G003820	-0.22	-1.16	0.00	0.004	Putative_Uncharacterized_protein_KIAA0090_homolog	AT5G11560
TC09G014980	-0.22	-1.16	0.00	0.028	Cytochrome_b-c1_complex_subunit_7	AT4G32470
TC05G018330	-0.22	-1.16	0.00	0.028	60S_ribosomal_protein_L6	AT1G74050
TC01G003740	-0.22	-1.16	0.00	0.020	Geranylgeranyl_pyrophosphate_synthase,_chloroplastic	AT4G36810
TC02G022670	-0.22	-1.16	0.00	0.004	Phospholipid-transporting_ATPase_3	AT1G59820
TC10G004680	-0.22	-1.17	0.00	0.006	Putative_Leukotriene_A-4_hydrolase	AT5G13520
TC04G012020	-0.22	-1.17	0.00	0.028	Putative_Sodium-coupled_neutral_amino_acid_transporter_3	AT3G30390
TC09G026910	-0.22	-1.17	0.00	0.027	Probable_NADH_dehydrogenase_[ubiquinone]_1_alpha_subcomplex_subunit_12	AT3G03100
TC05G015340	-0.22	-1.17	0.00	0.001	Cystathionine_beta-lyase,_chloroplastic	AT3G57050
TC03G017040	-0.22	-1.17	0.00	0.011	NA	NA
TC00G026230	-0.22	-1.17	0.00	0.020	Putative_Zinc_finger_HIT_domain-containing_protein_3	AT4G28820
TC07G002140	-0.22	-1.17	0.00	0.000	40S_ribosomal_protein_S8	AT5G20290
TC04G018490	-0.22	-1.17	0.00	0.015	Bromodomain_protein	AT1G05910
TC00G049090	-0.22	-1.17	0.01	0.050	Biotin/lipoyl_attachment	AT1G52670
TC05G005930	-0.22	-1.17	0.01	0.040	40S_ribosomal_protein_S23	AT5G02960
TC10G005050	-0.23	-1.17	0.00	0.015	Cyclin-L1-1	NA
TC01G010170	-0.23	-1.17	0.00	0.007	Putative_DNA-directed_RNA_polymerase_II_subunit_RPB4	AT5G09920
TC06G006840	-0.23	-1.17	0.00	0.024	Plastidic_ATP/ADP-transporter	AT1G15500
TC09G007210	-0.23	-1.17	0.00	0.031	Putative_Replication_protein_A_70_kDa_DNA-binding_subunit	AT2G06510
TC01G019290	-0.23	-1.17	0.00	0.003	Putative_uncharacterized_protein	AT5G48240
TC05G022350	-0.23	-1.17	0.00	0.004	Cellulose_synthase-like_protein_D3	AT3G03050
TC01G002980	-0.23	-1.17	0.01	0.040	Isoflavone_reductase_homolog_A622	AT4G39230
TC02G024400	-0.23	-1.17	0.00	0.014	NA	NA
TC08G011150	-0.23	-1.17	0.00	0.036	Protein_disulfide-isomerase	AT1G21750
TC04G002730	-0.23	-1.17	0.00	0.029	Ribosomal_protein_L2_(Fragment)	AT2G44065
TC04G024650	-0.23	-1.17	0.00	0.001	Putative_Mitochondrial_inner_membrane_magnesium_transporter_mrs2	AT5G09690

TC05G003070	-0.23	-1.17	0.00	0.022	Ataxin-3_homolog	AT3G54130
TC06G015070	-0.23	-1.17	0.01	0.042	28_kDa_heat-and_acid-stable_phosphoprotein,_putative	AT5G46020
TC04G028770	-0.23	-1.18	0.00	0.009	Eukaryotic_translation_initiation_factor_3_subunit,_putative	NA
TC02G010240	-0.23	-1.18	0.00	0.010	Predicted_protein	AT3G19650
TC03G023400	-0.23	-1.18	0.00	0.038	Protein_ABCI7,_chloroplastic	AT1G32500
TC01G040870	-0.23	-1.18	0.01	0.048	60S_ribosomal_protein_L31	AT5G56710
TC00G034250	-0.24	-1.18	0.01	0.040	Fasciclin-like_arabinogalactan_protein_17	NA
TC06G017680	-0.24	-1.18	0.00	0.036	Predicted_protein	AT1G71730
TC05G030440	-0.24	-1.18	0.00	0.005	Malignant_T_cell-amplified_sequence_1	AT1G09150
TC02G004980	-0.24	-1.18	0.00	0.006	DNA_binding_protein,_putative	AT2G16770
TC02G009410	-0.24	-1.18	0.00	0.038	D-3-phosphoglycerate_dehydrogenase,_chloroplastic	AT4G34200
TC04G013210	-0.24	-1.18	0.00	0.008	ATP-dependent_Clp_protease_proteolytic_subunit_3,_chloroplastic	AT1G66670
TC09G029210	-0.24	-1.18	0.00	0.023	Predicted_protein	AT1G08220
TC06G018700	-0.24	-1.18	0.00	0.001	Putative_Pentatricopeptide_repeat-containing_protein_At4g21170	AT4G21170
TC03G003730	-0.24	-1.18	0.00	0.011	Predicted_protein	NA
TC01G033350	-0.24	-1.18	0.00	0.000	Translocase_of_chloroplast_33,_chloroplastic	AT1G02280
TC01G029630	-0.24	-1.18	0.00	0.015	Putative_uncharacterized_protein	AT4G03180
TC01G000080	-0.24	-1.18	0.00	0.001	Putative_AP-3_complex_subunit_sigma-2	AT3G50860
TC06G004380	-0.24	-1.18	0.00	0.036	Transporter,_putative	AT1G79710
TC07G012860	-0.24	-1.18	0.00	0.001	Transcription_regulator,_putative	AT1G61730
TC08G008940	-0.24	-1.18	0.01	0.048	Predicted_protein	AT1G34780
TC06G012100	-0.24	-1.19	0.00	0.008	Auxin_response_factor_6	AT1G30330
TC08G005960	-0.25	-1.19	0.00	0.024	Argininosuccinate_lyase	AT5G10920
TC06G020140	-0.25	-1.19	0.00	0.029	Putative_uncharacterized_protein	AT3G01435
TC01G003060	-0.25	-1.19	0.00	0.000	Anaphase-promoting_complex_subunit_10	AT2G18290
TC03G001670	-0.25	-1.19	0.00	0.015	Putative_uncharacterized_protein	AT5G63050
TC01G030740	-0.25	-1.19	0.00	0.019	40S_ribosomal_protein_S4	AT5G58420
TC03G029450	-0.25	-1.19	0.00	0.000	ABC_transporter_F_family_member_3	AT1G64550
TC00G054510	-0.25	-1.19	0.00	0.018	40S_ribosomal_protein_S14	AT3G52580
TC01G016740	-0.25	-1.19	0.00	0.005	60S_ribosomal_protein_L18a	AT2G34480
TC06G019480	-0.25	-1.19	0.00	0.027	Bcr-associated_protein,_bap,_putative	AT1G11905
TC03G010290	-0.25	-1.19	0.01	0.048	Putative_Protein_SET	AT1G18800
TC06G012800	-0.26	-1.19	0.00	0.033	60S_ribosomal_protein_L18a	AT2G34480
TC01G036620	-0.26	-1.19	0.01	0.040	Acyl-CoA_thioesterase,_putative	AT3G61200
TC09G005450	-0.26	-1.19	0.01	0.047	Omega-amidase_NIT2-B	AT5G12040
TC10G009540	-0.26	-1.19	0.00	0.030	Uncharacterized_protein_At2g38710	AT2G38710
TC01G002480	-0.26	-1.19	0.00	0.031	50S_ribosomal_protein_L7/L12,_putative	AT4G36420

TC03G003140	-0.26	-1.19	0.00	0.036	Predicted_protein	NA
TC06G017640	-0.26	-1.20	0.00	0.025	50S_ribosomal_protein_L13	AT3G01790
TC03G023760	-0.26	-1.20	0.00	0.024	NA	NA
TC03G020090	-0.26	-1.20	0.00	0.002	Putative_Fibrillarin	AT4G25630
TC10G004670	-0.26	-1.20	0.00	0.001	FGFR1_oncogene_partner,_putative	AT3G55000
TC05G026750	-0.26	-1.20	0.00	0.017	60S_ribosomal_protein_L10a-3	AT1G08360
TC04G028630	-0.26	-1.20	0.00	0.031	60S_ribosomal_protein_L19-2	AT1G02780
TC01G038820	-0.26	-1.20	0.00	0.001	Predicted_protein	NA
TC02G010560	-0.26	-1.20	0.00	0.023	Predicted_protein	AT3G21175
TC02G002800	-0.26	-1.20	0.00	0.012	Putative_Protein_RMD5_homolog_A	AT5G09630
TC05G025270	-0.26	-1.20	0.00	0.031	Predicted_protein	AT1G73177
TC02G008740	-0.26	-1.20	0.00	0.003	Putative_TP53RK-binding_protein	AT4G34412
TC05G014990	-0.26	-1.20	0.00	0.012	Transcription_factor_BTF3	AT1G73230
TC10G005060	-0.27	-1.20	0.00	0.005	Elongation_factor_1-alpha	AT5G60390
TC08G003640	-0.27	-1.20	0.00	0.000	Tubulin_beta-6_chain	AT1G75780
TC01G025590	-0.27	-1.20	0.00	0.001	Succinyl-CoA_ligase_[GDP-forming]_subunit_beta,_mitochondrial	AT2G20420
TC05G000450	-0.27	-1.21	0.00	0.011	Putative_ALBINO3-like_protein_2,_chloroplastic	AT3G44370
TC09G032400	-0.27	-1.21	0.00	0.029	60S_ribosomal_protein_L35	AT5G02610
TC08G005230	-0.27	-1.21	0.00	0.016	Glutamate_receptor_3.3	NA
TC02G000670	-0.27	-1.21	0.00	0.036	Hypothetical_protein	NA
TC07G002720	-0.28	-1.21	0.00	0.024	Aspartate_carbamoyltransferase_2,_chloroplastic	AT3G20330
TC09G026420	-0.28	-1.21	0.01	0.050	Elongation_factor_1-alpha	AT1G07930
TC00G026820	-0.28	-1.21	0.01	0.047	Putative_uncharacterized_protein	NA
TC10G002550	-0.28	-1.21	0.00	0.011	Putative_Folylpolyglutamate_synthase,_mitochondrial	AT3G55630
TC03G002720	-0.28	-1.21	0.00	0.010	Putative_F-box/WD_repeat-containing_protein_7	AT1G49040
TC00G015780	-0.28	-1.21	0.00	0.005	60S_ribosomal_protein_L35	AT3G09500
TC03G013550	-0.28	-1.21	0.00	0.010	50S_ribosomal_protein_L19,_putative	AT4G11630
TC09G022160	-0.28	-1.21	0.00	0.001	Predicted_protein	AT3G57930
TC00G013740	-0.28	-1.21	0.00	0.024	40S_ribosomal_protein_S9-2	AT5G39850
TC08G002520	-0.28	-1.21	0.01	0.047	NA	NA
TC09G011260	-0.28	-1.22	0.00	0.032	Predicted_protein	AT5G15050
TC07G002420	-0.29	-1.22	0.00	0.014	Putative_Casein_kinase_I_isoform_delta-like	AT3G23340
TC01G011220	-0.29	-1.22	0.00	0.005	Uridylate_kinase	AT3G18680
TC08G001140	-0.29	-1.22	0.00	0.024	Serpin-ZX	AT1G47710
TC00G025180	-0.29	-1.22	0.00	0.013	Putative_uncharacterized_protein	AT5G17210
TC09G008940	-0.29	-1.22	0.00	0.013	Putative_uncharacterized_protein	AT3G21600
TC01G025360	-0.29	-1.22	0.01	0.050	Probable_methyltransferase_PMT8	AT3G23300
TC03G027490	-0.29	-1.22	0.00	0.007	Predicted_protein	AT4G23330

TC06G003160	-0.29	-1.22	0.00	0.000	Putative_Checkpoint_protein_HUS1	AT1G52530
TC01G024270	-0.29	-1.22	0.00	0.038	Eukaryotic_peptide_chain_release_factor_subunit_1-3	AT3G26618
TC05G021520	-0.29	-1.22	0.00	0.010	At1g09815	AT1G09815
TC02G020400	-0.29	-1.22	0.00	0.000	Putative_uncharacterized_protein	AT3G56820
TC08G002870	-0.29	-1.22	0.00	0.031	Auxin-induced_protein_5NG4	AT1G75500
TC03G005730	-0.29	-1.22	0.01	0.041	Putative_Protein_phosphatase_inhibitor	AT2G31305
TC00G020790	-0.29	-1.22	0.00	0.036	Transcription_factor,_putative	AT3G13040
TC02G003710	-0.29	-1.22	0.01	0.040	LysM_domain-containing_GPI-anchored_protein_2	AT2G17120
TC04G025930	-0.29	-1.23	0.00	0.025	50S_ribosomal_protein_L28,_chloroplastic	AT2G33450
TC01G007950	-0.29	-1.23	0.00	0.027	Nucleobase-ascorbate_transporter_11	AT4G38050
TC01G008500	-0.30	-1.23	0.00	0.026	Putative_LanC-like_protein_2	AT5G65280
TC05G031090	-0.30	-1.23	0.00	0.012	Putative_Peptide_chain_release_factor_2	AT1G56350
TC04G012530	-0.30	-1.23	0.00	0.003	NA	NA
TC09G027220	-0.30	-1.23	0.00	0.004	Putative_uncharacterized_protein	AT1G08480
TC03G022040	-0.30	-1.23	0.00	0.010	Uncharacterized_protein_C16G5.07c	AT4G27585
TC09G034150	-0.30	-1.23	0.00	0.038	Ran-binding_protein_1_homolog_c	AT1G07140
TC05G001880	-0.30	-1.23	0.00	0.036	Putative_Importin_subunit_beta-1	AT5G53480
TC02G000990	-0.30	-1.23	0.00	0.001	ABC_transporter_C_family_member_2	AT2G34660
TC04G019750	-0.30	-1.23	0.00	0.014	Predicted_protein	NA
TC01G016490	-0.30	-1.23	0.00	0.003	60S_ribosomal_protein_L13-1	AT3G49010
TC09G031460	-0.30	-1.23	0.00	0.023	Putative_uncharacterized_protein	NA
TC09G025850	-0.30	-1.23	0.01	0.045	40S_ribosomal_protein_S2-3	AT3G57490
TC07G008920	-0.30	-1.23	0.00	0.030	NA	NA
TC08G008470	-0.30	-1.23	0.00	0.002	Predicted_protein	AT1G20870
TC04G017820	-0.30	-1.23	0.00	0.002	Putative_uncharacterized_protein	AT5G40520
TC01G012930	-0.30	-1.23	0.00	0.020	Predicted_protein	NA
TC05G004950	-0.30	-1.24	0.00	0.000	Putative_Retinol_dehydrogenase_12	AT2G37540
TC04G013830	-0.30	-1.24	0.00	0.000	Putative_lipid_phosphate_phosphatase_3,_chloroplastic	AT3G02600
TC04G023310	-0.31	-1.24	0.00	0.001	40S_ribosomal_protein_S16	AT2G09990
TC09G010180	-0.31	-1.24	0.00	0.014	Putative_Sigma_factor_sigB_regulation_protein_rsbQ	AT3G24420
TC08G003620	-0.31	-1.24	0.00	0.011	Predicted_protein	AT1G19990
TC06G014840	-0.31	-1.24	0.00	0.011	Nudix_hydrolase_15,_mitochondrial	AT1G28960
TC09G027090	-0.31	-1.24	0.00	0.010	Putative_Lysosomal_beta_glucosidase	AT5G20950
TC03G010150	-0.31	-1.24	0.00	0.008	40S_ribosomal_protein_S9-2	AT5G39850
TC02G031380	-0.31	-1.24	0.00	0.024	Predicted_protein	AT1G26300
TC08G008760	-0.31	-1.24	0.00	0.027	40S_ribosomal_protein_S6	AT5G10360
TC03G014890	-0.31	-1.24	0.01	0.041	Deoxycytidylate_deaminase	AT3G48540
TC04G010210	-0.31	-1.24	0.00	0.024	Probable_UDP-glucose_6-dehydrogenase_1	AT3G29360

TC05G028110	-0.31	-1.24	0.00	0.001	Predicted_protein	AT5G27990
TC09G020550	-0.31	-1.24	0.00	0.004	Fasciclin-like_arabinogalactan_protein_9	AT1G03870
TC07G017060	-0.31	-1.24	0.00	0.024	40S_ribosomal_protein_S19-3	AT5G61170
TC00G086510	-0.31	-1.24	0.00	0.035	NA	NA
TC09G011200	-0.31	-1.24	0.00	0.021	NHP2-like_protein_1	AT5G20160
TC09G021860	-0.31	-1.24	0.00	0.022	Auxin_response_factor_2	AT5G20730
TC00G025590	-0.32	-1.24	0.00	0.021	Putative_RNA-binding_protein_38	NA
TC05G030220	-0.32	-1.25	0.00	0.024	F-actin-capping_protein_subunit_alpha	AT3G05520
TC08G012990	-0.32	-1.25	0.00	0.008	Thioredoxin-like_protein_slr0233	AT1G76760
TC05G019890	-0.32	-1.25	0.00	0.003	Calreticulin-3	AT1G65290
TC01G006940	-0.32	-1.25	0.00	0.004	DEAD-box_ATP-dependent_RNA_helicase_16	AT4G34910
TC00G014640	-0.32	-1.25	0.00	0.013	Pantoate--beta-alanine_ligase	AT5G48840
TC03G010910	-0.32	-1.25	0.00	0.021	NA	NA
TC08G013470	-0.32	-1.25	0.00	0.000	NA	NA
TC03G025070	-0.32	-1.25	0.01	0.044	Putative_Histidine_triad_nucleotide-binding_protein_3	AT4G16566
TC00G037760	-0.32	-1.25	0.00	0.036	NA	NA
TC07G010830	-0.32	-1.25	0.00	0.008	50S_ribosomal_protein_L13,_chloroplastic	AT1G78630
TC02G016150	-0.32	-1.25	0.00	0.011	Prohibitin-2	AT4G28510
TC01G033710	-0.32	-1.25	0.00	0.031	60S_acidic_ribosomal_protein_P1-1	AT5G24510
TC01G028890	-0.33	-1.25	0.00	0.036	Elongation_factor_Tu,_mitochondrial	AT4G02930
TC04G015810	-0.33	-1.25	0.00	0.021	Putative_uncharacterized_protein	AT3G08600
TC01G010870	-0.33	-1.25	0.00	0.005	Predicted_protein	NA
TC01G040350	-0.33	-1.25	0.00	0.006	Notchless_protein_homolog	AT5G52820
TC05G009090	-0.33	-1.25	0.01	0.048	1-(5-phosphoribosyl)-5-[(5-phosphoribosylamino)methylideneamino]_imidazole-4-carboxamide_isomerase,_chloroplastic	AT2G36230
TC04G026210	-0.33	-1.25	0.00	0.000	60S_ribosomal_protein_L23	AT1G04480
TC03G029950	-0.33	-1.25	0.00	0.022	Predicted_protein	NA
TC05G023650	-0.33	-1.26	0.00	0.011	Putative_Probable_LRR_receptor-like_serine/threonine-protein_kinase_At1g14390	AT3G03770
TC09G014740	-0.33	-1.26	0.00	0.020	Hypothetical_protein	NA
TC01G034330	-0.33	-1.26	0.00	0.009	Molybdopterin_biosynthesis_protein_CNX3	AT1G01290
TC01G035210	-0.33	-1.26	0.00	0.014	Reticulon-like_protein_B5	AT2G46170
TC09G011790	-0.33	-1.26	0.01	0.042	Predicted_protein	NA
TC01G009570	-0.34	-1.26	0.00	0.018	Predicted_protein	AT5G10070
TC01G039950	-0.34	-1.26	0.00	0.008	Putative_uncharacterized_protein	AT2G44640
TC07G017420	-0.34	-1.26	0.00	0.010	Putative_uncharacterized_protein	AT3G12685
TC08G003530	-0.34	-1.26	0.00	0.012	NA	NA
TC02G000470	-0.34	-1.26	0.00	0.014	Protein_OSB2,_chloroplastic	AT4G20010
TC01G029100	-0.34	-1.26	0.00	0.013	40S_ribosomal_protein_S7	AT3G02560

TC01G037770	-0.34	-1.26	0.00	0.009	40S_ribosomal_protein_S13	AT4G00100
TC01G017780	-0.34	-1.26	0.00	0.010	Triacylglycerol_lipase_2,_putative	AT1G04985
TC02G012120	-0.34	-1.26	0.00	0.036	LL-diaminopimelate_aminotransferase,_chloroplastic	NA
TC05G030450	-0.34	-1.26	0.00	0.032	60S_ribosomal_protein_L18-2	AT3G05590
TC09G003040	-0.34	-1.27	0.00	0.032	NA	NA
TC01G011080	-0.34	-1.27	0.00	0.018	ATP_synthase_subunit_epsilon,_mitochondrial	AT1G51650
TC01G039910	-0.35	-1.27	0.00	0.033	40S_ribosomal_protein_S11	AT5G23740
TC02G003200	-0.35	-1.27	0.00	0.027	mRNA_turnover_protein_4_homolog	AT1G25260
TC05G027990	-0.35	-1.27	0.00	0.001	40S_ribosomal_protein_S24-2	AT3G04920
TC02G010630	-0.35	-1.27	0.01	0.048	Putative_uncharacterized_protein	AT4G33625
TC01G025870	-0.35	-1.27	0.00	0.011	60S_ribosomal_protein_L23	AT2G33370
TC02G025170	-0.35	-1.27	0.00	0.001	Putative_uncharacterized_protein	AT1G13030
TC00G027040	-0.35	-1.27	0.00	0.029	Probable_methyltransferase_PMT25	AT1G29470
TC05G007060	-0.35	-1.27	0.00	0.020	Predicted_protein	AT2G36930
TC05G003150	-0.35	-1.28	0.00	0.030	60S_ribosomal_protein_L35a-3	AT1G74270
TC06G013850	-0.35	-1.28	0.00	0.011	Predicted_protein	AT4G18820
TC03G019700	-0.35	-1.28	0.00	0.000	Arginine/serine-rich-splicing_factor_RSP40	AT4G25500
TC09G001220	-0.35	-1.28	0.00	0.036	Putative_uncharacterized_protein	AT2G24860
TC02G024050	-0.36	-1.28	0.00	0.028	Ubiquitin	AT2G47110
TC09G014300	-0.36	-1.28	0.00	0.014	Putative_GDP-L-fucose_synthase_2	AT1G17890
TC03G016690	-0.36	-1.28	0.00	0.007	Putative_uncharacterized_protein	AT5G51220
TC05G009030	-0.36	-1.28	0.00	0.039	60S_ribosomal_protein_L10a-1	AT1G08360
TC03G031020	-0.36	-1.28	0.00	0.000	Predicted_protein	AT1G31650
TC01G022240	-0.36	-1.29	0.00	0.002	Catalytic,_putative	AT3G07060
TC07G012650	-0.36	-1.29	0.00	0.036	Interactor_of_constitutive_active_ROPs_1	AT1G78430
TC03G021500	-0.37	-1.29	0.00	0.012	Ubiquitin	AT3G52590
TC10G008390	-0.37	-1.29	0.00	0.001	60S_ribosomal_protein_L24	AT2G36620
TC02G020140	-0.37	-1.29	0.00	0.000	Mitochondrial_import_receptor_subunit_TOM20	AT1G27390
TC05G019600	-0.37	-1.30	0.00	0.027	Probable_nucleolar_GTP-binding_protein_1	NA
TC05G018860	-0.38	-1.30	0.01	0.046	Enoyl-[acyl-carrier-protein]_reductase_[NADH],_chloroplastic	AT2G05990
TC09G025120	-0.38	-1.30	0.00	0.022	Predicted_protein	AT2G26810
TC06G003210	-0.38	-1.30	0.01	0.047	Formamidopyrimidine-DNA_glycosylase,_putative	NA
TC07G004790	-0.38	-1.30	0.00	0.017	Thiazole_biosynthetic_enzyme,_chloroplastic	AT5G54770
TC05G019880	-0.38	-1.30	0.00	0.008	Predicted_protein_(Fragment)	AT2G43945
TC08G009520	-0.38	-1.30	0.00	0.033	Putative_uncharacterized_protein	AT1G22140
TC06G008530	-0.38	-1.30	0.01	0.042	NA	NA
TC06G007970	-0.38	-1.30	0.00	0.013	Putative_Zinc_phosphodiesterase_ELAC_protein_2	NA

TC05G020980	-0.39	-1.31	0.00	0.036	DNA-binding_protein_RHL1	NA
TC04G017880	-0.39	-1.31	0.00	0.022	Putative_Disease_resistance_protein_R GA2	AT5G09320
TC02G002860	-0.39	-1.31	0.01	0.044	Putative_Uncharacterized_protein_C16o rf61_homolog	AT4G21192
TC01G010280	-0.39	-1.31	0.00	0.000	UPF0202_protein_At1g10490	AT3G57940
TC05G028310	-0.39	-1.31	0.00	0.001	Probable_nucleolar_protein_5-1	AT5G27120
TC02G009030	-0.39	-1.31	0.00	0.005	40S_ribosomal_protein_S16	AT2G09990
TC02G009470	-0.40	-1.32	0.00	0.010	Putative_Transcription_factor_MYB44	AT4G37260
TC06G020520	-0.40	-1.32	0.00	0.000	NA	NA
TC04G020880	-0.40	-1.32	0.00	0.018	Probable_ribonuclease_P/MRP_protein_ subunit_POP5	AT1G04635
TC05G011670	-0.40	-1.32	0.00	0.009	Putative_uncharacterized_protein	AT3G11660
TC09G003430	-0.40	-1.32	0.00	0.002	Putative_uncharacterized_protein	NA
TC00G068320	-0.40	-1.32	0.00	0.010	NA	NA
TC06G002570	-0.40	-1.32	0.00	0.010	Putative_Elongation_factor_P	AT4G26310
TC01G037950	-0.40	-1.32	0.00	0.007	Putative_Auxin-induced_protein_X10A	AT3G60690
TC01G002060	-0.40	-1.32	0.00	0.008	Putative_Lactoylglutathione_lyase	AT1G15380
TC08G000470	-0.40	-1.32	0.00	0.033	Putative_Non-specific_lipid-transfer_protein-like	AT5G42890
TC09G019240	-0.40	-1.32	0.00	0.012	Hypothetical_protein	AT5G57120
TC06G016840	-0.40	-1.32	0.00	0.017	Putative_Telomerase_Cajal_body_protei n_1	AT4G21520
TC01G030420	-0.41	-1.33	0.00	0.006	Putative_uncharacterized_protein	AT1G02870
TC09G029690	-0.41	-1.33	0.00	0.033	Putative_Gibberellin_2-beta-dioxygenase_8	AT5G58660
TC10G015880	-0.41	-1.33	0.00	0.014	Annexin_D3	AT2G38760
TC10G006280	-0.41	-1.33	0.00	0.008	Putative_Exosome_complex_exonucleas e_RRP42	AT3G07750
TC03G002170	-0.42	-1.33	0.00	0.022	Putative_Protein_MAK16_homolog_B	AT1G23280
TC06G018680	-0.42	-1.33	0.00	0.036	NA	NA
TC05G021490	-0.42	-1.33	0.01	0.047	Putative_30S_ribosomal_protein_S5	AT1G64880
TC03G018530	-0.42	-1.34	0.00	0.001	40S_ribosomal_protein_S13	AT4G00100
TC03G022370	-0.42	-1.34	0.00	0.001	60S_ribosomal_protein_L9	AT1G33140
TC03G020780	-0.42	-1.34	0.01	0.040	Putative_uncharacterized_protein_DDB_ G0287975	NA
TC04G009760	-0.42	-1.34	0.01	0.040	NA	NA
TC01G034910	-0.42	-1.34	0.00	0.003	Exosome_complex_exonuclease_RRP4 1	AT3G61620
TC06G007780	-0.42	-1.34	0.00	0.008	Putative_uncharacterized_protein	AT3G16310
TC01G033090	-0.42	-1.34	0.00	0.038	Predicted_protein	AT2G46930
TC08G003330	-0.42	-1.34	0.00	0.006	Predicted_protein	AT5G39600
TC03G018440	-0.42	-1.34	0.00	0.006	40S_ribosomal_protein_S20-2	AT3G47370
TC03G030020	-0.42	-1.34	0.00	0.000	Probable_xyloglucan_endotransglucosyl ase/hydrolase_protein_30	AT4G18990
TC00G054320	-0.43	-1.35	0.00	0.010	Predicted_protein	AT2G27470
TC03G002320	-0.43	-1.35	0.00	0.001	Predicted_protein	AT3G18580
TC01G016320	-0.43	-1.35	0.00	0.012	Pyrophosphate--fructose_6-phosphate_1-	AT3G08000

					phosphotransferase_subunit_beta	
TC10G001900	-0.44	-1.35	0.00	0.031	Auxin_transporter-like_protein_2	NA
TC05G020220	-0.45	-1.36	0.00	0.008	40S_ribosomal_protein_S28	AT5G03850
TC05G028430	-0.45	-1.36	0.00	0.032	Short-chain_type_dehydrogenase/reductase	AT4G13180
TC09G015240	-0.45	-1.37	0.00	0.001	UDP-glucuronate_4-epimerase_1	AT4G30440
TC05G020310	-0.45	-1.37	0.00	0.000	Putative_Protein_RRP5_homolog	AT3G11964
TC10G001180	-0.45	-1.37	0.00	0.004	Putative_Pentatricopeptide_repeat-containing_protein_At3g56030	AT2G40240
TC02G007060	-0.45	-1.37	0.00	0.022	NA	NA
TC05G005730	-0.46	-1.37	0.00	0.014	Probable_adenylate_kinase_1,_chloroplastic	AT2G37250
TC04G027980	-0.46	-1.38	0.00	0.006	60S_ribosome_subunit_biogenesis_protein_NIP7_homolog	AT4G15770
TC03G013270	-0.46	-1.38	0.00	0.001	H/ACA_ribonucleoprotein_complex_subunit_2-like_protein	AT5G08180
TC04G012070	-0.46	-1.38	0.00	0.005	DNA-directed_RNA_polymerase_2,_chloroplastic/mitochondrial	AT5G15700
TC04G002710	-0.46	-1.38	0.00	0.004	Prohibitin-1,_mitochondrial	AT3G27280
TC03G013880	-0.46	-1.38	0.00	0.016	Putative_Predicted_protein	NA
TC08G000320	-0.47	-1.38	0.00	0.025	Predicted_protein	AT3G49180
TC03G016330	-0.47	-1.39	0.00	0.008	NA	NA
TC04G005100	-0.47	-1.39	0.00	0.008	Aspartokinase_2,_chloroplastic	AT5G13280
TC02G028660	-0.47	-1.39	0.00	0.015	MLP-like_protein_28	AT1G70830
TC07G008960	-0.48	-1.39	0.00	0.037	Transcription_elongation_factor_1_homolog	AT5G46030
TC06G016960	-0.48	-1.39	0.00	0.037	Putative_Dynein_light_chain_LC6,_flagellar_outer_arm	AT4G27360
TC01G012470	-0.48	-1.40	0.00	0.006	NA	NA
TC01G005520	-0.49	-1.40	0.00	0.037	NA	NA
TC03G012450	-0.49	-1.40	0.00	0.007	Protein_COBRA	AT5G60920
TC09G010980	-0.49	-1.40	0.00	0.006	NA	NA
TC03G025500	-0.49	-1.40	0.00	0.024	NA	NA
TC08G011180	-0.49	-1.41	0.01	0.050	NA	NA
TC09G003710	-0.50	-1.41	0.00	0.005	Uncharacterized_protein_At1g55205.2	AT1G55205
TC09G006410	-0.50	-1.42	0.00	0.033	Putative_uncharacterized_protein	AT1G19240
TC06G014600	-0.51	-1.42	0.00	0.013	Predicted_protein	AT1G29050
TC09G005750	-0.52	-1.43	0.00	0.012	Putative_Chalcone--flavonone_isomerase_2-B	NA
TC00G051081	-0.52	-1.43	0.00	0.005	NA	NA
TC00G030840	-0.52	-1.43	0.01	0.047	Predicted_protein	AT2G20515
TC10G003410	-0.52	-1.43	0.00	0.003	Putative_uncharacterized_protein	AT3G04020
TC01G034580	-0.52	-1.43	0.00	0.001	DEAD-box_ATP-dependent_RNA_helicase_26	AT5G63630
TC09G031410	-0.52	-1.44	0.00	0.005	Predicted_protein	AT5G59500
TC03G023410	-0.52	-1.44	0.00	0.007	NA	NA
TC03G020580	-0.53	-1.44	0.00	0.000	Hypothetical_protein	AT5G23760

TC00G013030	-0.53	-1.44	0.00	0.008	Putative_uncharacterized_protein	NA
TC10G001540	-0.53	-1.44	0.00	0.006	Peptidyl-prolyl_cis-trans_isomerase_CYP19-4	AT3G55920
TC00G026710	-0.53	-1.44	0.00	0.002	Pentatricopeptide_repeat-containing_protein_At1g80270,_mitochondrial	AT1G80270
TC00G020080	-0.53	-1.44	0.00	0.035	Putative_Sulfotransferase_16	AT5G07010
TC10G011460	-0.53	-1.45	0.00	0.018	Cytochrome_P450_77A3	AT3G10570
TC04G005410	-0.53	-1.45	0.00	0.011	40S_ribosomal_protein_S27-2	AT3G61110
TC10G016810	-0.54	-1.45	0.00	0.022	Predicted_protein	AT2G40820
TC06G009980	-0.54	-1.45	0.00	0.013	Putative_Midasin	AT1G67120
TC01G000260	-0.54	-1.46	0.01	0.041	NA	NA
TC10G012980	-0.54	-1.46	0.00	0.018	Putative_uncharacterized_protein	AT3G12390
TC09G012290	-0.54	-1.46	0.00	0.000	Arogenate_dehydrogenase_1,_chloroplastic	AT1G15710
TC00G026330	-0.55	-1.46	0.00	0.035	Putative_uncharacterized_protein	NA
TC02G033190	-0.55	-1.46	0.00	0.001	Transcription_factor,_putative	AT1G69580
TC05G013900	-0.55	-1.47	0.00	0.009	G2/mitotic-specific_cyclin_S13-6	AT5G06150
TC05G005490	-0.55	-1.47	0.00	0.017	NA	NA
TC00G015920	-0.56	-1.47	0.00	0.006	DNA_gyrase_subunit_A,_chloroplastic/mitochondrial	AT3G10690
TC05G016800	-0.57	-1.48	0.00	0.012	Putative_uncharacterized_protein	NA
TC01G025640	-0.57	-1.48	0.00	0.029	Rho_GDP-dissociation_inhibitor_1	AT3G07880
TC02G013230	-0.57	-1.49	0.00	0.006	Mitochondrial_import_receptor_subunit_TOM40_homolog_1	AT3G20000
TC04G020530	-0.58	-1.50	0.00	0.000	Glycine-rich_RNA-binding_protein_2,_mitochondrial	AT4G13850
TC05G022200	-0.58	-1.50	0.00	0.001	Putative_Uncharacterized_protein_C3B8.09	AT2G43650
TC01G019970	-0.58	-1.50	0.01	0.040	Putative_uncharacterized_protein	AT5G48500
TC09G030630	-0.59	-1.50	0.00	0.016	Mitochondrial_import_inner_membrane_translocase_subunit_Tim10	AT2G29530
TC02G006970	-0.59	-1.50	0.00	0.000	Hypothetical_protein	AT2G21660
TC01G034940	-0.59	-1.50	0.00	0.027	GDSL_esterase/lipase_At4g01130	AT4G01130
TC09G006140	-0.59	-1.51	0.01	0.043	Pectinesterase/pectinesterase_inhibitor_PPE8B	AT4G33220
TC09G023330	-0.60	-1.51	0.00	0.035	Putative_copper-transporting_ATPase_3	AT5G44790
TC03G014200	-0.60	-1.52	0.00	0.015	Putative_Oxygen-independent_coproporphyrinogen-III_oxidase-like_protein_sll1917	AT5G63290
TC04G015700	-0.62	-1.54	0.00	0.010	NA	NA
TC00G012140	-0.62	-1.54	0.00	0.018	NA	NA
TC00G087940	-0.62	-1.54	0.00	0.021	ATP-dependent_RNA_and_DNA_helicase,_putative	NA
TC10G014000	-0.63	-1.54	0.00	0.000	Probable_protein_arginine_N-methyltransferase_3	AT3G12270
TC03G026830	-0.64	-1.56	0.00	0.036	Putative_uncharacterized_protein	AT4G23060
TC06G017480	-0.65	-1.57	0.00	0.001	Putative_Pentatricopeptide_repeat-containing_protein_At3g13150	AT1G55890
TC03G028600	-0.65	-1.57	0.00	0.031	NA	NA
TC06G021200	-0.67	-1.59	0.00	0.026	NA	NA

TC04G005150	-0.68	-1.60	0.00	0.005	NA	NA
TC04G022740	-0.68	-1.60	0.00	0.007	Ribonucleoside-diphosphate_reductase_small_chain_A	AT3G23580
TC00G087770	-0.68	-1.61	0.00	0.038	Probable_peptide_transporter_At1g52190	NA
TC03G029820	-0.69	-1.61	0.00	0.001	50S_ribosomal_protein	AT4G23620
TC08G007520	-0.70	-1.62	0.00	0.004	Outer_plastidial_membrane_protein_porin	AT5G67500
TC06G011110	-0.71	-1.64	0.00	0.006	60S_ribosomal_protein_L44	AT4G14320
TC04G030320	-0.72	-1.65	0.00	0.036	Putative_Dehydrogenase/reductase_SDR_family_member_on_chromosome_X	AT5G15940
TC06G016980	-0.73	-1.65	0.00	0.002	Sugar_carrier_protein_C	AT1G11260
TC03G025560	-0.73	-1.66	0.00	0.004	Putative_UPF0551_protein_C8orf38_homolog,_mitochondrial	AT1G62730
TC09G026710	-0.73	-1.66	0.01	0.045	Putative_Probable_peptide_transporter_At1g52190	AT3G45680
TC07G016420	-0.74	-1.66	0.00	0.018	Hypothetical_protein	NA
TC02G033730	-0.77	-1.70	0.00	0.012	Origin_recognition_complex_subunit_6	AT1G26840
TC07G002090	-0.77	-1.71	0.00	0.006	NA	NA
TC08G014320	-0.78	-1.71	0.00	0.024	Gibberellin-regulated_protein_2,_putative	AT1G75750
TC01G006720	-0.78	-1.71	0.00	0.004	NA	NA
TC04G001330	-0.82	-1.76	0.00	0.005	NA	NA
TC03G027850	-0.83	-1.78	0.00	0.002	NA	NA
TC05G002920	-0.84	-1.79	0.01	0.040	Peptide_transporter_PTR1	AT3G54140
TC05G028950	-0.87	-1.82	0.00	0.037	NA	NA
TC09G034060	-0.92	-1.89	0.00	0.035	NA	NA
TC01G006680	-0.93	-1.91	0.00	0.020	NA	NA
TC10G001550	-1.03	-2.04	0.00	0.000	Putative_uncharacterized_protein	AT2G40080

[a] Mean differences and p values were calculated by fitting a general linear model in which the SA to H_2O differences (within leaf type) were modeled against *overall_mean* + *leaf type + error,* then testing if *overall_mean* was significantly different than zero. Fold change columns show transformed and untransformed values of *overall_mean.* A BH multiple testing adjustment was used for determination of significance.

Supplemental Table 2 - Genes with statistically significant differential regulation in genotype ICS1

TC ID	Log_2 Fold Change ($SA-H_2O$)[a]	Actual Fold Change ($SA-H_2O$)[a]	p-value[a]	BH-corrected p-value[a]	Arabidopsis Annotation	AThal BestHitID
TC04G029300	2.70	6.49	0.00	0.001	Glucan_endo-1,3-beta-glucosidase,_basic_vacuolar_isoform	AT3G57270
TC05G009360	2.33	5.03	0.00	0.028	NA	NA
TC08G006890	2.24	4.72	0.00	0.010	Branched-chain-amino-acid_aminotransferase_2,_chloroplastic	AT1G10070
TC10G012390	2.24	4.72	0.00	0.011	Hypothetical_protein	NA
TC02G003890	2.18	4.54	0.00	0.012	Endochitinase_1	AT3G12500
TC09G034930	2.15	4.45	0.00	0.015	Peroxidase_4	AT5G05340
TC03G003050	2.09	4.25	0.00	0.020	Predicted_protein	AT1G68320
TC04G016440	2.04	4.11	0.00	0.005	NA	NA
TC03G021520	2.02	4.06	0.00	0.016	Homeobox_protein,_putative	NA
TC05G025040	2.02	4.04	0.00	0.025	NAC_domain-containing_protein_29	AT3G04070
TC04G027520	1.99	3.99	0.00	0.026	UPF0497_membrane_protein_12	AT4G15610
TC09G029650	1.90	3.73	0.01	0.050	Asparagine_synthetase_[glutamine-hydrolyzing]	AT3G47340
TC01G019270	1.77	3.41	0.00	0.000	Putative_uncharacterized_protein	AT2G04240
TC05G003590	1.70	3.24	0.00	0.005	Putative_uncharacterized_protein	AT5G02020
TC09G012750	1.62	3.07	0.00	0.006	NA	NA
TC03G016070	1.60	3.03	0.00	0.028	NA	NA
TC02G011270	1.57	2.98	0.00	0.002	ABC_transporter_G_family_member_11	AT1G17840
TC03G022890	1.55	2.93	0.00	0.001	Putative_Methionine_gamma-lyase	AT1G64660
TC01G035050	1.50	2.83	0.00	0.003	Acidic_endochitinase	AT5G24090
TC08G011870	1.50	2.82	0.00	0.000	2-oxoisovalerate_dehydrogenase_subunit_alpha,_mitochondrial	AT1G21400
TC01G000770	1.44	2.72	0.01	0.040	Endochitinase	AT3G12500
TC03G026900	1.44	2.71	0.00	0.006	Heat_stress_transcription_factor_B-2b	NA
TC06G001520	1.43	2.69	0.00	0.016	NA	NA
TC07G009500	1.43	2.69	0.00	0.020	Epidermis-specific_secreted_glycoprotein_EP1	AT1G78850
TC05G003840	1.37	2.59	0.00	0.000	Delta-1-pyrroline-5-carboxylate_synthase	AT2G39800
TC03G017810	1.36	2.57	0.00	0.005	Putative_DNA-damage-repair/toleration_protein_DRT100	AT1G33590
TC09G015270	1.34	2.53	0.00	0.014	NA	NA
TC00G016740	1.31	2.47	0.00	0.009	GHMYB10	NA

TC09G008760	1.30	2.46	0.00	0.009	Putative_Probable_glutamate_carboxypeptidase_2	AT5G19740
TC03G025490	1.30	2.46	0.00	0.005	Acid_beta-fructofuranosidase	AT1G12240
TC09G024830	1.27	2.41	0.00	0.013	Putative_Epoxide_hydrolase_2	AT3G05600
TC03G014450	1.27	2.41	0.00	0.036	Predicted_protein	AT5G63350
TC03G016040	1.23	2.35	0.00	0.000	Predicted_protein	AT5G61820
TC09G010220	1.19	2.29	0.01	0.043	Putative_DVL	AT4G13395
TC04G004670	1.18	2.26	0.00	0.020	Transcription_factor,_putative	AT3G15510
TC09G009610	1.18	2.26	0.00	0.011	Dynein_light_chain_1,_cytoplasmic	AT3G16120
TC06G020410	1.16	2.23	0.00	0.009	Cystatin-like_protein	AT5G47550
TC01G029220	1.14	2.21	0.00	0.000	Probable_gibberellin_receptor_GID1L2	AT3G63010
TC09G024130	1.11	2.15	0.00	0.000	Lichenase	AT4G16260
TC01G005850	1.10	2.15	0.00	0.019	Predicted_protein	AT4G37300
TC02G002100	1.10	2.14	0.00	0.001	COR413-PM2,_putative	AT2G15970
TC00G089300	1.08	2.11	0.00	0.005	Heat_shock_protein_101	AT1G74310
TC09G023030	1.06	2.09	0.00	0.029	DNA-damage-repair/toleration_protein_DRT100	AT3G20820
TC09G033260	1.06	2.09	0.00	0.007	Predicted_protein_(Fragment)	AT3G43790
TC02G032090	1.06	2.08	0.00	0.025	Ureide_permease_2	AT2G03530
TC06G015310	1.05	2.07	0.00	0.016	Putative_DnaJ_homolog_subfamily_B_member_4	AT2G20560
TC03G030500	1.04	2.06	0.00	0.000	Putative_uncharacterized_protein	AT1G72030
TC06G001650	1.03	2.05	0.00	0.014	Predicted_protein	AT1G52565
TC04G028750	1.03	2.05	0.00	0.003	Putative_Major_allergen_Pru_av_1	NA
TC03G027270	1.01	2.01	0.00	0.005	Putative_peroxisomal-coenzyme_A_synthetase	AT3G48990
TC05G006380	1.00	2.01	0.00	0.000	Aquaporin_PIP2-2	AT2G37170
TC05G002060	1.00	2.00	0.00	0.005	Pyruvate_decarboxylase_isozyme_2	AT4G33070
TC00G014360	1.00	1.99	0.01	0.044	Cytokinin-N-glucosyltransferase_1	AT3G11340
TC03G019840	0.98	1.98	0.00	0.034	Putative_uncharacterized_protein	AT1G01490
TC03G025670	0.97	1.95	0.00	0.019	21_kDa_protein	AT4G12390
TC04G028730	0.96	1.94	0.00	0.005	Putative_uncharacterized_protein	NA
TC01G035600	0.96	1.94	0.00	0.013	Predicted_protein	AT4G23490
TC03G004830	0.95	1.93	0.00	0.008	Beta-glucosidase_44	AT3G18070
TC08G012700	0.95	1.93	0.00	0.018	Peroxidase_43	AT1G77100
TC09G035070	0.95	1.93	0.00	0.000	Probable_serine/threonine-protein_kinase_WNK4	AT3G51630
TC05G028550	0.93	1.90	0.00	0.017	Glutamate_dehydrogenase_1	AT5G18170
TC03G006290	0.92	1.90	0.00	0.000	Predicted_protein	AT5G17300
TC04G016080	0.91	1.88	0.00	0.027	Serine/threonine-protein_kinase_AtPK19	NA
TC10G016440	0.90	1.87	0.00	0.014	NA	NA
TC00G022640	0.89	1.85	0.00	0.009	UPF0131_protein_At3g02910	AT3G02910
TC01G030720	0.88	1.85	0.00	0.000	Indole-3-acetic_acid-	AT4G02380

					induced_protein_ARG2	
TC01G000680	0.88	1.84	0.00	0.000	Putative_Adipocyte_plasma_membrane-associated_protein	AT3G51430
TC04G026780	0.88	1.84	0.00	0.013	Beta-amylase_1,_chloroplastic	AT3G23920
TC09G012660	0.86	1.82	0.00	0.028	NA	NA
TC02G010120	0.86	1.82	0.01	0.050	Putative_uncharacterized_protein	AT1G27730
TC08G013300	0.86	1.81	0.00	0.009	Peroxidase_12	AT1G71695
TC01G033460	0.86	1.81	0.00	0.009	Hypothetical_protein	AT1G33060
TC04G020200	0.86	1.81	0.00	0.017	Brassinosteroid-regulated_protein_BRU1	AT4G14130
TC00G045560	0.85	1.81	0.00	0.018	Alanine--glyoxylate_aminotransferase_2_homolog_3,_mitochondrial	AT2G38400
TC09G001650	0.84	1.79	0.00	0.010	Putative_Zinc_finger_protein_3	AT5G10970
TC03G003610	0.84	1.79	0.00	0.000	F-box_protein_At2g32560	AT2G26850
TC03G013700	0.84	1.79	0.00	0.029	Cytochrome_P450_71A25	AT3G48280
TC08G012130	0.84	1.78	0.00	0.033	Predicted_protein	AT4G19950
TC04G002800	0.83	1.78	0.01	0.045	NA	NA
TC03G024660	0.83	1.78	0.00	0.023	Putative_1-aminocyclopropane-1-carboxylate_oxidase	AT4G16770
TC09G033430	0.82	1.77	0.01	0.038	Predicted_protein	AT5G60680
TC01G004380	0.82	1.77	0.00	0.030	Polyneuridine-aldehyde_esterase	AT2G23620
TC08G009250	0.82	1.76	0.00	0.003	Predicted_protein	NA
TC05G026300	0.81	1.76	0.00	0.012	Hypothetical_protein	AT1G08630
TC09G026340	0.81	1.75	0.00	0.000	Isovaleryl-CoA_dehydrogenase_1,_mitochondrial	AT3G45300
TC03G019930	0.81	1.75	0.00	0.001	Beta-carotene_hydroxylase	AT5G52570
TC09G011650	0.80	1.74	0.01	0.043	Putative_Blue_copper_protein	NA
TC05G006130	0.80	1.74	0.00	0.030	Putative_uncharacterized_protein	AT2G42760
TC09G026080	0.80	1.74	0.00	0.000	Aluminum-activated_citrate_transporter	AT1G51340
TC04G020270	0.79	1.73	0.00	0.028	Indole-3-acetate_beta-glucosyltransferase_2	AT1G05560
TC07G006560	0.79	1.73	0.00	0.021	Putative_Stem-specific_protein_TSJT1	AT4G27450
TC02G010600	0.78	1.72	0.01	0.039	Putative_Uncharacterized_isochorismatase_family_protein_pncA	AT2G22570
TC02G008840	0.77	1.71	0.00	0.002	ABSCISIC_ACID-INSENSITIVE_5-like_protein_6	NA
TC06G012620	0.77	1.71	0.00	0.030	Putative_Calcyclin-binding_protein	AT1G30070
TC08G008850	0.77	1.70	0.00	0.000	Cytokinin-O-glucosyltransferase_2	AT1G22360
TC08G000980	0.77	1.70	0.00	0.001	Serine_carboxypeptidase_II-3	AT2G24010
TC01G003940	0.77	1.70	0.00	0.023	STS14_protein,_putative	AT5G66590
TC10G003040	0.75	1.68	0.00	0.000	Putative_Probable_E3_ubiquitin-protein_ligase_HERC2	AT3G55580
TC09G027430	0.75	1.68	0.01	0.049	DnaJ_protein_homolog	AT3G44110
TC00G024440	0.74	1.67	0.00	0.017	NA	NA
TC00G033180	0.74	1.67	0.00	0.000	Putative_Predicted_protein	NA

TC01G016700	0.74	1.67	0.00	0.016	Hypothetical_protein	NA
TC05G015130	0.74	1.66	0.00	0.027	N-acylneuraminate-9-phosphatase,_putative	AT2G41250
TC08G007840	0.74	1.66	0.00	0.002	Cytochrome_P450_81D1	AT1G66540
TC09G003640	0.73	1.66	0.00	0.017	Aspartate_aminotransferase,_chloroplastic	AT5G11520
TC03G025660	0.73	1.66	0.00	0.018	Putative_21_kDa_protein	AT5G62360
TC00G044630	0.73	1.66	0.00	0.037	Cytochrome_P450_71D10	AT2G45550
TC00G015660	0.73	1.66	0.00	0.002	NA	NA
TC07G007460	0.73	1.65	0.00	0.007	Putative_serine/threonine-protein_kinase_receptor	AT4G03230
TC00G017110	0.72	1.65	0.00	0.004	Hypothetical_protein	AT4G03420
TC00G013500	0.72	1.65	0.00	0.003	Predicted_protein	AT1G72510
TC08G004140	0.72	1.65	0.00	0.009	Putative_Nucleoredoxin	AT1G60420
TC07G007090	0.71	1.63	0.00	0.024	NA	NA
TC09G008500	0.70	1.63	0.00	0.005	Zinc_finger_CCCH_domain-containing_protein_49	AT2G19810
TC10G001870	0.70	1.62	0.00	0.000	Putative_uncharacterized_protein	AT2G40000
TC03G013020	0.69	1.62	0.01	0.040	Putative_Disease_resistance_response_protein_206	NA
TC02G014240	0.69	1.61	0.00	0.010	Predicted_protein	AT3G21890
TC07G007410	0.69	1.61	0.00	0.001	Transcription_factor,_putative	AT5G54470
TC03G022970	0.68	1.60	0.00	0.009	Metal-nicotianamine_transporter_YSL1	AT5G53550
TC00G035350	0.67	1.59	0.00	0.020	Predicted_protein	NA
TC02G000010	0.66	1.58	0.00	0.025	CTP_synthase	AT3G12670
TC08G007780	0.66	1.58	0.00	0.032	NA	NA
TC00G092080	0.65	1.57	0.00	0.028	Putative_uncharacterized_protein	NA
TC02G025720	0.65	1.57	0.01	0.039	NA	NA
TC07G009970	0.65	1.57	0.00	0.003	Hypothetical_protein	NA
TC09G012140	0.64	1.56	0.00	0.015	Bifunctional_purple_acid_phosphatase_26	AT5G34850
TC00G035500	0.64	1.55	0.00	0.005	Capsanthin/capsorubin_synthase,_chromoplast	AT3G10230
TC03G022640	0.63	1.55	0.00	0.029	NA	NA
TC01G020510	0.63	1.54	0.00	0.000	Uncharacterized_UDP-glucosyltransferase_At1g05670	AT1G05680
TC06G015470	0.62	1.54	0.00	0.000	Ethylene-responsive_transcription_factor_12	AT1G28360
TC03G028600	0.62	1.54	0.01	0.039	NA	NA
TC01G035370	0.62	1.53	0.00	0.020	Putative_uncharacterized_protein	AT1G01490
TC04G024360	0.62	1.53	0.00	0.007	Early_light-induced_protein,_chloroplastic	AT4G14690
TC00G052720	0.61	1.53	0.00	0.013	Pathogen-related_protein	AT1G78780
TC05G014480	0.61	1.53	0.00	0.034	Hypothetical_protein	NA
TC05G002270	0.61	1.52	0.00	0.001	Acyl-coenzyme_A_oxidase_4,_peroxisomal	NA
TC07G002090	0.60	1.52	0.00	0.037	NA	NA

TC08G005980	0.60	1.52	0.00	0.016	Putative_uncharacterized_protein	AT5G65480
TC06G017320	0.60	1.52	0.00	0.010	Putative_serine/threonine-protein_kinase_receptor	AT1G65800
TC06G013590	0.60	1.51	0.00	0.010	Cyclic_phosphodiesterase	AT4G18930
TC01G031060	0.59	1.51	0.00	0.000	Ethylene_receptor_1	AT2G40940
TC04G015230	0.59	1.50	0.00	0.017	NA	NA
TC01G030430	0.59	1.50	0.00	0.031	E3_ubiquitin-protein_ligase_BAH1	AT1G02860
TC02G004940	0.58	1.50	0.00	0.009	Putative_uncharacterized_protein	AT3G12920
TC00G055300	0.58	1.50	0.00	0.034	Tyrosine-sulfated_glycopeptide_receptor_1	AT1G72300
TC01G030310	0.58	1.50	0.01	0.038	Stress-related_protein	AT2G47780
TC01G030440	0.58	1.49	0.00	0.010	Putative_Universal_stress_protein_A-like_protein	AT2G47710
TC04G015180	0.58	1.49	0.00	0.000	ERD15	AT2G41430
TC01G015210	0.57	1.49	0.00	0.020	4-hydroxyphenylpyruvate_dioxygenase	AT1G06570
TC00G036580	0.57	1.49	0.00	0.019	Prolyl_4-hydroxylase_alpha_subunit,_putative	AT3G28480
TC00G001530	0.57	1.48	0.00	0.003	Putative_Peptide_transporter_PTR1	AT1G69870
TC02G028120	0.57	1.48	0.00	0.031	Uncharacterized_protein_At1g14870	AT1G14870
TC05G001360	0.56	1.47	0.00	0.031	Probable_cytochrome_c_biosynthesis_protein	ATMG00830
TC10G006460	0.55	1.47	0.00	0.005	Hypothetical_protein	NA
TC08G015910	0.55	1.46	0.00	0.000	Sucrose_transport_protein_SUC2	AT1G22710
TC01G013181	0.54	1.46	0.00	0.026	NA	NA
TC09G031960	0.54	1.46	0.00	0.020	Heat_shock_cognate_70_kDa_protein	AT3G12580
TC09G030440	0.54	1.45	0.00	0.006	Predicted_protein	AT1G07350
TC06G007990	0.54	1.45	0.01	0.046	Aquaporin_TIP2-1	AT3G16240
TC02G015760	0.54	1.45	0.00	0.001	Putative_LanC-like_protein_2	AT2G20770
TC04G028610	0.53	1.44	0.00	0.019	Putative_Ethylene-responsive_transcription_factor_RAP2-3	AT3G16770
TC01G028670	0.53	1.44	0.00	0.028	Putative_Uncharacterized_isomerase_BH0283	AT1G03210
TC03G022200	0.53	1.44	0.00	0.001	Probable_aquaporin_NIP5-1	AT4G10380
TC09G007600	0.53	1.44	0.00	0.006	Putative_Diacylglycerol_O-acyltransferase_1	NA
TC03G021310	0.53	1.44	0.01	0.050	Protein_yippee-like_At4g27740	AT4G27740
TC05G003410	0.53	1.44	0.00	0.003	Putative_Cytokinin-O-glucosyltransferase_2	AT5G05860
TC08G006000	0.52	1.44	0.00	0.001	NADPH--cytochrome_P450_reductase	AT4G24520
TC04G026120	0.52	1.43	0.00	0.002	Putative_uncharacterized_protein	AT5G13890
TC04G015650	0.52	1.43	0.00	0.025	S-adenosylmethionine_decarboxylase_proenzyme	AT3G25570
TC01G022340	0.52	1.43	0.00	0.000	Putative_PPPDE_peptidase_domain-containing_protein_2	AT3G07090
TC04G023840	0.51	1.43	0.00	0.000	Putative_Lactoylglutathione_lyas	AT2G32090

					e	
TC02G011450	0.51	1.43	0.00	0.024	Riboflavin_biosynthesis_protein_ribBA,_chloroplastic	AT5G64300
TC08G000200	0.51	1.43	0.00	0.016	Putative_uncharacterized_protein	AT4G36930
TC04G000850	0.51	1.43	0.00	0.028	Formate_dehydrogenase,_mitochondrial	AT5G14780
TC01G037720	0.51	1.42	0.00	0.000	Predicted_protein	AT2G45380
TC08G007820	0.51	1.42	0.00	0.029	Putative_Isoflavone_2'-hydroxylase	AT4G37360
TC02G020210	0.50	1.42	0.00	0.000	REF/SRPP-like_protein_At1g67360	AT1G67360
TC01G001220	0.50	1.41	0.00	0.035	Aquaporin_PIP2-7	AT4G35100
TC01G014990	0.50	1.41	0.00	0.031	Putative_Transcription_factor_PIF4	AT2G20180
TC06G009210	0.50	1.41	0.00	0.017	Aspartic_proteinase_nepenthesin-2,_putative	AT5G10770
TC04G022980	0.49	1.41	0.00	0.015	Putative_Basic_7S_globulin	AT1G03230
TC08G008390	0.49	1.40	0.00	0.036	Hypothetical_protein	NA
TC08G001820	0.49	1.40	0.00	0.004	Nucleic_acid_binding_protein,_putative	AT1G03840
TC00G051081	0.49	1.40	0.00	0.007	NA	NA
TC06G010330	0.49	1.40	0.00	0.000	Probable_peroxisomal_(S)-2-hydroxy-acid_oxidase_2	AT3G14130
TC03G019800	0.49	1.40	0.00	0.023	Putative_Uncharacterized_protein_P11E10.01	AT5G52810
TC01G038850	0.48	1.40	0.00	0.022	NA	NA
TC02G001130	0.48	1.40	0.00	0.037	NA	NA
TC03G022190	0.48	1.40	0.01	0.039	NA	NA
TC03G011070	0.48	1.40	0.00	0.016	NA	NA
TC07G003370	0.48	1.39	0.00	0.022	NA	NA
TC05G013900	0.48	1.39	0.00	0.027	G2/mitotic-specific_cyclin_S13-6	AT5G06150
TC08G011990	0.47	1.39	0.00	0.000	F-box/LRR-repeat_protein_5	AT1G21410
TC05G017380	0.47	1.39	0.00	0.010	Putative_calcium-transporting_ATPase_11,_plasma_membrane-type	AT3G57330
TC05G005370	0.47	1.38	0.00	0.000	Ankyrin_repeat-containing_protein_At5g02620	AT1G07710
TC00G061020	0.46	1.38	0.01	0.042	Putative_uncharacterized_protein	NA
TC03G028470	0.46	1.38	0.00	0.017	Arabinogalactan_peptide_20	AT5G24105
TC08G002840	0.46	1.37	0.00	0.028	NA	NA
TC01G036960	0.46	1.37	0.00	0.024	ATPase_GET3	AT1G01910
TC05G020320	0.45	1.37	0.01	0.047	Serine/arginine_rich_splicing_factor,_putative	AT3G13570
TC09G034560	0.45	1.37	0.00	0.014	Putative_Uncharacterized_UDP-glucosyltransferase_At1g05670	AT2G30140
TC00G035780	0.45	1.37	0.00	0.022	Serine/threonine-protein_kinase_CTR1	AT5G03730
TC07G007210	0.45	1.36	0.00	0.009	Cyclic_nucleotide-gated_ion_channel_4	AT5G54250
TC03G018860	0.44	1.36	0.00	0.020	Putative_uncharacterized_protein	AT5G62200
TC00G033120	0.44	1.36	0.00	0.037	Putative_Laccase-14	AT5G03260
TC05G019710	0.44	1.35	0.00	0.003	Phosphatidylcholine-sterol_O-acyltransferase,_putative	AT3G03310

TC10G000750	0.44	1.35	0.00	0.022	Putative_Polyadenylate-binding_protein-interacting_protein_2	AT2G41430
TC09G007190	0.43	1.35	0.00	0.029	Predicted_protein	NA
TC02G015080	0.43	1.35	0.01	0.046	Putative_uncharacterized_protein	AT4G28240
TC03G018200	0.43	1.35	0.01	0.040	Beta-fructofuranosidase,_soluble_isoenzyme_I	AT1G12240
TC00G081420	0.43	1.35	0.01	0.044	Remorin,_putative	AT2G41870
TC09G035000	0.43	1.35	0.00	0.025	Putative_uncharacterized_protein	AT5G58375
TC02G012120	0.43	1.35	0.00	0.013	LL-diaminopimelate_aminotransferase,_chloroplastic	NA
TC04G029200	0.43	1.34	0.00	0.031	GTP-binding_protein_SAR1A	AT3G62560
TC02G010520	0.43	1.34	0.00	0.000	Putative_glutamine_amidotransferase_YLR126C	AT2G23970
TC02G018870	0.42	1.34	0.00	0.009	Uncharacterized_protein_At4g14100	AT4G14100
TC04G025890	0.42	1.34	0.01	0.041	Peroxisomal_membrane_protein_PMP22	AT4G14305
TC05G031750	0.42	1.34	0.00	0.004	Putative_Isopenicillin_N_epimerase	AT5G26600
TC01G003260	0.42	1.34	0.00	0.002	UPF0195_protein_At1g68310	AT3G50845
TC05G000230	0.42	1.34	0.00	0.033	Predicted_protein	AT1G05870
TC09G012860	0.42	1.34	0.00	0.004	Predicted_protein	AT2G12462
TC01G014200	0.42	1.34	0.00	0.016	Citrate_synthase_2,_peroxisomal	AT2G42790
TC02G007500	0.42	1.33	0.00	0.025	Predicted_protein	AT4G34630
TC04G005190	0.42	1.33	0.00	0.000	Putative_Lysosomal_alpha-mannosidase	AT3G26720
TC01G037060	0.41	1.33	0.00	0.014	Putative_Lysophosphatidylcholine_acyltransferase_2	NA
TC01G034930	0.41	1.33	0.00	0.001	Common_plant_regulatory_factor_1	AT2G46270
TC03G015780	0.41	1.33	0.01	0.049	Putative_Lysosomal_alpha-mannosidase	AT3G26720
TC09G029570	0.41	1.33	0.00	0.013	Pheophorbide_a_oxygenase,_chloroplastic	AT3G44880
TC02G027350	0.41	1.33	0.00	0.011	Putative_uncharacterized_protein	NA
TC10G016760	0.40	1.32	0.00	0.007	Hydroxyacylglutathione_hydrolase_cytoplasmic	AT3G10850
TC02G022220	0.40	1.32	0.01	0.043	Putative_Probable_serine/threonine-protein_kinase_At1g18390	NA
TC00G060510	0.40	1.32	0.00	0.030	Predicted_protein	AT1G68440
TC05G027410	0.40	1.32	0.00	0.037	Light-inducible_protein_CPRF2	AT5G28770
TC09G030360	0.40	1.32	0.00	0.001	Peptide_transporter_PTR1	AT3G54140
TC01G027700	0.40	1.32	0.00	0.029	Mavicyanin	NA
TC08G004300	0.40	1.32	0.00	0.025	Putative_uncharacterized_protein	AT5G27280
TC03G023190	0.40	1.32	0.01	0.042	Putative_Predicted_protein	NA
TC06G012100	0.39	1.31	0.00	0.000	Auxin_response_factor_6	AT1G30330
TC01G028230	0.39	1.31	0.00	0.011	Methylcrotonoyl-CoA_carboxylase_subunit_alpha,_mitochondrial	AT1G03090
TC02G028060	0.39	1.31	0.00	0.003	Putative_uncharacterized_protein	NA
TC03G031100	0.39	1.31	0.00	0.009	Putative_Protein_notum_homolo	AT4G19420

					g	
TC00G047240	0.39	1.31	0.00	0.002	Sphingosine-1-phosphate_lyase	AT1G27980
TC00G089680	0.39	1.31	0.01	0.046	Putative_Probable_peptide_trans porter_At1g52190	AT3G47960
TC00G092550	0.38	1.31	0.00	0.012	Protein_PINHEAD	AT5G43810
TC03G020770	0.38	1.30	0.00	0.000	Putative_uncharacterized_protein	AT5G52230
TC05G003500	0.38	1.30	0.00	0.020	Putative_Universal_stress_protei n_A-like_protein	AT3G53990
TC00G038750	0.38	1.30	0.00	0.037	Predicted_protein	AT2G01190
TC01G021990	0.37	1.30	0.00	0.009	Predicted_protein	AT3G25120
TC05G009830	0.37	1.29	0.00	0.024	Putative_Vacuolar_protein_sortin g_55	AT3G11530
TC03G015550	0.37	1.29	0.00	0.002	Putative_uncharacterized_protein	AT3G26890
TC01G002780	0.37	1.29	0.00	0.026	Putative_uncharacterized_protein	AT5G66450
TC02G004320	0.37	1.29	0.00	0.032	Protein_kinase,_putative	NA
TC06G001170	0.37	1.29	0.00	0.031	Putative_Predicted_protein	NA
TC08G004760	0.37	1.29	0.00	0.005	Putative_Protein_TSSC1	NA
TC09G004540	0.36	1.29	0.00	0.020	Putative_UPF0587_protein_C1or f123_homolog	AT4G32930
TC09G033010	0.36	1.29	0.00	0.019	L- ascorbate_peroxidase_2,_cytosol ic	AT1G07890
TC05G004100	0.36	1.29	0.00	0.022	Putative_Lectin- domain_containing_receptor_kin ase_A4.2	AT3G55550
TC01G029910	0.36	1.29	0.00	0.008	Metal_tolerance_protein_C1	NA
TC01G011950	0.36	1.29	0.00	0.005	Predicted_protein	AT4G19950
TC10G002680	0.36	1.28	0.00	0.004	NA	NA
TC08G009800	0.36	1.28	0.00	0.005	Predicted_protein	AT1G36380
TC09G013770	0.36	1.28	0.00	0.002	Polyol_transporter_5	AT3G18830
TC02G028560	0.36	1.28	0.00	0.015	Putative_Probable_ADP- ribosylation_factor_GTPase- activating_protein_AGD11	AT1G70790
TC10G013670	0.35	1.28	0.00	0.011	ABC_transporter_I_family_memb er_17	AT1G67940
TC03G009710	0.35	1.28	0.00	0.012	Predicted_protein	NA
TC03G021650	0.35	1.28	0.00	0.025	RNA-binding_protein,_putative	AT3G49390
TC01G010800	0.35	1.28	0.00	0.005	Putative_uncharacterized_protein	AT3G18800
TC02G004610	0.35	1.28	0.01	0.049	Phytoene_dehydrogenase,_putati ve	NA
TC00G022810	0.35	1.27	0.00	0.000	Histidine_kinase_3_(Fragment)	AT1G27320
TC05G002660	0.35	1.27	0.00	0.033	Putative_Clathrin_light_chain_B	AT2G40060
TC01G021090	0.35	1.27	0.00	0.009	Putative_uncharacterized_protein	AT3G07310
TC03G021030	0.35	1.27	0.00	0.000	PP2A_regulatory_subunit_TAP46	AT5G53000
TC01G006280	0.34	1.27	0.00	0.014	Peroxidase_73	AT4G37530
TC03G029230	0.34	1.27	0.00	0.028	Putative_Probable_protein_phos phatase_2C_55	AT4G16580
TC01G003540	0.34	1.27	0.00	0.000	Probable_protein_phosphatase_ 2C_80	AT4G16580
TC05G015450	0.34	1.27	0.01	0.048	Putative_methyltransferase_DDB _G0268948	AT2G41380

TC01G039930	0.34	1.27	0.01	0.042	NA	NA
TC02G035090	0.34	1.27	0.00	0.002	Predicted_protein	NA
TC09G005201	0.34	1.26	0.00	0.023	NA	NA
TC04G022950	0.34	1.26	0.00	0.025	Dual_specificity_protein_phosphatase,_putative	AT3G23610
TC10G006740	0.34	1.26	0.00	0.008	Putative_Uncharacterized_protein_ycf45	AT3G10420
TC05G025030	0.34	1.26	0.00	0.034	Putative_uncharacterized_protein	AT2G39050
TC01G005060	0.33	1.26	0.00	0.011	Zeaxanthin_epoxidase,_chloroplastic	AT5G67030
TC09G032550	0.33	1.26	0.00	0.006	Calcium-transporting_ATPase_4,_endoplasmic_reticulum-type	AT1G07810
TC05G005870	0.33	1.26	0.00	0.019	E3_ubiquitin-protein_ligase_UPL4	AT5G02880
TC04G014040	0.33	1.26	0.00	0.011	Protease_Do-like_7	NA
TC01G002280	0.33	1.26	0.00	0.015	Glutaredoxin-C3	AT1G77370
TC04G004720	0.33	1.26	0.00	0.018	Putative_uncharacterized_protein	AT3G26890
TC00G014550	0.33	1.26	0.00	0.001	Predicted_protein	AT5G61960
TC03G000810	0.33	1.26	0.00	0.014	Putative_uncharacterized_protein	AT1G24450
TC07G001320	0.33	1.26	0.00	0.009	Putative_uncharacterized_protein	AT5G55640
TC10G007380	0.33	1.26	0.00	0.030	Putative_RING_finger_protein_B	NA
TC01G020630	0.33	1.25	0.00	0.008	Ribonuclease_2	AT2G39780
TC04G020340	0.32	1.25	0.00	0.000	Putative_selenium-binding_protein	AT4G14030
TC02G002980	0.32	1.25	0.00	0.027	Putative_uncharacterized_protein	AT1G25290
TC05G031570	0.32	1.25	0.00	0.028	Putative_Pyrrolidone-carboxylate_peptidase	AT1G56700
TC04G018000	0.32	1.25	0.00	0.009	Predicted_protein	AT3G54200
TC09G002870	0.31	1.24	0.00	0.016	CBL-interacting_serine/threonine-protein_kinase_6	AT4G30960
TC08G002030	0.31	1.24	0.00	0.001	Predicted_protein	AT1G75180
TC01G029870	0.31	1.24	0.00	0.007	Putative_uncharacterized_protein	NA
TC02G010730	0.31	1.24	0.01	0.049	Putative_IgA_FC_receptor	AT5G09530
TC02G029760	0.31	1.24	0.00	0.007	NA	NA
TC08G007220	0.31	1.24	0.01	0.041	70_kDa_peptidyl-prolyl_isomerase	AT3G25230
TC05G003610	0.31	1.24	0.00	0.028	Probable_proteasome_inhibitor	AT3G53970
TC09G014740	0.30	1.23	0.00	0.028	Hypothetical_protein	NA
TC09G032890	0.30	1.23	0.00	0.002	Phototropin-1	AT3G45780
TC09G027400	0.30	1.23	0.00	0.006	NA	NA
TC03G000280	0.30	1.23	0.00	0.032	Lipase,_putative	AT3G48080
TC06G001580	0.29	1.23	0.00	0.019	Putative_uncharacterized_protein	AT5G62200
TC05G021810	0.29	1.23	0.01	0.041	Hypothetical_protein	NA
TC01G018610	0.29	1.23	0.00	0.034	Zinc_finger_CCCH_domain-containing_protein_18	AT2G05160
TC01G020380	0.29	1.22	0.00	0.008	Putative_uncharacterized_protein	AT3G07525
TC00G092230	0.29	1.22	0.00	0.007	Protein_yippee-like_At4g27745	AT4G27745

TC03G010140	0.29	1.22	0.00	0.015	Hypothetical_protein	AT3G47670
TC02G010620	0.29	1.22	0.00	0.005	Beta-ureidopropionase	AT5G64370
TC09G034370	0.29	1.22	0.00	0.016	LRR_repeats_and_ubiquitin-like_domain-containing_protein_At2g30105	AT2G30100
TC02G033920	0.29	1.22	0.00	0.026	Ubiquitin-conjugating_enzyme_E2_2	AT2G02760
TC01G025610	0.28	1.22	0.00	0.035	Predicted_protein	AT5G44060
TC03G019970	0.28	1.22	0.00	0.026	Putative_uncharacterized_protein	AT5G52550
TC08G006660	0.28	1.22	0.00	0.014	Putative_Xylulose_kinase	AT5G49650
TC05G005030	0.28	1.22	0.00	0.027	Putative_uncharacterized_protein	AT3G53670
TC04G025960	0.28	1.22	0.00	0.035	Cyanate_hydratase	AT3G23490
TC09G004620	0.28	1.22	0.01	0.045	Putative_UPF0424_protein_C1orf128	AT2G25950
TC04G004740	0.28	1.21	0.01	0.047	Acyltransferase-like_protein_At3g26840,_chloroplastic	AT5G41120
TC09G034530	0.28	1.21	0.01	0.040	Putative_Cytokinin-O-glucosyltransferase_2	AT2G30150
TC10G006340	0.28	1.21	0.01	0.047	Glycerol-3-phosphate_dehydrogenase_SDP6,_mitochondrial	AT3G10370
TC07G012930	0.28	1.21	0.00	0.007	UPF0497_membrane_protein_f16	AT1G17200
TC01G040610	0.27	1.21	0.00	0.004	Carotenoid_9,10(9',10')-cleavage_dioxygenase_1	AT3G63520
TC03G000760	0.27	1.21	0.00	0.018	Ubiquitin-conjugating_enzyme_E2_5	AT5G41340
TC09G029950	0.27	1.21	0.00	0.005	Putative_NudC_domain-containing_protein_2	AT5G58740
TC08G000900	0.27	1.21	0.00	0.007	Ribonuclease_p/mrp_subunit,_putative	NA
TC05G018110	0.27	1.21	0.00	0.013	Putative_ER-derived_vesicles_protein_ERV14	AT3G12180
TC06G004850	0.27	1.21	0.00	0.029	Putative_Sorcin	NA
TC02G030470	0.27	1.21	0.00	0.027	Triptychon_and_cpc,_putative	AT2G46410
TC01G033620	0.27	1.20	0.00	0.028	Metal_tolerance_protein_A2	AT2G46800
TC09G027220	0.27	1.20	0.00	0.008	Putative_uncharacterized_protein	AT1G08480
TC02G002280	0.27	1.20	0.01	0.042	Probable_E3_ubiquitin-protein_ligase_ARI1	AT4G34370
TC02G008260	0.27	1.20	0.00	0.001	Putative_Protein_SRG1	AT3G19000
TC03G024560	0.27	1.20	0.00	0.027	Putative_Palmitoyl-protein_thioesterase_1	NA
TC02G032660	0.26	1.20	0.00	0.034	Putative_Uncharacterized_protein_MJ0044	AT1G26640
TC10G011790	0.26	1.19	0.00	0.001	DnaJ_protein_homolog	AT3G44110
TC04G017500	0.26	1.19	0.00	0.015	Uncharacterized_protein_At1g18480	NA
TC09G012560	0.25	1.19	0.00	0.024	Homeobox_protein_knotted-1-like_3	AT5G11060
TC01G020700	0.25	1.19	0.01	0.049	Peroxisome_biogenesis_factor_13	NA
TC02G010310	0.25	1.19	0.00	0.011	ADP-ribosylation_factor-like_protein_8A	AT3G49870
TC02G034460	0.25	1.19	0.00	0.032	ATP-dependent_Clp_protease_adapter_protein_clpS	AT1G68660
TC10G005250	0.25	1.19	0.00	0.010	Putative_Transmembrane_protein_115	AT3G07950

TC05G003480	0.25	1.19	0.00	0.002	Peptidyl-prolyl_isomerase_PASTICCINO1	AT3G54010
TC09G001320	0.25	1.19	0.00	0.026	Enhancer_of_rudimentary_homolog	AT5G10810
TC09G034920	0.25	1.19	0.00	0.022	Biogenesis_of_lysosome-related_organelles_complex_1_subunit_1	AT2G30330
TC05G017590	0.25	1.19	0.00	0.025	Probable_calcium-binding_protein_CML35	AT2G41410
TC00G054890	0.24	1.18	0.00	0.009	Leucine_zipper-ef-hand_containing_transmembrane_protein,_putative	AT1G65540
TC03G019280	0.24	1.18	0.00	0.002	L-idonate_5-dehydrogenase	AT5G51970
TC03G025700	0.24	1.18	0.00	0.010	Putative_Hsc70-interacting_protein	AT4G22670
TC03G002410	0.24	1.18	0.01	0.043	Predicted_protein	AT1G73960
TC05G022160	0.24	1.18	0.00	0.000	N-acetyltransferase,_putative	AT5G16800
TC09G014430	0.24	1.18	0.00	0.002	Putative_uncharacterized_protein	AT4G30780
TC07G007080	0.24	1.18	0.00	0.002	NA	NA
TC01G039090	0.24	1.18	0.00	0.018	Putative_Uncharacterized_amino_acid_permease_yfnA	AT1G05940
TC09G013680	0.23	1.18	0.00	0.032	Putative_APO_protein_3,_mitochondrial	AT5G57930
TC03G009790	0.23	1.18	0.00	0.002	Delta-1-pyrroline-5-carboxylate_dehydrogenase_12A1,_mitochondrial	AT5G62530
TC02G033940	0.23	1.17	0.00	0.034	Putative_SNF1-related_protein_kinase_regulatory_subunit_gamma_1	AT1G69800
TC09G011440	0.23	1.17	0.01	0.047	EIN3-binding_F-box_protein_1	AT2G25490
TC04G029430	0.23	1.17	0.00	0.025	Putative_Universal_stress_protein_A-like_protein	AT3G17020
TC02G004260	0.23	1.17	0.00	0.024	F-box_protein_SKIP23	NA
TC04G015860	0.23	1.17	0.00	0.004	Putative_Protein_of_unknown_function_DUF3411	AT3G08640
TC09G000200	0.23	1.17	0.00	0.017	Ubiquitin_carboxyl-terminal_hydrolase_26	NA
TC01G017640	0.23	1.17	0.00	0.015	Ubiquitin-like_protein_SMT3	AT4G26840
TC09G013850	0.23	1.17	0.01	0.047	NEDD8-conjugating_enzyme_Ubc12	AT2G18600
TC04G030390	0.23	1.17	0.00	0.026	BWF1-like_protein	AT5G18525
TC04G027110	0.23	1.17	0.00	0.005	Predicted_protein	AT5G18900
TC06G001660	0.23	1.17	0.00	0.009	Putative_uncharacterized_protein	AT5G25360
TC05G025930	0.23	1.17	0.00	0.020	Predicted_protein	NA
TC08G011390	0.23	1.17	0.01	0.039	Hypothetical_protein	AT4G08330
TC04G030560	0.22	1.17	0.00	0.030	Putative_[Pyruvate_dehydrogenase_[lipoamide]]_kinase,_mitochondrial	AT3G06483
TC03G003260	0.22	1.17	0.01	0.041	NADH_dehydrogenase_[ubiquinone]_iron-sulfur_protein_6	AT1G49140
TC07G009420	0.22	1.17	0.00	0.010	Putative_uncharacterized_protein	AT3G15010
TC00G006190	0.22	1.17	0.01	0.049	Putative_UPF0301_protein_Cag_1601	AT1G33780
TC08G013980	0.22	1.16	0.00	0.022	Putative_uncharacterized_protein	AT4G10080
TC02G034930	0.22	1.16	0.00	0.028	Hypothetical_protein	AT1G26150
TC08G011110	0.22	1.16	0.01	0.043	Aldehyde_dehydrogenase_family_3_member_H1	AT1G44170

TC01G016020	0.22	1.16	0.01	0.047	Putative_Uncharacterized_membrane_protein_yuiD	AT3G21610
TC05G000450	0.22	1.16	0.00	0.029	Putative_ALBINO3-like_protein_2,_chloroplastic	AT3G44370
TC07G001030	0.22	1.16	0.00	0.027	Putative_RPM1-interacting_protein_4	AT5G55850
TC03G015830	0.22	1.16	0.01	0.043	RNA-binding_protein,_putative	AT5G07290
TC04G001950	0.21	1.16	0.00	0.009	Golgin_candidate_6	AT3G27530
TC04G029270	0.21	1.16	0.00	0.025	NA	NA
TC01G037650	0.21	1.16	0.00	0.003	Putative_Dihydroflavonol-4-reductase	AT2G45400
TC00G022710	0.21	1.16	0.01	0.047	NA	NA
TC02G011710	0.20	1.15	0.00	0.002	Serine/arginine_rich_splicing_factor,_putative	AT5G64200
TC01G001110	0.20	1.15	0.00	0.015	RING-H2_finger_protein_ATL4M	AT2G17730
TC03G022260	0.20	1.15	0.00	0.020	Putative_Transmembrane_protein_120_homolog	AT4G10430
TC00G027830	0.20	1.15	0.00	0.003	Putative_5-methylthioadenosine/S-adenosylhomocysteine_deaminase	NA
TC02G012700	0.20	1.15	0.01	0.043	NA	NA
TC08G005000	0.20	1.15	0.01	0.041	Predicted_protein	AT1G20640
TC03G019310	0.20	1.15	0.00	0.003	Predicted_protein	AT1G12580
TC04G015870	0.20	1.15	0.00	0.012	Putative_Protein_gufA	AT3G08650
TC00G069330	0.20	1.15	0.00	0.027	NA	NA
TC01G016290	0.20	1.15	0.00	0.000	Ebs-bah-phd_domain-containing_protein	AT4G22140
TC00G033720	0.20	1.14	0.00	0.017	Putative_Protein_thiJ	AT1G53280
TC08G010320	0.19	1.14	0.00	0.007	Predicted_protein	AT4G08240
TC03G018810	0.19	1.14	0.00	0.017	Putative_Cysteine_and_histidine-rich_domain-containing_protein_1	AT5G51700
TC04G019120	0.19	1.14	0.00	0.029	Probable_serine/threonine-protein_kinase_GCN2	AT3G59410
TC09G013000	0.19	1.14	0.00	0.007	Putative_uncharacterized_protein	AT5G11440
TC08G009890	0.19	1.14	0.00	0.029	Putative_Transmembrane_protein_50A	AT1G36980
TC09G030850	0.19	1.14	0.00	0.030	Putative_Universal_stress_protein_A-like_protein	AT3G53990
TC09G026470	0.18	1.13	0.00	0.010	Predicted_protein_(Fragment)	NA
TC02G024240	0.18	1.13	0.00	0.037	Cysteine-rich_receptor-like_protein_kinase_2	AT1G70520
TC03G017440	0.18	1.13	0.00	0.019	Probable_protein_phosphatase_2C_76	AT5G53140
TC05G005830	0.18	1.13	0.00	0.000	Predicted_protein	NA
TC09G034140	0.18	1.13	0.01	0.040	Clathrin_heavy_chain_1	AT3G08530
TC01G001280	0.18	1.13	0.00	0.024	SKP1-like_protein_1A	AT5G42190
TC05G024520	0.18	1.13	0.00	0.003	Putative_uncharacterized_protein	AT3G05010
TC02G002370	0.18	1.13	0.00	0.017	Hypothetical_protein	NA
TC01G005720	0.18	1.13	0.00	0.020	Probable_cadmium/zinc-transporting_ATPase_HMA1,_chloroplastic	AT4G37270
TC08G014060	0.18	1.13	0.00	0.028	Predicted_protein	AT1G71410

TC04G022210	0.18	1.13	0.00	0.026	Putative_uncharacterized_protein	AT3G57170
TC03G024330	0.18	1.13	0.00	0.008	Protein_BUD31_homolog_2	AT4G21110
TC09G029990	0.18	1.13	0.00	0.018	NA	NA
TC03G027320	0.17	1.13	0.00	0.034	Glutathione_peroxidase_(Fragment)	AT4G11600
TC01G039280	0.17	1.13	0.00	0.019	Putative_uncharacterized_protein	AT1G02120
TC00G017590	0.17	1.12	0.00	0.013	Predicted_protein	AT1G79970
TC04G014440	0.17	1.12	0.00	0.016	Plastidic_glucose_transporter_4	AT5G16150
TC01G040240	0.16	1.12	0.00	0.016	F-box/WD-40_repeat-containing_protein_At3g52030	AT3G52030
TC08G010710	0.16	1.12	0.01	0.043	Putative_Protein_KRTCAP2_homolog	AT1G77350
TC08G011280	0.16	1.12	0.00	0.025	V-type_proton_ATPase_16_kDa_proteolipid_subunit	AT4G34720
TC10G006450	0.15	1.11	0.00	0.027	Predicted_protein	AT2G39170
TC00G059940	0.15	1.11	0.00	0.020	Cytochrome_c1-1,_heme_protein,_mitochondrial	AT5G40810
TC09G010070	0.14	1.10	0.00	0.029	Genomic_DNA,_chromosome_3,_P1_clone:_MOB24	AT3G24490
TC01G007290	0.14	1.10	0.01	0.043	Putative_Protein_spinster_homolog_1	NA
TC00G045230	0.14	1.10	0.01	0.038	Putative_uncharacterized_protein	AT1G77220
TC08G008270	0.14	1.10	0.00	0.014	Putative_bZIP_transcription_factor_60	AT1G42990
TC01G000410	0.14	1.10	0.00	0.005	Predicted_protein	NA
TC09G010390	0.14	1.10	0.01	0.046	Putative_Transmembrane_protein_19	AT5G19930
TC06G019520	0.13	1.10	0.01	0.043	Aspartic_proteinase	AT1G11910
TC10G016790	0.12	1.09	0.01	0.047	Putative_Quinone_oxidoreductase-like_protein_2_homolog	AT3G56460
TC06G006730	-0.11	-1.08	0.00	0.026	Predicted_protein	AT3G15610
TC02G032270	-0.11	-1.08	0.01	0.042	Putative_COBW_domain-containing_protein_1	AT1G26520
TC02G000290	-0.11	-1.08	0.01	0.042	Proteasome_subunit_alpha_type-4	AT3G22110
TC02G007320	-0.11	-1.08	0.01	0.050	Squalene_synthase	AT4G34640
TC02G020400	-0.12	-1.09	0.00	0.035	Putative_uncharacterized_protein	AT3G56820
TC00G080870	-0.12	-1.09	0.00	0.021	Putative_uncharacterized_protein	AT5G01010
TC04G002630	-0.13	-1.10	0.00	0.022	Putative_uncharacterized_protein	AT5G14310
TC06G009360	-0.14	-1.10	0.01	0.042	Serine/threonine_protein_phosphatase_2A_55_kDa_regulatory_subunit_B_beta_isoform	AT1G17720
TC01G040970	-0.14	-1.10	0.00	0.023	Putative_Protein_KIAA0664_homolog	AT3G52140
TC00G019560	-0.14	-1.10	0.01	0.039	Predicted_protein_(Fragment)	AT1G15420
TC01G034650	-0.14	-1.10	0.00	0.018	Uncharacterized_protein_At4g01050	AT4G01050
TC04G030150	-0.14	-1.10	0.00	0.006	Dihydrolipoyl_dehydrogenase_1,_mitochondrial	AT1G48030
TC09G017030	-0.15	-1.11	0.00	0.012	Predicted_protein	AT5G57460
TC10G001890	-0.15	-1.11	0.01	0.044	Syntaxin-31	AT5G05760
TC03G027740	-0.15	-1.11	0.00	0.035	DNA_repair_helicase_XPB1	AT5G41370
TC01G038440	-0.15	-1.11	0.01	0.049	T-	AT3G20050

					complex_protein_1_subunit_alpha	
TC05G018850	-0.15	-1.11	0.00	0.001	Zinc_finger_CCCH_domain-containing_protein_14	AT3G12130
TC00G060130	-0.15	-1.11	0.00	0.035	Putative_Lys-63-specific_deubiquitinase_BRCC36	AT1G80210
TC00G029800	-0.15	-1.11	0.01	0.043	Serine/threonine-protein_phosphatase_PP2A_catalytic_subunit	AT3G58500
TC02G031480	-0.16	-1.11	0.01	0.048	Putative_uncharacterized_protein	AT1G69060
TC00G047370	-0.16	-1.11	0.01	0.047	Putative_GTP-binding_protein_engA	AT5G39960
TC01G028010	-0.16	-1.12	0.00	0.010	WD-repeat_protein,_putative	AT1G03110
TC06G015690	-0.16	-1.12	0.01	0.047	Eukaryotic_translation_initiation_factor_3_subunit_D	AT4G20980
TC09G006690	-0.16	-1.12	0.00	0.034	Lsm1,_putative	AT3G14080
TC01G033350	-0.16	-1.12	0.00	0.013	Translocase_of_chloroplast_33,_chloroplastic	AT1G02280
TC01G010280	-0.16	-1.12	0.00	0.026	UPF0202_protein_At1g10490	AT3G57940
TC09G001360	-0.16	-1.12	0.01	0.049	Probable_protein_phosphatase_2C_60	AT4G31860
TC00G065690	-0.17	-1.12	0.01	0.042	Hypothetical_protein	AT2G02500
TC05G031720	-0.17	-1.12	0.00	0.009	Predicted_protein	AT3G05830
TC01G032990	-0.17	-1.13	0.00	0.024	Protoporphyrinogen_oxidase,_chloroplastic	AT4G01690
TC09G006060	-0.17	-1.13	0.00	0.004	Guanine_nucleotide-exchange-like_protein	AT3G43300
TC09G010660	-0.17	-1.13	0.00	0.018	Serine-threonine_kinase_receptor-associated_protein	AT1G15470
TC07G002300	-0.17	-1.13	0.01	0.049	DNA_topoisomerase_1	AT5G55300
TC06G001930	-0.17	-1.13	0.01	0.050	Putative_uncharacterized_protein	AT3G15840
TC00G020830	-0.17	-1.13	0.00	0.025	Putative_Porin%2C_eukaryotic_type	AT5G57490
TC07G009470	-0.17	-1.13	0.00	0.002	Uroporphyrinogen_decarboxylase_1,_chloroplastic	AT3G14930
TC05G002500	-0.17	-1.13	0.00	0.032	GDSL_esterase/lipase_CPRD49	AT2G38180
TC02G010130	-0.17	-1.13	0.01	0.048	ATP-dependent_Clp_protease_proteolytic_subunit-related_protein_1,_chloroplastic	AT1G49970
TC02G002990	-0.17	-1.13	0.00	0.006	Predicted_protein	AT5G24690
TC10G001430	-0.17	-1.13	0.01	0.039	Putative_uncharacterized_protein	AT3G55960
TC03G031270	-0.18	-1.13	0.00	0.001	ATP-dependent_Clp_protease_proteolytic_subunit_4,_chloroplastic	AT5G45390
TC08G004510	-0.18	-1.13	0.00	0.016	Cytoplasmic_tRNA_2-thiolation_protein_1	AT2G44270
TC02G026650	-0.18	-1.13	0.00	0.012	NADH_dehydrogenase_[ubiquinone]_flavoprotein_1,_mitochondrial	AT5G08530
TC09G034680	-0.18	-1.13	0.01	0.042	Putative_uncharacterized_protein	AT5G58430
TC09G006590	-0.18	-1.13	0.01	0.049	Putative_Nucleolysin_TIAR	AT1G17370
TC04G022640	-0.18	-1.14	0.01	0.045	Catalytic,_putative	AT4G14290
TC09G012650	-0.18	-1.14	0.01	0.050	Putative_uncharacterized_protein	AT4G20130
TC08G005720	-0.19	-1.14	0.00	0.006	Glucose-6-phosphate_isomerase	AT4G24620

TC03G014010	-0.19	-1.14	0.00	0.000	Adenosylhomocysteinase_1	AT3G23810
TC01G017980	-0.19	-1.14	0.01	0.046	NA	NA
TC02G034790	-0.19	-1.14	0.00	0.021	Predicted_protein	AT1G08660
TC10G004430	-0.19	-1.14	0.00	0.020	At1g28060/F13K9_16	AT1G28060
TC09G010880	-0.19	-1.14	0.01	0.045	Predicted_protein	AT3G25910
TC00G024300	-0.19	-1.14	0.01	0.046	Predicted_protein	AT4G27500
TC02G024160	-0.19	-1.14	0.00	0.010	Probable_LRR_receptor-like_serine/threonine-protein_kinase_At2g23950	AT1G60800
TC05G026350	-0.19	-1.14	0.01	0.041	Serine/threonine-protein_phosphatase_PP1	AT2G39840
TC04G016990	-0.19	-1.14	0.00	0.009	Acetolactate_synthase_small_subunit	AT2G31810
TC03G011540	-0.19	-1.14	0.00	0.027	SNAP25_homologous_protein_SNAP33	AT5G61210
TC03G005310	-0.19	-1.14	0.00	0.007	Putative_uncharacterized_protein	AT1G18170
TC08G000460	-0.19	-1.14	0.00	0.030	Predicted_protein	AT1G07700
TC01G030580	-0.19	-1.14	0.00	0.021	UDP-glucuronic_acid_decarboxylase_1	AT3G62830
TC01G004520	-0.20	-1.15	0.01	0.049	Serine/threonine-protein_kinase_SRK2I	AT5G66880
TC00G041300	-0.20	-1.15	0.00	0.029	Putative_Protein_sym-1	AT5G19750
TC03G027640	-0.20	-1.15	0.00	0.000	Putative_Aminopeptidase_N	AT1G63770
TC06G018700	-0.20	-1.15	0.00	0.005	Putative_Pentatricopeptide_repeat-containing_protein_At4g21170	AT4G21170
TC03G023650	-0.20	-1.15	0.01	0.042	Putative_uncharacterized_protein	AT5G46560
TC02G001650	-0.20	-1.15	0.00	0.013	Yth_domain-containing_protein,_putative	AT1G09810
TC01G024040	-0.20	-1.15	0.00	0.000	Putative_uncharacterized_protein	AT5G47860
TC02G008240	-0.20	-1.15	0.00	0.019	Predicted_protein	AT2G15860
TC05G028240	-0.20	-1.15	0.00	0.027	Putative_defensin-like_protein_184	AT3G05100
TC04G019060	-0.20	-1.15	0.01	0.042	Predicted_protein	AT1G05960
TC07G012860	-0.20	-1.15	0.00	0.009	Transcription_regulator,_putative	AT1G61730
TC01G001790	-0.20	-1.15	0.00	0.014	Putative_uncharacterized_protein	AT2G17970
TC02G017370	-0.21	-1.15	0.00	0.022	ATP_synthase_subunit_beta,_mitochondrial	AT5G08670
TC08G007800	-0.21	-1.15	0.00	0.018	Putative_Uncharacterized_protein_C757.02c	AT4G24220
TC05G029270	-0.21	-1.15	0.00	0.031	Putative_Xaa-Pro_aminopeptidase_1	AT3G05350
TC04G027170	-0.21	-1.15	0.00	0.021	Squamosa_promoter-binding-like_protein_7	AT5G18830
TC04G026240	-0.21	-1.16	0.00	0.000	Pre-mRNA-processing_factor_19_homolog_1	AT1G04510
TC05G004950	-0.21	-1.16	0.00	0.003	Putative_Retinol_dehydrogenase_12	AT2G37540
TC02G029160	-0.21	-1.16	0.00	0.002	Predicted_protein	AT1G14570
TC04G001160	-0.21	-1.16	0.00	0.024	DEAD-box_ATP-dependent_RNA_helicase_46	AT3G06480
TC00G047130	-0.21	-1.16	0.00	0.016	Glyceraldehyde-3-phosphate_dehydrogenase_A,_chloroplastic	AT1G12900

TC02G033270	-0.21	-1.16	0.00	0.026	Putative_Predicted_protein	NA
TC01G010180	-0.21	-1.16	0.00	0.026	Putative_Cytochrome_P450_86B 1	AT4G39490
TC01G025590	-0.21	-1.16	0.00	0.012	Succinyl-CoA_ligase_[GDP-forming]_subunit_beta,_mitochon drial	AT2G20420
TC10G005940	-0.21	-1.16	0.01	0.046	Putative_arsenical_pump-driving_ATPase	AT3G10350
TC01G026730	-0.21	-1.16	0.00	0.000	Transcription_factor_bHLH3	AT4G16430
TC04G020520	-0.21	-1.16	0.00	0.011	Probable_methylenetetrahydrofol ate_reductase	AT2G44160
TC01G005240	-0.21	-1.16	0.01	0.045	Probable_serine/threonine-protein_kinase_At1g01540	AT4G02630
TC05G002780	-0.22	-1.16	0.00	0.013	Auxin_transporter-like_protein_2	AT2G38120
TC09G007620	-0.22	-1.16	0.00	0.010	Casein_kinase_I_isoform_delta-like	AT4G26100
TC01G034380	-0.22	-1.16	0.00	0.004	Putative_uncharacterized_protein	AT2G46500
TC05G029440	-0.22	-1.16	0.01	0.043	Putative_Mitochondrial_Rho_GT Pase_1	AT5G27540
TC09G008410	-0.22	-1.16	0.00	0.002	Asparaginyl-tRNA_synthetase,_cytoplasmic_1	AT1G70980
TC01G007050	-0.22	-1.16	0.00	0.028	Branched-chain-amino-acid_aminotransferase_5,_chloro plastic	AT5G65780
TC07G014350	-0.22	-1.16	0.00	0.016	Hypothetical_protein	NA
TC03G017290	-0.22	-1.16	0.00	0.008	Putative_Splicing_factor_3A_sub unit_3	AT5G06160
TC03G001670	-0.22	-1.17	0.00	0.027	Putative_uncharacterized_protein	AT5G63050
TC01G006730	-0.22	-1.17	0.00	0.004	Serine_carboxypeptidase-like_18	AT1G33540
TC09G002390	-0.22	-1.17	0.00	0.000	DEAD-box_ATP-dependent_RNA_helicase_56	AT5G11200
TC02G027700	-0.22	-1.17	0.00	0.030	NA	NA
TC01G020450	-0.22	-1.17	0.00	0.004	Putative_uncharacterized_protein	AT5G48385
TC00G016810	-0.22	-1.17	0.00	0.002	Ring_finger_protein,_putative	NA
TC04G013440	-0.23	-1.17	0.00	0.005	Predicted_protein	AT1G66680
TC04G022720	-0.23	-1.17	0.00	0.021	Endo-1,3-1,4-beta-d-glucanase,_putative	AT3G23600
TC01G025370	-0.23	-1.17	0.00	0.002	Predicted_protein	AT4G21150
TC03G016690	-0.23	-1.17	0.01	0.049	Putative_uncharacterized_protein	AT5G51220
TC03G006240	-0.23	-1.17	0.01	0.040	Putative_Tetratricopeptide_repea t_protein_4_homolog	AT1G04130
TC00G045000	-0.23	-1.17	0.00	0.013	Hypothetical_protein	AT5G49970
TC05G020310	-0.23	-1.18	0.00	0.032	Putative_Protein_RRP5_homolog	AT3G11964
TC01G038370	-0.23	-1.18	0.00	0.037	Transcription_factor_UNE10	AT4G00050
TC07G017290	-0.23	-1.18	0.01	0.038	Succinate-semialdehyde_dehydrogenase,_mitochondrial	AT1G79440
TC01G001360	-0.24	-1.18	0.01	0.044	3-dehydroquinate_synthase	AT5G66120
TC00G037200	-0.24	-1.18	0.00	0.001	Pyruvate_dehydrogenase_E1_co mponent_subunit_alpha-1,_mitochondrial	AT1G24180
TC05G031090	-0.24	-1.18	0.00	0.030	Putative_Peptide_chain_release_factor_2	AT1G56350
TC05G018100	-0.24	-1.18	0.00	0.006	Putative_Transcription_factor_bH LH60	AT3G57800

TC07G002760	-0.24	-1.18	0.00	0.030	Putative_UPF0580_protein_C15orf58_homolog	AT4G26850
TC03G029930	-0.24	-1.18	0.00	0.005	Shikimate_kinase-like_protein	AT2G35500
TC00G017490	-0.24	-1.18	0.00	0.028	Coatomer_subunit_beta'-2	AT1G52360
TC05G030910	-0.24	-1.18	0.00	0.002	Importin_subunit_alpha-1	AT3G06720
TC05G025780	-0.24	-1.18	0.00	0.020	Putative_uncharacterized_protein	AT3G04310
TC00G068180	-0.24	-1.18	0.00	0.019	Lycopene_beta_cyclase,_chloroplastic/chromoplastic	AT3G10230
TC00G027760	-0.24	-1.18	0.00	0.020	Aminocarboxymuconate-semialdehyde_decarboxylase,_putative	NA
TC01G035460	-0.24	-1.18	0.00	0.032	50S_ribosomal_protein_L5,_chloroplastic	AT4G01310
TC01G007970	-0.24	-1.18	0.00	0.002	Protein_SGT1_homolog_At5g65490	AT5G65490
TC00G029100	-0.24	-1.18	0.00	0.001	Predicted_protein	AT3G13060
TC09G014700	-0.24	-1.18	0.00	0.018	Putative_Translation_initiation_factor_IF-3	AT4G30690
TC02G003530	-0.24	-1.18	0.00	0.006	Putative_Ubiquilin-1	AT2G17190
TC02G035030	-0.25	-1.19	0.00	0.020	DNA_repair_helicase_rad5,16,_putative	AT1G05120
TC09G030090	-0.25	-1.19	0.01	0.041	Putative_Anthocyanidin_3-O-glucosyltransferase_1	AT1G07260
TC09G003670	-0.25	-1.19	0.01	0.041	Putative_Eukaryotic_translation_initiation_factor_3_subunit_L	AT5G25757
TC05G003230	-0.25	-1.19	0.00	0.001	Predicted_protein	AT3G09210
TC03G019340	-0.25	-1.19	0.00	0.026	Putative_Post-GPI_attachment_to_proteins_factor_3	AT5G62130
TC03G026360	-0.25	-1.19	0.00	0.037	Predicted_protein	AT4G11970
TC09G025870	-0.25	-1.19	0.00	0.001	Sulfite_reductase_[ferredoxin]	AT5G04590
TC09G001430	-0.25	-1.19	0.01	0.042	Transmembrane_9_superfamily_member_4	AT5G25100
TC01G016830	-0.25	-1.19	0.00	0.003	Putative_Probable_U3_small_nucleolar_RNA-associated_protein_7	AT3G10530
TC10G002440	-0.25	-1.19	0.00	0.025	Serine/threonine-protein_phosphatase_PP1_isozyme_4	AT2G39840
TC02G032260	-0.25	-1.19	0.01	0.050	Putative_Protein_KTI12_homolog	AT1G13870
TC05G003800	-0.25	-1.19	0.01	0.042	Uracil_phosphoribosyltransferase	AT3G53900
TC00G032120	-0.25	-1.19	0.00	0.023	3-isopropylmalate_dehydrogenase,_chloroplastic	AT1G80560
TC03G026550	-0.25	-1.19	0.00	0.030	Eukaryotic_translation_initiation_factor_2_subunit_alpha	AT2G40290
TC00G056860	-0.25	-1.19	0.00	0.001	Putative_Zinc_finger_protein_CONSTANS-LIKE_16	AT1G25440
TC06G000130	-0.25	-1.19	0.00	0.030	Dynamin-2B	AT1G10290
TC00G049090	-0.25	-1.19	0.00	0.024	Biotin/lipoyl_attachment	AT1G52670
TC01G035210	-0.25	-1.19	0.01	0.043	Reticulon-like_protein_B5	AT2G46170
TC09G029070	-0.25	-1.19	0.00	0.010	Putative_Bifunctional_polymyxin_resistance_protein_arnA	AT1G08200
TC00G056010	-0.26	-1.19	0.00	0.003	Phosphatidylinositol-4-phosphate_5-kinase_9	AT3G09920
TC03G014740	-0.26	-1.19	0.00	0.014	SWI/SNF_complex_component_SNF12_homolog	AT5G14170

TC08G008280	-0.26	-1.19	0.00	0.000	Pyrophosphate--fructose_6-phosphate_1-phosphotransferase_subunit_alpha	AT1G20950
TC09G009270	-0.26	-1.20	0.00	0.000	Probable_lactoylglutathione_lyase,_chloroplast	AT1G67280
TC09G034600	-0.26	-1.20	0.00	0.030	Putative_Malonyl-CoA-acyl_carrier_protein_transacylase,_mitochondrial	AT2G30200
TC01G017100	-0.26	-1.20	0.00	0.008	Probable_S-acyltransferase_At4g15080	AT4G15080
TC03G020090	-0.26	-1.20	0.00	0.003	Putative_Fibrillarin	AT4G25630
TC05G028250	-0.26	-1.20	0.00	0.010	Predicted_protein	AT5G27950
TC01G035390	-0.26	-1.20	0.01	0.043	2-hydroxyacid_dehydrogenase,_putative	AT1G01510
TC10G005060	-0.26	-1.20	0.00	0.006	Elongation_factor_1-alpha	AT5G60390
TC09G014300	-0.26	-1.20	0.01	0.048	Putative_GDP-L-fucose_synthase_2	AT1G17890
TC02G007920	-0.26	-1.20	0.00	0.000	Transmembrane_9_superfamily_member_3	AT1G10950
TC07G004270	-0.26	-1.20	0.01	0.041	Putative_uncharacterized_protein	AT3G47860
TC09G031170	-0.26	-1.20	0.01	0.039	Putative_Uncharacterized_protein_At2g34460,_chloroplastic	AT3G46780
TC00G087380	-0.26	-1.20	0.00	0.030	IAA-amino_acid_hydrolase_ILR1-like_4	AT1G51760
TC04G021340	-0.26	-1.20	0.00	0.007	Predicted_protein	AT3G24160
TC04G013830	-0.26	-1.20	0.00	0.003	Putative_lipid_phosphate_phosphatase_3,_chloroplastic	AT3G02600
TC01G015050	-0.27	-1.20	0.00	0.014	Oxygen-evolving_enhancer_protein_2-1,_chloroplastic	AT1G06680
TC01G039330	-0.27	-1.20	0.01	0.049	Putative_uncharacterized_protein	AT4G16830
TC02G001520	-0.27	-1.20	0.00	0.002	Predicted_protein	AT1G33980
TC02G033190	-0.27	-1.20	0.00	0.037	Transcription_factor,_putative	AT1G69580
TC02G008000	-0.27	-1.20	0.00	0.034	Transcription_factor_RF2a,_putative	AT1G58110
TC07G008920	-0.27	-1.20	0.01	0.045	NA	NA
TC01G040090	-0.27	-1.20	0.00	0.032	Predicted_protein	AT3G63460
TC06G003420	-0.27	-1.20	0.01	0.049	Putative_COBW_domain-containing_protein_2	AT1G80480
TC01G030230	-0.27	-1.20	0.00	0.016	Putative_uncharacterized_protein	AT1G02910
TC03G020050	-0.27	-1.20	0.00	0.030	Prolyl-tRNA_synthetase	AT5G52520
TC02G034470	-0.27	-1.20	0.00	0.003	NA	NA
TC02G025750	-0.27	-1.21	0.00	0.020	ALBINO3-like_protein_1,_chloroplastic	AT1G24490
TC02G030620	-0.27	-1.21	0.01	0.039	50S_ribosomal_protein_L6,_chloroplastic	AT1G05190
TC03G002320	-0.27	-1.21	0.00	0.016	Predicted_protein	AT3G18580
TC01G031850	-0.27	-1.21	0.01	0.043	Pentatricopeptide_repeat-containing_protein_At1g02370,_mitochondrial	AT1G02370
TC04G010130	-0.27	-1.21	0.00	0.027	Probable_galacturonosyltransferase_13	AT3G01040
TC04G023400	-0.27	-1.21	0.00	0.000	Putative_Long-chain-fatty-acid--CoA_ligase_1	AT3G05970
TC01G031900	-0.27	-1.21	0.00	0.005	Pentatricopeptide_repeat-containing_protein_At2g31400,_c	AT2G31400

					hloroplastic	
TC05G002490	-0.27	-1.21	0.00	0.009	LIMR_family_protein_At5g01460	AT3G08930
TC04G004980	-0.27	-1.21	0.01	0.046	V-type_proton_ATPase_subunit_C	AT1G12840
TC02G002580	-0.27	-1.21	0.00	0.030	Seryl-tRNA_synthetase	AT5G27470
TC02G023290	-0.27	-1.21	0.00	0.022	Putative_Auxin-induced_protein_5NG4	AT5G40240
TC06G000230	-0.27	-1.21	0.01	0.039	Ferredoxin--NADP_reductase,_root-type_isozyme,_chloroplastic	AT4G05390
TC10G000790	-0.27	-1.21	0.00	0.002	Putative_uncharacterized_protein	AT3G56130
TC07G009520	-0.28	-1.21	0.00	0.004	Phosphoenolpyruvate_carboxylase,_housekeeping_isozyme	AT1G53310
TC01G028730	-0.28	-1.21	0.00	0.035	Putative_uncharacterized_protein	AT1G03270
TC08G003530	-0.28	-1.21	0.01	0.044	NA	NA
TC10G012330	-0.28	-1.21	0.00	0.026	Putative_Predicted_protein	AT5G07900
TC04G019680	-0.28	-1.21	0.00	0.037	Protein_TRIGALACTOSYLDIACYLGLYCEROL_2,_chloroplastic	AT3G20320
TC09G022160	-0.28	-1.21	0.00	0.002	Predicted_protein	AT3G57930
TC03G019700	-0.28	-1.21	0.00	0.000	Arginine/serine-rich-splicing_factor_RSP40	AT4G25500
TC10G007210	-0.28	-1.21	0.00	0.023	Putative_Uncharacterized_protein_SYNPCC7002_A1590	AT5G04440
TC07G010830	-0.28	-1.21	0.00	0.016	50S_ribosomal_protein_L13,_chloroplastic	AT1G78630
TC07G013200	-0.28	-1.21	0.00	0.002	Elongation_factor_2	AT1G56070
TC01G003740	-0.28	-1.21	0.00	0.002	Geranylgeranyl_pyrophosphate_synthase,_chloroplastic	AT4G36810
TC00G022440	-0.28	-1.21	0.01	0.046	ATP_binding_protein,_putative	AT1G65010
TC02G006260	-0.28	-1.21	0.00	0.036	tRNA-dihydrouridine_synthase_3-like	NA
TC05G019880	-0.28	-1.21	0.00	0.030	Predicted_protein_(Fragment)	AT2G43945
TC01G035920	-0.28	-1.21	0.00	0.011	U-box_domain-containing_protein_33	AT2G45910
TC09G016880	-0.28	-1.21	0.00	0.000	Predicted_protein	AT2G18750
TC03G020380	-0.28	-1.21	0.00	0.022	Predicted_protein	AT5G23890
TC04G022630	-0.28	-1.22	0.00	0.035	Glutamine_synthetase_leaf_isozyme,_chloroplastic	AT5G35630
TC06G014830	-0.28	-1.22	0.00	0.000	Magnesium-chelatase_subunit_chlI,_chloroplastic	AT4G18480
TC06G008030	-0.28	-1.22	0.01	0.049	Auxin-responsive_protein_IAA27	AT3G04730
TC08G005820	-0.28	-1.22	0.00	0.029	Putative_AP-4_complex_subunit_mu	AT4G24550
TC01G003290	-0.28	-1.22	0.00	0.000	Scarecrow-like_protein_4	AT5G66770
TC01G014900	-0.28	-1.22	0.00	0.030	Pentatricopeptide_repeat-containing_protein_At3g59040	AT3G59040
TC03G015400	-0.28	-1.22	0.00	0.024	Glutamate-1-semialdehyde_2,1-aminomutase_2,_chloroplastic	AT3G48730
TC00G010560	-0.28	-1.22	0.00	0.001	Calcium-dependent_protein_kinase_30	AT1G18890
TC04G001720	-0.29	-1.22	0.00	0.001	Hypothetical_protein	NA
TC01G019700	-0.29	-1.22	0.00	0.003	Putative_Transmembrane_9_superfamily_member_4	AT4G12650
TC03G013820	-0.29	-1.22	0.00	0.018	31_kDa_ribonucleoprotein,_chloroplastic	AT4G24770

TC02G024980	-0.29	-1.22	0.00	0.021	Putative_uncharacterized_protein	NA
TC02G000550	-0.29	-1.22	0.00	0.012	Putative_Bifunctional_purine_biosynthesis_protein_purH	AT2G35040
TC08G010880	-0.29	-1.22	0.00	0.020	Replication_factor_C_subunit_4	AT1G21690
TC04G018830	-0.29	-1.22	0.00	0.032	Epsin-2,_putative	NA
TC09G002670	-0.29	-1.22	0.00	0.007	Probable_cyclic_nucleotide-gated_ion_channel_5	AT2G23980
TC00G014640	-0.29	-1.22	0.00	0.021	Pantoate--beta-alanine_ligase	AT5G48840
TC05G022350	-0.29	-1.22	0.00	0.000	Cellulose_synthase-like_protein_D3	AT3G03050
TC02G023650	-0.29	-1.22	0.01	0.046	Quinone_oxidoreductase-like_protein_At1g23740,_chloroplastic	AT1G23740
TC06G020850	-0.29	-1.22	0.01	0.049	SNF1-related_protein_kinase_regulatory_subunit_beta-2	NA
TC03G014080	-0.29	-1.22	0.00	0.001	Putative_Protein_cbbY	AT3G48420
TC00G050720	-0.29	-1.22	0.00	0.000	Endoglucanase_25	AT5G49720
TC09G027090	-0.29	-1.22	0.00	0.016	Putative_Lysosomal_beta_glucosidase	AT5G20950
TC06G007890	-0.29	-1.22	0.00	0.012	NA	NA
TC08G010630	-0.29	-1.22	0.00	0.034	AT1G21600_protein	AT1G21600
TC02G009410	-0.29	-1.22	0.00	0.017	D-3-phosphoglycerate_dehydrogenase,_chloroplastic	AT4G34200
TC04G023500	-0.29	-1.22	0.00	0.030	Photosystem_I_reaction_center_subunit_II,_chloroplastic	AT4G02770
TC08G004420	-0.29	-1.22	0.00	0.011	Putative_Alpha-amylase	AT1G76130
TC06G019410	-0.29	-1.23	0.00	0.002	Aminomethyltransferase,_mitochondrial	AT1G11860
TC03G015980	-0.29	-1.23	0.00	0.013	Catalytic,_putative	AT5G61840
TC08G000220	-0.30	-1.23	0.00	0.012	Probable_serine/threonine-protein_kinase_At1g01540	AT1G01540
TC04G002840	-0.30	-1.23	0.00	0.029	SET_domain_protein	AT5G14260
TC04G013930	-0.30	-1.23	0.00	0.001	Putative_L-Ala-D/L-Glu_epimerase	AT3G18270
TC01G035320	-0.30	-1.23	0.01	0.049	Pentatricopeptide_repeat-containing_protein_At3g61520,_mitochondrial	AT3G61530
TC09G005220	-0.30	-1.23	0.00	0.023	Putative_Serine/threonine-protein_kinase_CBK1	AT4G33080
TC07G002000	-0.30	-1.23	0.01	0.049	Scarecrow-like_protein_5	AT1G50600
TC01G034960	-0.30	-1.23	0.00	0.033	Uncharacterized_protein_At4g01150,_chloroplastic	AT4G01150
TC01G041020	-0.30	-1.23	0.00	0.007	Predicted_protein	AT3G52150
TC04G024450	-0.30	-1.23	0.01	0.048	ATP_binding_protein,_putative	AT1G04210
TC06G003950	-0.30	-1.23	0.00	0.005	Rae1-like_protein_At1g80670	AT1G80670
TC04G017560	-0.30	-1.23	0.00	0.027	Protein_IQ-DOMAIN_14	AT2G43680
TC09G012820	-0.30	-1.23	0.00	0.000	Predicted_protein	AT2G12400
TC08G013850	-0.30	-1.23	0.00	0.026	Putative_Far_upstream_element-binding_protein_3	AT1G33680
TC00G020100	-0.30	-1.23	0.01	0.039	Putative_Cation/calcium_exchanger_4	AT5G17850
TC04G027510	-0.30	-1.23	0.00	0.006	Predicted_protein	AT5G18660

TC08G001400	-0.30	-1.23	0.01	0.042	Putative_Cytochrome_P450_82A3	AT4G31940
TC01G012850	-0.30	-1.23	0.01	0.041	Probable_NADH_dehydrogenase,_chloroplastic/mitochondrial	AT5G08740
TC02G009350	-0.30	-1.23	0.00	0.032	Putative_Kynurenine_formamidase	AT4G35220
TC01G026200	-0.30	-1.23	0.00	0.024	Putative_NADH_dehydrogenase_[ubiquinone]_1_alpha_subcomplex_subunit_9,_mitochondrial	AT2G20360
TC02G023250	-0.30	-1.23	0.00	0.030	Putative_U4/U6_small_nuclear_ribonucleoprotein_Prp31	AT1G60170
TC04G028990	-0.30	-1.23	0.00	0.019	Two-component_response_regulator_ARR2	AT4G16110
TC05G020970	-0.30	-1.23	0.00	0.000	Probable_metal-nicotianamine_transporter_YSL5	AT1G48370
TC08G003390	-0.30	-1.23	0.00	0.005	Filament-like_plant_protein_4	AT1G19835
TC10G002550	-0.30	-1.23	0.00	0.005	Putative_Folylpolyglutamate_synthase,_mitochondrial	AT3G55630
TC02G013990	-0.30	-1.23	0.00	0.000	Hypothetical_protein	AT3G18610
TC06G019820	-0.31	-1.24	0.00	0.029	50S_ribosomal_protein_L4	AT2G20060
TC04G015810	-0.31	-1.24	0.00	0.030	Putative_uncharacterized_protein	AT3G08600
TC05G026240	-0.31	-1.24	0.00	0.025	Pentatricopeptide_repeat-containing_protein_At1g08610	AT1G08610
TC01G011450	-0.31	-1.24	0.00	0.007	Putative_Zinc_finger_CCCH_domain-containing_protein_38	AT3G18640
TC09G030280	-0.31	-1.24	0.00	0.003	Phosphomethylpyrimidine_synthase	AT2G29630
TC00G022300	-0.31	-1.24	0.00	0.005	Putative_uncharacterized_protein	AT2G07360
TC05G006050	-0.31	-1.24	0.00	0.009	Probable_phosphoribosylformylglycinamidine_synthase,_chloroplastic	AT1G74260
TC02G031020	-0.31	-1.24	0.00	0.016	6-phosphogluconate_dehydrogenase,_decarboxylating	AT3G02360
TC02G014170	-0.31	-1.24	0.00	0.019	Putative_Zinc_finger_CCCH_domain-containing_protein_40	NA
TC05G007050	-0.31	-1.24	0.00	0.000	DP1	AT5G03415
TC05G019320	-0.31	-1.24	0.00	0.022	Putative_Programmed_cell_death_protein_2	AT4G02220
TC01G005460	-0.31	-1.24	0.00	0.024	Polyadenylate-binding_protein_2	AT1G49760
TC08G007170	-0.31	-1.24	0.00	0.014	Putative_uncharacterized_protein	AT3G49800
TC03G030630	-0.31	-1.24	0.00	0.012	Uncharacterized_protein_At4g19112.1	AT4G19110
TC01G031820	-0.31	-1.24	0.01	0.049	Putative_uncharacterized_protein	AT4G02010
TC02G000620	-0.31	-1.24	0.00	0.033	Hypothetical_protein	NA
TC00G000210	-0.31	-1.24	0.00	0.036	Biotin_carboxylase_1,_chloroplastic	AT5G35360
TC04G003930	-0.31	-1.24	0.01	0.038	H_-transporting_two-sector_ATPase,_alpha/beta_subunit,_central_region;_H_-transporting_two-sector_ATPase,_delta/epsilon_subunit	ATCG00480
TC01G035870	-0.32	-1.24	0.00	0.000	Aquaporin_PIP1-2	AT1G01620
TC08G003640	-0.32	-1.24	0.00	0.000	Tubulin_beta-6_chain	AT1G75780
TC10G015920	-0.32	-1.24	0.00	0.004	Uroporphyrinogen_decarboxylase,_chloroplastic	AT2G40490
TC00G028210	-0.32	-1.24	0.01	0.040	Predicted_protein	AT3G57880

TC04G030270	-0.32	-1.25	0.00	0.004	Yth_domain-containing_protein,_putative	AT1G48110
TC03G029400	-0.32	-1.25	0.00	0.035	Putative_Nephrocystin-3	AT4G10840
TC04G017460	-0.32	-1.25	0.00	0.016	Cysteine_synthase,_chloroplastic/chromoplastic	AT2G43750
TC09G003510	-0.32	-1.25	0.00	0.000	GTP-binding_protein_At2g22870	AT5G11480
TC04G025310	-0.32	-1.25	0.00	0.005	1-aminocyclopropane-1-carboxylate_oxidase_homolog_1	AT5G10060
TC09G033490	-0.32	-1.25	0.00	0.005	Putative_uncharacterized_protein	AT1G07990
TC01G017460	-0.32	-1.25	0.00	0.000	Putative_uncharacterized_protein_yghX	AT2G32520
TC01G026020	-0.32	-1.25	0.00	0.015	Putative_G3BP-like_protein	AT5G43960
TC01G002060	-0.32	-1.25	0.01	0.039	Putative_Lactoylglutathione_lyase	AT1G15380
TC04G002710	-0.32	-1.25	0.00	0.021	Prohibitin-1,_mitochondrial	AT3G27280
TC01G002090	-0.32	-1.25	0.00	0.030	Probable_fructose-bisphosphate_aldolase_1,_chloroplastic	AT4G38970
TC07G005140	-0.32	-1.25	0.01	0.043	Predicted_protein_(Fragment)	AT3G20680
TC10G014000	-0.32	-1.25	0.00	0.011	Probable_protein_arginine_N-methyltransferase_3	AT3G12270
TC03G024470	-0.32	-1.25	0.00	0.021	Plasminogen_activator_inhibitor_1_RNA-binding_protein,_putative	AT4G16830
TC03G014430	-0.33	-1.25	0.00	0.010	Putative_uncharacterized_protein_At3g48500	AT3G48500
TC02G006530	-0.33	-1.25	0.01	0.047	Putative_Sec14_cytosolic_factor	AT2G21520
TC07G014720	-0.33	-1.25	0.00	0.029	Predicted_protein	AT1G79090
TC03G029950	-0.33	-1.25	0.00	0.022	Predicted_protein	NA
TC00G014920	-0.33	-1.26	0.01	0.038	Dead_box_ATP-dependent_RNA_helicase,_putative	AT4G09730
TC01G037750	-0.33	-1.26	0.01	0.041	Pentatricopeptide_repeat-containing_protein_At2g45350,_chloroplastic	AT2G45350
TC00G045910	-0.33	-1.26	0.00	0.014	Predicted_protein	AT3G12060
TC07G010310	-0.33	-1.26	0.00	0.031	Putative_uncharacterized_protein	AT3G14860
TC05G029590	-0.33	-1.26	0.01	0.045	Putative_Predicted_protein	NA
TC02G000970	-0.33	-1.26	0.00	0.003	DNA_damage-binding_protein_1	AT4G05420
TC04G020060	-0.33	-1.26	0.00	0.025	Putative_Protein_transport_protein_SEC23	AT1G05520
TC01G000350	-0.33	-1.26	0.00	0.000	Glutamate_receptor_3.6	AT3G51480
TC09G006760	-0.33	-1.26	0.00	0.032	Putative_Adipocyte_plasma_membrane-associated_protein	NA
TC01G031790	-0.33	-1.26	0.00	0.000	ATP-dependent_Clp_protease_proteolytic_subunit_5,_chloroplastic	AT1G02560
TC08G004790	-0.33	-1.26	0.00	0.012	Putative_uncharacterized_protein	AT1G63610
TC05G005640	-0.34	-1.26	0.00	0.013	Putative_SAP_domain-containing_ribonucleoprotein	AT5G02770
TC01G008540	-0.34	-1.26	0.00	0.014	Putative_tRNA_(cytosine-5-)-methyltransferase_NSUN2	AT2G22400
TC07G006570	-0.34	-1.26	0.00	0.020	Putative_Sorting_and_assembly_machinery_component_50_homolog	AT5G05520
TC00G014190	-0.34	-1.26	0.00	0.013	Putative_metal_tolerance_protein_C3	AT3G58060
TC00G002000	-0.34	-1.26	0.01	0.047	Arginase	AT4G08900

TC02G008580	-0.34	-1.26	0.01	0.046	Predicted_protein	AT3G19130
TC06G001570	-0.34	-1.27	0.00	0.014	DEAD-box_ATP-dependent_RNA_helicase_56	AT5G11200
TC04G012070	-0.34	-1.27	0.00	0.022	DNA-directed_RNA_polymerase_2,_chloroplastic/mitochondrial	AT5G15700
TC04G024170	-0.34	-1.27	0.00	0.003	NA	NA
TC03G020150	-0.34	-1.27	0.00	0.003	Putative_uncharacterized_protein	AT4G25620
TC00G080410	-0.34	-1.27	0.00	0.025	Uncharacterized_membrane_protein_At1g16860	AT1G16860
TC00G075300	-0.34	-1.27	0.00	0.017	Putative_uncharacterized_protein	AT1G59840
TC03G024830	-0.34	-1.27	0.00	0.036	Putative_Formyltetrahydrofolate_deformylase	AT4G17360
TC03G012440	-0.34	-1.27	0.00	0.020	Putative_Cleavage_stimulation_factor_subunit_1	AT5G60940
TC02G006520	-0.34	-1.27	0.01	0.045	Putative_Ocs_element-binding_factor_1	AT1G75390
TC00G059510	-0.34	-1.27	0.00	0.009	Vacuolar_cation/proton_exchanger_2	NA
TC00G005690	-0.34	-1.27	0.00	0.030	Putative_Bystin	NA
TC01G030930	-0.35	-1.27	0.00	0.024	Predicted_protein	AT4G02260
TC02G016150	-0.35	-1.27	0.00	0.009	Prohibitin-2	AT4G28510
TC00G026380	-0.35	-1.27	0.00	0.001	Glutamate_decarboxylase	AT2G02010
TC02G013010	-0.35	-1.27	0.00	0.011	Predicted_protein	AT5G08415
TC01G012200	-0.35	-1.27	0.01	0.046	Probable_methyltransferase_PMT26	AT5G64030
TC09G028810	-0.35	-1.27	0.00	0.029	Putative_Probable_phosphoglycerate_mutase_gpmB	AT5G22620
TC00G069140	-0.35	-1.27	0.00	0.001	Putative_UPF0176_protein_pc0378	AT1G17850
TC02G034690	-0.35	-1.27	0.00	0.001	Protein_CHUP1,_chloroplastic	AT3G25690
TC04G023970	-0.35	-1.27	0.01	0.039	Predicted_protein	AT3G22550
TC09G011030	-0.35	-1.27	0.00	0.008	NA	NA
TC04G020730	-0.35	-1.27	0.00	0.009	Predicted_protein	AT3G23910
TC06G011030	-0.35	-1.27	0.00	0.032	Putative_uncharacterized_protein	AT4G31440
TC02G012200	-0.35	-1.28	0.01	0.040	Amine_oxidase,_putative	AT3G09580
TC05G004750	-0.35	-1.28	0.00	0.029	Acetylglucosaminyltransferase,_putative	NA
TC05G006520	-0.35	-1.28	0.00	0.000	Ribonucleoprotein_At2g37220,_chloroplastic	AT2G37220
TC02G014000	-0.35	-1.28	0.00	0.028	Predicted_protein	AT1G50120
TC09G009370	-0.36	-1.28	0.00	0.022	Elongation_factor_Ts	AT4G29060
TC05G005260	-0.36	-1.28	0.00	0.032	Putative_uncharacterized_protein	AT2G37400
TC09G022300	-0.36	-1.28	0.00	0.000	Lipoxygenase_2,_chloroplastic	AT3G45140
TC07G000190	-0.36	-1.28	0.01	0.050	Probable_WRKY_transcription_factor_2	NA
TC04G017700	-0.36	-1.29	0.00	0.013	Lysyl-tRNA_synthetase	AT3G11710
TC02G007260	-0.36	-1.29	0.00	0.005	Putative_Translation_initiation_factor_IF-2	AT4G11160
TC02G003430	-0.36	-1.29	0.00	0.008	Actin-101	AT5G09810
TC08G012990	-0.36	-1.29	0.00	0.004	Thioredoxin-like_protein_slr0233	AT1G76760

TC09G009660	-0.36	-1.29	0.01	0.042	Chloroplast_processing_peptidase	AT3G24590
TC10G009100	-0.36	-1.29	0.00	0.019	Putative_uncharacterized_protein	NA
TC04G029030	-0.36	-1.29	0.01	0.050	Importin_subunit_alpha-1	AT4G16143
TC09G032960	-0.36	-1.29	0.01	0.040	Mitogen-activated_protein_kinase_homolog_NTF6	AT1G07880
TC06G015710	-0.36	-1.29	0.00	0.000	Putative_Serine/threonine-protein_kinase_HT1	AT5G58950
TC08G000150	-0.36	-1.29	0.01	0.047	Polyadenylate-binding_protein_2	AT1G49760
TC03G002290	-0.37	-1.29	0.00	0.000	Elongation_factor_Tu,_chloroplastic	AT4G20360
TC05G023510	-0.37	-1.29	0.00	0.022	Putative_amidase_C869.01	AT4G34880
TC00G027040	-0.37	-1.29	0.00	0.023	Probable_methyltransferase_PMT25	AT1G29470
TC05G023650	-0.37	-1.29	0.00	0.007	Putative_Probable_LRR_receptor-like_serine/threonine-protein_kinase_At1g14390	AT3G03770
TC01G001330	-0.37	-1.29	0.00	0.017	Putative_Vacuolar_protein_sorting-associated_protein_35	AT2G17790
TC08G008900	-0.37	-1.29	0.00	0.005	Probable_pectin_methyltransferase_QUA2	AT1G78240
TC02G029530	-0.37	-1.29	0.00	0.006	Putative_uncharacterized_protein	AT1G68130
TC03G010410	-0.37	-1.29	0.00	0.016	Eukaryotic_peptide_chain_release_factor_subunit_1-3	AT5G47880
TC03G023410	-0.37	-1.29	0.00	0.031	NA	NA
TC06G009910	-0.37	-1.29	0.00	0.000	Putative_uncharacterized_protein	AT3G13990
TC00G013030	-0.37	-1.29	0.00	0.035	Putative_uncharacterized_protein	NA
TC03G011020	-0.37	-1.29	0.00	0.009	Malate_dehydrogenase,_chloroplastic	AT3G47520
TC09G007960	-0.37	-1.29	0.00	0.013	Putative_Glutamate-rich_WD_repeat-containing_protein_1	AT2G19540
TC02G012620	-0.37	-1.29	0.00	0.023	Glutamyl-tRNA_synthetase	AT5G64050
TC09G018550	-0.37	-1.29	0.01	0.048	Hypothetical_protein	NA
TC03G003970	-0.37	-1.29	0.00	0.005	Ring_finger_containing_protein,_putative	AT1G17970
TC06G020730	-0.37	-1.29	0.00	0.009	NA	NA
TC06G000140	-0.37	-1.29	0.00	0.029	Putative_U3_small_nucleolar_RNA-interacting_protein_2	AT4G21130
TC00G029750	-0.37	-1.30	0.00	0.026	DEAD-box_ATP-dependent_RNA_helicase_37	AT2G42520
TC05G027350	-0.37	-1.30	0.01	0.050	Auxin-responsive_protein_IAA16	AT3G04730
TC04G010680	-0.37	-1.30	0.01	0.047	Putative_G3BP-like_protein	AT5G60980
TC01G039950	-0.38	-1.30	0.00	0.005	Putative_uncharacterized_protein	AT2G44640
TC01G034580	-0.38	-1.30	0.00	0.008	DEAD-box_ATP-dependent_RNA_helicase_26	AT5G63630
TC10G000060	-0.38	-1.30	0.00	0.002	Ribose-phosphate_pyrophosphokinase_5	AT2G44530
TC03G030370	-0.38	-1.30	0.00	0.010	Putative_uncharacterized_protein	AT1G32080
TC02G000950	-0.38	-1.30	0.01	0.040	Eukaryotic_translation_initiation_factor_3_subunit_B	AT5G25780
TC03G001710	-0.38	-1.30	0.00	0.026	NA	NA
TC05G005020	-0.38	-1.30	0.01	0.049	Arginine_biosynthesis_bifunctional_protein_argJ	AT2G37500

TC04G020470	-0.38	-1.30	0.00	0.008	Serine_hydroxymethyltransferase_1	AT4G13930
TC02G006550	-0.38	-1.30	0.00	0.015	Diaminopimelate_decarboxylase_2,_chloroplastic	AT3G14390
TC07G012790	-0.38	-1.30	0.00	0.029	Probable_peroxisomal_(S)-2-hydroxy-acid_oxidase_2	AT3G14420
TC03G030450	-0.39	-1.31	0.01	0.050	Isoflavone_reductase_homolog	AT1G32100
TC06G020660	-0.39	-1.31	0.00	0.030	Transcriptional_adapter_ADA2b	NA
TC05G005760	-0.39	-1.31	0.00	0.018	Pentatricopeptide_repeat-containing_protein_At5g02830,_chloroplastic	AT5G02830
TC01G002380	-0.39	-1.31	0.00	0.004	Beta-galactosidase_3	AT1G45130
TC05G028470	-0.39	-1.31	0.00	0.031	Putative_uncharacterized_protein	AT1G55370
TC02G001340	-0.39	-1.31	0.00	0.024	12-oxophytodienoate_reductase_1	AT1G76680
TC03G026840	-0.39	-1.31	0.00	0.005	Predicted_protein	AT1G63120
TC03G028960	-0.39	-1.31	0.00	0.010	Putative_E3_ubiquitin-protein_ligase_UBR7	AT4G23860
TC08G006720	-0.39	-1.31	0.00	0.000	Inositol-tetrakisphosphate_1-kinase_2	AT4G08170
TC05G018890	-0.39	-1.31	0.00	0.028	Putative_uncharacterized_protein_T10K17.190	AT2G42150
TC02G000990	-0.39	-1.31	0.00	0.000	ABC_transporter_C_family_member_2	AT2G34660
TC03G002580	-0.39	-1.31	0.00	0.000	Putative_Aspartic_proteinase_nepenthesin-1	AT1G25510
TC02G019690	-0.39	-1.31	0.01	0.048	NA	NA
TC05G031960	-0.39	-1.31	0.00	0.004	NA	NA
TC09G030170	-0.39	-1.31	0.00	0.017	Predicted_protein	AT1G07280
TC02G024400	-0.39	-1.31	0.00	0.001	NA	NA
TC02G000520	-0.39	-1.31	0.00	0.004	Probable_UDP-N-acetylglucosamine_pyrophosphorylase	AT1G31070
TC06G003200	-0.40	-1.32	0.00	0.018	Putative_Haloalkane_dehalogenase	AT1G52510
TC09G015240	-0.40	-1.32	0.00	0.005	UDP-glucuronate_4-epimerase_1	AT4G30440
TC02G015410	-0.40	-1.32	0.00	0.000	Mannan_endo-1,4-beta-mannosidase_2	AT2G20680
TC06G020840	-0.40	-1.32	0.01	0.041	Oligopeptide_transporter_3	AT4G16370
TC08G005360	-0.40	-1.32	0.00	0.001	Monosaccharide-sensing_protein_2	AT1G20840
TC07G002060	-0.40	-1.32	0.00	0.013	NA	NA
TC03G019660	-0.40	-1.32	0.00	0.024	Putative_uncharacterized_protein	AT5G52100
TC09G031410	-0.40	-1.32	0.00	0.019	Predicted_protein	AT5G59500
TC00G089630	-0.40	-1.32	0.00	0.000	ATPase_11,_plasma_membrane-type	AT5G62670
TC10G000540	-0.40	-1.32	0.00	0.022	Putative_uncharacterized_protein	AT2G38780
TC00G084630	-0.40	-1.32	0.01	0.040	Probable_rhamnose_biosynthetic_enzyme_1	AT1G78570
TC02G029750	-0.40	-1.32	0.00	0.005	Probable_xyloglucan_endotransglucosylase/hydrolase_protein_28	AT1G14720
TC09G011790	-0.40	-1.32	0.00	0.019	Predicted_protein	NA
TC01G013570	-0.40	-1.32	0.00	0.005	Ketol-acid_reductoisomerase,_chloroplastic	AT3G58610
TC02G028230	-0.40	-1.32	0.00	0.006	Putative_Thiol:disulfide_interchange_protein_txlA_homolog	AT4G37200

TC08G007770	-0.40	-1.32	0.00	0.037	Putative_uncharacterized_protein	AT1G27700
TC03G027610	-0.40	-1.32	0.01	0.046	Cytochrome_P450_86A2	AT1G63710
TC08G014190	-0.40	-1.32	0.00	0.003	Predicted_protein	AT1G22882
TC02G004670	-0.40	-1.32	0.00	0.028	Putative_uncharacterized_protein	AT1G34360
TC02G034540	-0.40	-1.32	0.00	0.035	Methionine_aminopeptidase_1B,_chloroplastic	AT1G13270
TC03G029070	-0.41	-1.33	0.00	0.004	GDSL_esterase/lipase_At4g10955	AT2G05260
TC03G000790	-0.41	-1.33	0.01	0.050	Probable_RNA-dependent_RNA_polymerase_2	AT4G11130
TC03G025050	-0.41	-1.33	0.00	0.013	Chaperone_protein_dnaJ_13	AT2G35720
TC08G008550	-0.41	-1.33	0.00	0.020	Putative_28_kDa_ribonucleoprotein,_chloroplastic	AT4G09040
TC06G019200	-0.41	-1.33	0.00	0.000	ATP_synthase_gamma_chain,_chloroplastic	AT4G04640
TC01G028890	-0.41	-1.33	0.00	0.014	Elongation_factor_Tu,_mitochondrial	AT4G02930
TC03G023530	-0.41	-1.33	0.00	0.000	Pyruvate_kinase_isozyme_G,_chloroplastic	AT1G32440
TC10G008440	-0.41	-1.33	0.00	0.034	Putative_Bark_storage_protein_A	AT4G24350
TC04G002670	-0.41	-1.33	0.00	0.024	Glucose-6-phosphate_1-dehydrogenase,_cytoplasmic_isoform	AT3G27300
TC02G016420	-0.41	-1.33	0.00	0.007	Protein_binding_protein,_putative	AT4G28370
TC08G002130	-0.41	-1.33	0.01	0.046	Probable_LRR_receptor-like_serine/threonine-protein_kinase_At2g16250	AT2G16250
TC02G014650	-0.41	-1.33	0.00	0.035	Protein_phosphatase_1_regulatory_subunit,_putative	AT4G03260
TC01G011220	-0.42	-1.33	0.00	0.001	Uridylate_kinase	AT3G18680
TC08G002590	-0.42	-1.33	0.00	0.022	Mitogen-activated_protein_kinase_homolog_NTF3	AT1G10210
TC00G026520	-0.42	-1.34	0.00	0.011	Putative_phagocytic_receptor_1b	AT2G01970
TC05G025090	-0.42	-1.34	0.00	0.019	Violaxanthin_de-epoxidase,_chloroplastic	AT1G08550
TC01G007950	-0.42	-1.34	0.00	0.005	Nucleobase-ascorbate_transporter_11	AT4G38050
TC08G004270	-0.42	-1.34	0.00	0.033	Tetracycline_transporter,_putative	AT2G16990
TC01G029360	-0.43	-1.34	0.00	0.002	Gtpase_activating_protein,_putative	AT4G03100
TC09G030210	-0.43	-1.34	0.00	0.011	Sodium-dependent_phosphate_transport_protein_1,_chloroplastic	AT2G29650
TC01G008500	-0.43	-1.34	0.00	0.005	Putative_LanC-like_protein_2	AT5G65280
TC10G012980	-0.43	-1.35	0.01	0.046	Putative_uncharacterized_protein	AT3G12390
TC01G012610	-0.43	-1.35	0.00	0.002	At4g33480	AT4G33480
TC10G013470	-0.43	-1.35	0.00	0.026	Putative_Flavonol_sulfotransferase-like	AT5G07010
TC05G031690	-0.43	-1.35	0.00	0.025	Probable_receptor-like_protein_kinase_At2g42960	AT1G56720
TC01G017530	-0.43	-1.35	0.01	0.041	NA	NA
TC00G090890	-0.44	-1.35	0.00	0.000	NA	NA
TC09G007590	-0.44	-1.35	0.01	0.049	Eukaryotic_initiation_factor_4A-15	AT3G13920
TC01G002570	-0.44	-1.35	0.00	0.004	Predicted_protein_(Fragment)	AT3G26750

TC02G012490	-0.44	-1.35	0.00	0.032	Predicted_protein_(Fragment)	AT4G38225
TC03G025560	-0.44	-1.36	0.01	0.042	Putative_UPF0551_protein_C8orf38_homolog,_mitochondrial	AT1G62730
TC02G000690	-0.44	-1.36	0.00	0.016	Coatomer_subunit_delta	AT5G05010
TC08G003480	-0.44	-1.36	0.00	0.033	NA	NA
TC01G036400	-0.44	-1.36	0.01	0.050	Bifunctional_protein_folD	AT4G00620
TC05G023220	-0.44	-1.36	0.00	0.008	Putative_uncharacterized_protein	AT5G17710
TC00G018080	-0.45	-1.36	0.01	0.040	Caffeic_acid_3-O-methyltransferase	AT5G54160
TC09G030990	-0.45	-1.36	0.00	0.035	Serine/threonine-protein_phosphatase_PP1_isozyme_2	AT2G39840
TC09G003520	-0.45	-1.36	0.00	0.021	Predicted_protein	AT5G11490
TC05G028430	-0.45	-1.36	0.00	0.030	Short-chain_type_dehydrogenase/reductase	AT4G13180
TC09G001560	-0.45	-1.36	0.00	0.019	Nucleic_acid_binding_protein,_putative	AT4G31880
TC01G013500	-0.45	-1.36	0.00	0.027	Predicted_protein	AT1G78710
TC01G037950	-0.45	-1.36	0.00	0.002	Putative_Auxin-induced_protein_X10A	AT3G60690
TC00G050290	-0.45	-1.37	0.01	0.045	Somatic_embryogenesis_receptor_kinase_1	AT1G34210
TC03G027850	-0.45	-1.37	0.01	0.040	NA	NA
TC06G006840	-0.45	-1.37	0.00	0.000	Plastidic_ATP/ADP-transporter	AT1G15500
TC06G003340	-0.45	-1.37	0.00	0.002	Pyrophosphate-energized_vacuolar_membrane_proton_pump	AT1G15690
TC02G029200	-0.46	-1.37	0.00	0.000	Zinc_finger_protein,_putative	AT2G02070
TC01G036920	-0.46	-1.37	0.00	0.016	Putative_Adenosine_3'-phospho_5'-phosphosulfate_transporter_2	AT1G12600
TC05G020770	-0.46	-1.37	0.00	0.000	Photosystem_I_reaction_center_subunit_XI,_chloroplastic	AT4G12800
TC06G018840	-0.46	-1.37	0.00	0.015	Beta-glucosidase_46	AT1G61820
TC10G002220	-0.46	-1.37	0.00	0.020	Putative_Solute_carrier_family_35_member_E4	AT5G05820
TC05G001560	-0.46	-1.37	0.01	0.049	NA	NA
TC03G001060	-0.46	-1.38	0.00	0.012	Serine/threonine-protein_kinase_HT1	AT1G62400
TC01G009060	-0.46	-1.38	0.00	0.003	Predicted_protein	AT4G39840
TC08G002560	-0.46	-1.38	0.00	0.037	Putative_Cyclin-T1-4	AT1G27630
TC09G030140	-0.47	-1.38	0.00	0.000	Anthranilate_synthase_component_I-2,_chloroplastic	AT2G29690
TC03G023330	-0.47	-1.38	0.00	0.017	Putative_uncharacterized_protein	NA
TC08G002770	-0.47	-1.38	0.00	0.000	Beta-galactosidase_5	AT1G45130
TC03G002170	-0.47	-1.38	0.00	0.013	Putative_Protein_MAK16_homolog_B	AT1G23280
TC09G030100	-0.47	-1.39	0.00	0.007	NA	NA
TC01G018100	-0.47	-1.39	0.00	0.025	Rho_GDP-dissociation_inhibitor_1	NA
TC01G015140	-0.47	-1.39	0.00	0.002	Glutathione_S-transferase_ERD13	AT2G30860
TC03G028380	-0.47	-1.39	0.00	0.017	6-phosphogluconate_dehydrogenase,_decarboxylating	AT5G41670

TC03G017410	-0.47	-1.39	0.00	0.009	Spermidine_synthase	AT5G53120
TC10G001820	-0.47	-1.39	0.01	0.042	Putative_Jasmonate_O-methyltransferase	AT1G19640
TC04G020920	-0.48	-1.40	0.00	0.012	DUF246_domain-containing_protein_At1g04910	AT1G04910
TC02G033690	-0.48	-1.40	0.00	0.017	Cullin-3	AT1G26830
TC04G002380	-0.49	-1.40	0.00	0.003	Uncharacterized_membrane_protein_At3g27390	AT5G40640
TC02G012690	-0.49	-1.40	0.00	0.009	NA	NA
TC02G003180	-0.49	-1.41	0.00	0.012	Ptm1,_putative	AT2G01070
TC02G029590	-0.49	-1.41	0.00	0.000	PLE,_putative	AT2G01910
TC00G054420	-0.50	-1.41	0.00	0.000	NA	NA
TC08G011180	-0.50	-1.41	0.01	0.046	NA	NA
TC06G002620	-0.50	-1.41	0.00	0.035	D-3-phosphoglycerate_dehydrogenase,_chloroplastic	AT1G17745
TC00G043210	-0.50	-1.41	0.00	0.008	RING-H2_finger_protein_ATL4E	NA
TC03G014200	-0.50	-1.41	0.00	0.032	Putative_Oxygen-independent_coproporphyrinogen-III_oxidase-like_protein_sll1917	AT5G63290
TC09G029270	-0.50	-1.41	0.00	0.035	Serine_carboxypeptidase-like_51	AT2G27920
TC03G029820	-0.51	-1.42	0.00	0.005	50S_ribosomal_protein	AT4G23620
TC07G016580	-0.51	-1.42	0.00	0.003	Phosphoglycerate_kinase,_chloroplastic	AT1G56190
TC09G006370	-0.51	-1.42	0.00	0.000	Sucrose_synthase	AT3G43190
TC00G016020	-0.51	-1.42	0.00	0.032	Hypothetical_protein	NA
TC07G006640	-0.51	-1.43	0.01	0.039	Fiber_protein_Fb34	AT4G27435
TC05G008920	-0.51	-1.43	0.00	0.005	Cell_division_protein_ftsZ	AT2G36250
TC07G000720	-0.52	-1.43	0.01	0.048	Putative_uncharacterized_protein	AT5G55970
TC01G005590	-0.52	-1.44	0.00	0.000	Glycosyltransferase	AT5G67230
TC09G034000	-0.52	-1.44	0.00	0.016	Serine_carboxypeptidase-like_48	AT3G10410
TC02G026330	-0.52	-1.44	0.00	0.019	Serine/threonine_protein_phosphatase_2A_59_kDa_regulatory_subunit_B'_eta_isoform	NA
TC02G023850	-0.52	-1.44	0.00	0.014	Putative_Glycogenin-1	AT1G60470
TC03G021000	-0.52	-1.44	0.00	0.007	Predicted_protein	AT3G49260
TC08G009610	-0.52	-1.44	0.00	0.006	Membrane-anchored_ubiquitin-fold_protein_6	AT1G77870
TC09G013330	-0.53	-1.44	0.01	0.045	Probable_protein_phosphatase_2C_22	NA
TC10G000880	-0.53	-1.44	0.00	0.035	Putative_uncharacterized_protein	AT2G40320
TC01G015100	-0.53	-1.44	0.00	0.008	NA	NA
TC06G010940	-0.53	-1.44	0.00	0.009	4-coumarate--CoA_ligase_1	AT1G51680
TC00G027180	-0.53	-1.44	0.00	0.020	Polycomb_group_protein_VERNALIZATION_2	AT4G16845
TC02G032060	-0.53	-1.44	0.00	0.013	Pentatricopeptide_repeat-containing_protein_At1g69290	AT1G69290
TC02G024910	-0.53	-1.45	0.00	0.000	Putative_Root_phototropism_protein_3	AT1G67900
TC02G004390	-0.54	-1.45	0.00	0.000	Putative_Hippocampus_abundant_transcript_1_protein	NA
TC02G006970	-0.54	-1.46	0.00	0.000	Hypothetical_protein	AT2G21660

TC07G000770	-0.55	-1.46	0.00	0.018	NA	NA
TC10G001550	-0.55	-1.47	0.00	0.016	Putative_uncharacterized_protein	AT2G40080
TC09G010020	-0.55	-1.47	0.00	0.000	Aldehyde_dehydrogenase_family_2_member_C4	AT3G24503
TC08G003420	-0.55	-1.47	0.00	0.006	NA	NA
TC05G007120	-0.55	-1.47	0.00	0.018	S-adenosylmethionine_synthase_2	AT2G36880
TC04G003840	-0.56	-1.47	0.00	0.019	Apocytochrome_f	ATCG00540
TC00G020080	-0.56	-1.47	0.00	0.027	Putative_Sulfotransferase_16	AT5G07010
TC08G008560	-0.56	-1.48	0.00	0.017	Blue_copper_protein	AT1G22480
TC10G016710	-0.56	-1.48	0.00	0.006	NA	NA
TC08G002080	-0.57	-1.48	0.00	0.003	Predicted_protein	AT5G42760
TC09G011610	-0.57	-1.49	0.00	0.006	Putative_Glucan_endo-1,3-beta-glucosidase_11	AT3G15800
TC07G004790	-0.57	-1.49	0.00	0.002	Thiazole_biosynthetic_enzyme,_chloroplastic	AT5G54770
TC00G084610	-0.58	-1.49	0.00	0.000	Alpha,alpha-trehalose-phosphate_synthase_[UDP-forming]_1	AT1G78580
TC05G000150	-0.58	-1.49	0.00	0.002	Putative_Probable_UDP-sugar_transporter_protein_SLC35A4	AT3G43740
TC06G016510	-0.58	-1.49	0.00	0.007	Pentatricopeptide_repeat-containing_protein_At3g02650,_mitochondrial	AT3G02650
TC04G003810	-0.58	-1.50	0.00	0.009	Photosystem_II_CP47_chlorophyll_apoprotein	ATCG00680
TC04G022740	-0.58	-1.50	0.00	0.015	Ribonucleoside-diphosphate_reductase_small_chain_A	AT3G23580
TC01G000260	-0.59	-1.50	0.00	0.029	NA	NA
TC06G003470	-0.59	-1.50	0.00	0.016	CTV.2	AT1G80490
TC01G008970	-0.59	-1.51	0.00	0.004	Asparagine_synthetase_[glutamine-hydrolyzing]	AT5G65010
TC03G026830	-0.60	-1.51	0.01	0.045	Putative_uncharacterized_protein	AT4G23060
TC00G074080	-0.60	-1.51	0.00	0.000	Glycyl-tRNA_synthetase_1,_mitochondrial	AT1G29880
TC04G000540	-0.60	-1.51	0.00	0.011	Putative_Auxin-induced_protein_5NG4	AT5G40230
TC09G004900	-0.60	-1.51	0.00	0.004	Predicted_protein	AT5G11890
TC08G000350	-0.61	-1.52	0.00	0.000	NA	NA
TC05G013890	-0.61	-1.52	0.00	0.005	Putative_uncharacterized_protein	AT3G56480
TC02G031860	-0.61	-1.52	0.00	0.016	Putative_Fructokinase-2	NA
TC10G015880	-0.62	-1.53	0.00	0.002	Annexin_D3	AT2G38760
TC02G027730	-0.62	-1.54	0.00	0.006	Myosin_heavy_chain,_clone,_putative	AT1G14840
TC00G035560	-0.62	-1.54	0.00	0.005	Fasciclin-like_arabinogalactan_protein_17	AT5G06390
TC08G003440	-0.62	-1.54	0.00	0.014	Endoglucanase_2	AT1G19940
TC04G019850	-0.63	-1.55	0.00	0.012	Putative_uncharacterized_protein	AT2G43490
TC03G015870	-0.63	-1.55	0.00	0.001	Putative_Copine-3	AT5G07300
TC04G005560	-0.63	-1.55	0.01	0.041	Putative_uncharacterized_protein	AT1G52140

TC04G025850	-0.64	-1.55	0.00	0.007	Predicted_protein	AT3G23410
TC06G016980	-0.64	-1.56	0.00	0.004	Sugar_carrier_protein_C	AT1G11260
TC04G010210	-0.64	-1.56	0.00	0.000	Probable_UDP-glucose_6-dehydrogenase_1	AT3G29360
TC09G012250	-0.64	-1.56	0.00	0.005	Pyrophosphate-energized_vacuolar_membrane_proton_pump	AT1G15690
TC02G010330	-0.65	-1.56	0.00	0.011	NA	NA
TC08G006650	-0.66	-1.58	0.00	0.004	Tubulin_alpha-1_chain	AT1G50010
TC02G030420	-0.66	-1.58	0.00	0.001	NA	NA
TC09G033550	-0.66	-1.59	0.00	0.036	NA	NA
TC03G027830	-0.67	-1.59	0.00	0.032	Homeobox_protein_BEL1_homolog	AT5G41410
TC01G031600	-0.68	-1.60	0.00	0.006	NA	NA
TC08G001240	-0.68	-1.61	0.00	0.001	Molybdopterin_cofactor_sulfurase,_putative	AT2G23520
TC03G025890	-0.68	-1.61	0.00	0.025	NA	NA
TC09G012350	-0.69	-1.61	0.00	0.000	Heparanase-like_protein_3	AT5G34940
TC07G016420	-0.69	-1.62	0.00	0.023	Hypothetical_protein	NA
TC02G030290	-0.71	-1.64	0.01	0.048	Inositol_oxygenase_1	AT1G14520
TC09G028950	-0.73	-1.66	0.00	0.003	Glucomannan_4-beta-mannosyltransferase_2	AT5G22740
TC06G003360	-0.74	-1.67	0.00	0.000	Putative_Protein_SRG1	AT1G49390
TC05G004310	-0.74	-1.67	0.00	0.000	NA	NA
TC02G017900	-0.74	-1.68	0.00	0.026	NA	NA
TC02G006210	-0.75	-1.69	0.00	0.013	Putative_Indole-3-acetic_acid-induced_protein_ARG7	AT5G10990
TC10G000830	-0.76	-1.69	0.00	0.024	Laccase-12	AT5G05390
TC04G001330	-0.76	-1.69	0.00	0.007	NA	NA
TC06G018680	-0.77	-1.70	0.00	0.002	NA	NA
TC06G014050	-0.78	-1.72	0.00	0.020	NA	NA
TC02G024390	-0.78	-1.72	0.00	0.019	Putative_carboxyl-terminal_proteinase	AT1G70550
TC01G002190	-0.78	-1.72	0.00	0.000	Cytochrome_P450_84A1	AT4G36220
TC10G015870	-0.79	-1.73	0.00	0.013	Annexin_D4	AT2G38750
TC10G001410	-0.80	-1.74	0.00	0.002	Putative_uncharacterized_protein	AT2G29970
TC00G009620	-0.80	-1.74	0.00	0.000	Putative_uncharacterized_protein	AT5G15890
TC05G028950	-0.80	-1.74	0.01	0.047	NA	NA
TC01G001190	-0.82	-1.77	0.00	0.015	Peroxidase_64	AT5G42180
TC07G014620	-0.84	-1.79	0.00	0.004	Putative_Probable_gibberellin_receptor_GID1L3	AT5G62180
TC06G017140	-0.84	-1.79	0.00	0.005	ATP_binding_protein,_putative	AT4G05150
TC06G002530	-0.89	-1.85	0.00	0.024	Predicted_protein	AT5G52060
TC01G018820	-0.95	-1.93	0.00	0.002	Glucose-6-phosphate_1-dehydrogenase_1,_chloroplastic	AT5G35790
TC01G031530	-1.01	-2.02	0.00	0.024	NA	NA
TC02G005170	-1.02	-2.02	0.00	0.005	Predicted_protein	AT2G16660

TC02G012170	-1.10	-2.15	0.00	0.020	Beta-xylosidase/alpha-L-arabinofuranosidase_2	AT5G64570
TC08G009960	-1.12	-2.17	0.00	0.001	Nitrate_reductase_[NADH]	AT1G77760
TC09G026710	-1.15	-2.21	0.00	0.006	Putative_Probable_peptide_transporter_At1g52190	AT3G45680
TC05G004360	-1.16	-2.24	0.00	0.026	NA	NA
TC02G023300	-1.50	-2.83	0.00	0.020	NA	NA

[a] Mean differences and p values were calculated by fitting a general linear model in which the SA to H_2O differences (within leaf type) were modeled against *overall_mean* + *leaf type + error,* then testing if *overall_mean* was significantly different than zero. Fold change columns show transformed and untransformed values of *overall_mean.* A BH multiple testing adjustment was used for determination of significance.

Supplemental Table 3 - Genes with statistically significant differential regulation in both genotypes

TC ID	Sca6 Log_2 Fold Change SA-H_2O[a]	ICS1 Log_2 Fold Change SA-H_2O[a]	AThal BestHitID	Arabidopsis Annotation
TC04G003810	2.73	-0.58	ATCG00680	Photosystem II CP47 chlorophyll apoprotein
TC06G001520	1.38	1.43	NA	NA
TC09G031960	1.28	0.54	AT3G12580	Heat shock cognate 70 kDa protein
TC01G031600	1.23	-0.68	NA	NA
TC04G003840	1.15	-0.56	ATCG00540	Apocytochrome f
TC06G015310	1.11	1.05	AT2G20560	Putative DnaJ homolog subfamily B member 4
TC00G089300	1.09	1.08	AT1G74310	Heat shock protein 101
TC05G003590	1.09	1.70	AT5G02020	Putative uncharacterized protein
TC09G012660	0.98	0.86	NA	NA
TC03G022890	0.98	1.55	AT1G64660	Putative Methionine gamma-lyase
TC03G016040	0.97	1.23	AT5G61820	Predicted protein
TC04G002800	0.88	0.83	NA	NA
TC01G033460	0.88	0.86	AT1G33060	Hypothetical protein
TC10G003040	0.86	0.75	AT3G55580	Putative Probable E3 ubiquitin-protein ligase HERC2
TC03G027270	0.84	1.01	AT3G48990	Putative peroxisomal-coenzyme A synthetase
TC07G007410	0.83	0.69	AT5G54470	Transcription factor, putative
TC04G026780	0.81	0.88	AT3G23920	Beta-amylase 1, chloroplastic
TC00G061020	0.81	0.46	NA	Putative uncharacterized protein
TC10G016440	0.80	0.90	NA	NA
TC06G012620	0.80	0.77	AT1G30070	Putative Calcyclin-binding protein
TC00G019560	0.77	-0.60	AT1G15420	Predicted protein (Fragment)

TC03G013700	0.76	0.84	AT3G48280	Cytochrome P450 71A25
TC05G003840	0.75	1.37	AT2G39800	Delta-1-pyrroline-5-carboxylate synthase
TC03G006290	0.73	0.92	AT5G17300	Predicted protein
TC05G006130	0.73	0.80	AT2G42760	Putative uncharacterized protein
TC03G030500	0.72	1.04	AT1G72030	Putative uncharacterized protein
TC08G011870	0.72	1.50	AT1G21400	2-oxoisovalerate dehydrogenase subunit alpha, mitochondrial
TC01G019270	0.71	1.77	AT2G04240	Putative uncharacterized protein
TC04G024360	0.71	0.62	AT4G14690	Early light-induced protein, chloroplastic
TC05G020320	0.70	0.45	AT3G13570	Serine/arginine rich splicing factor, putative
TC01G020510	0.68	0.63	AT1G05680	Uncharacterized UDP-glucosyltransferase At1g05670
TC01G022340	0.67	0.52	AT3G07090	Putative PPPDE peptidase domain-containing protein 2
TC01G017530	0.65	-0.43	NA	NA
TC05G015130	0.65	0.74	AT2G41250	N-acylneuraminate-9-phosphatase, putative
TC03G019930	0.64	0.81	AT5G52570	Beta-carotene hydroxylase
TC03G015550	0.64	0.37	AT3G26890	Putative uncharacterized protein
TC09G035070	0.64	0.95	AT3G51630	Probable serine/threonine-protein kinase WNK4
TC00G036580	0.61	0.57	AT3G28480	Prolyl 4-hydroxylase alpha subunit, putative
TC10G007380	0.61	0.33	NA	Putative RING finger protein B
TC03G022970	0.61	0.68	AT5G53550	Metal-nicotianamine transporter YSL1
TC08G002840	0.60	0.46	NA	NA
TC09G003520	0.59	-0.45	AT5G11490	Predicted protein
TC00G092080	0.59	0.65	NA	Putative uncharacterized protein
TC05G005640	0.58	-0.34	AT5G02770	Putative SAP domain-containing ribonucleoprotein
TC01G029220	0.57	1.14	AT3G63010	Probable gibberellin receptor GID1L2
TC08G004140	0.56	0.72	AT1G60420	Putative Nucleoredoxin
TC09G026080	0.55	0.80	AT1G51340	Aluminum-activated citrate transporter
TC03G021650	0.55	0.35	AT3G49390	RNA-binding protein, putative
TC00G013030	0.53	0.37	NA	Putative uncharacterized protein
TC05G006380	0.52	1.00	AT2G37170	Aquaporin PIP2-2
TC01G030930	0.52	-0.35	AT4G02260	Predicted protein
TC08G013850	0.51	-0.30	AT1G33680	Putative Far upstream element-binding protein 3
TC01G001330	0.51	-0.37	AT2G17790	Putative Vacuolar protein sorting-associated protein 35
TC07G000190	0.50	-0.36	NA	Probable WRKY transcription factor 2
TC09G001560	0.49	-0.45	AT4G31880	Nucleic acid binding protein, putative
TC02G014240	0.49	0.69	AT3G21890	Predicted protein
TC10G009100	0.49	-0.36	NA	Putative uncharacterized protein
TC02G034460	0.49	0.25	AT1G68660	ATP-dependent Clp protease adapter protein clpS
TC01G037720	0.48	0.51	AT2G45380	Predicted protein
TC08G008390	0.48	0.49	NA	Hypothetical protein

TC02G020210	0.47	0.50	AT1G67360	REF/SRPP-like protein At1g67360
TC04G015180	0.47	0.58	AT2G41430	ERD15
TC00G081420	0.44	0.43	AT2G41870	Remorin, putative
TC02G000970	0.44	-0.33	AT4G05420	DNA damage-binding protein 1
TC02G027730	0.44	-0.62	AT1G14840	Myosin heavy chain, clone, putative
TC01G002780	0.43	0.37	AT5G66450	Putative uncharacterized protein
TC01G040610	0.43	0.27	AT3G63520	Carotenoid 9,10(9',10')-cleavage dioxygenase 1
TC08G002030	0.43	0.31	AT1G75180	Predicted protein
TC03G018200	0.42	0.43	AT1G12240	Beta-fructofuranosidase, soluble isoenzyme I
TC06G003340	0.42	-0.45	AT1G15690	Pyrophosphate-energized vacuolar membrane proton pump
TC02G010520	0.41	0.43	AT2G23970	Putative glutamine amidotransferase YLR126C
TC07G014720	0.39	-0.33	AT1G79090	Predicted protein
TC07G017290	0.38	-0.23	AT1G79440	Succinate-semialdehyde dehydrogenase, mitochondrial
TC09G033490	0.38	-0.32	AT1G07990	Putative uncharacterized protein
TC09G022300	0.36	-0.36	AT3G45140	Lipoxygenase 2, chloroplastic
TC08G001240	0.36	-0.68	AT2G23520	Molybdopterin cofactor sulfurase, putative
TC00G033720	0.35	0.20	AT1G53280	Putative Protein thiJ
TC02G028560	0.35	0.36	AT1G70790	Putative Probable ADP-ribosylation factor GTPase-activating protein AGD11
TC10G011790	0.35	0.26	AT3G44110	DnaJ protein homolog
TC09G032890	0.34	0.30	AT3G45780	Phototropin-1
TC02G012690	0.34	-0.49	NA	NA
TC00G089630	0.34	-0.40	AT5G62670	ATPase 11, plasma membrane-type
TC04G026120	0.33	0.52	AT5G13890	Putative uncharacterized protein
TC05G031960	0.33	-0.39	NA	NA
TC09G013770	0.32	0.36	AT3G18830	Polyol transporter 5
TC03G020770	0.31	0.38	AT5G52230	Putative uncharacterized protein
TC01G018610	0.31	0.29	AT2G05160	Zinc finger CCCH domain-containing protein 18
TC01G040090	0.31	-0.27	AT3G63460	Predicted protein
TC02G011710	0.31	0.20	AT5G64200	Serine/arginine rich splicing factor, putative
TC01G003540	0.31	0.34	AT4G16580	Probable protein phosphatase 2C 80
TC09G002870	0.31	0.31	AT4G30960	CBL-interacting serine/threonine-protein kinase 6
TC03G021030	0.30	0.35	AT5G53000	PP2A regulatory subunit TAP46
TC01G020450	0.30	-0.22	AT5G48385	Putative uncharacterized protein
TC09G004540	0.30	0.36	AT4G32930	Putative UPF0587 protein C1orf123 homolog
TC04G020340	0.29	0.32	AT4G14030	Putative selenium-binding protein
TC03G030630	0.29	-0.31	AT4G19110	Uncharacterized protein At4g19112.1
TC01G038370	0.29	-0.23	AT4G00050	Transcription factor UNE10
TC08G011990	0.28	0.47	AT1G21410	F-box/LRR-repeat protein 5
TC05G031720	0.28	-0.17	AT3G05830	Predicted protein

TC04G030560	0.27	0.22	AT3G06483	Putative [Pyruvate dehydrogenase [lipoamide]] kinase, mitochondrial
TC01G037650	0.27	0.21	AT2G45400	Putative Dihydroflavonol-4-reductase
TC01G021990	0.27	0.37	AT3G25120	Predicted protein
TC02G029750	0.27	-0.40	AT1G14720	Probable xyloglucan endotransglucosylase/hydrolase protein 28
TC06G019520	0.26	0.13	AT1G11910	Aspartic proteinase
TC09G002670	0.26	-0.29	AT2G23980	Probable cyclic nucleotide-gated ion channel 5
TC00G056860	0.26	-0.25	AT1G25440	Putative Zinc finger protein CONSTANS-LIKE 16
TC03G017290	0.25	-0.22	AT5G06160	Putative Splicing factor 3A subunit 3
TC08G008900	0.25	-0.37	AT1G78240	Probable pectin methyltransferase QUA2
TC02G002990	0.25	-0.17	AT5G24690	Predicted protein
TC00G029800	0.25	-0.15	AT3G58500	Serine/threonine-protein phosphatase PP2A catalytic subunit
TC04G030270	0.25	-0.32	AT1G48110	Yth domain-containing protein, putative
TC03G027640	0.24	-0.20	AT1G63770	Putative Aminopeptidase N
TC04G022640	0.23	-0.18	AT4G14290	Catalytic, putative
TC03G025700	0.23	0.24	AT4G22670	Putative Hsc70-interacting protein
TC02G034690	0.23	-0.35	AT3G25690	Protein CHUP1, chloroplastic
TC01G003290	0.22	-0.28	AT5G66770	Scarecrow-like protein 4
TC03G002290	0.21	-0.37	AT4G20360	Elongation factor Tu, chloroplastic
TC09G010660	0.21	-0.17	AT1G15470	Serine-threonine kinase receptor-associated protein
TC00G022300	0.20	-0.31	AT2G07360	Putative uncharacterized protein
TC00G059940	0.19	0.15	AT5G40810	Cytochrome c1-1, heme protein, mitochondrial
TC09G029990	0.18	0.18	NA	NA
TC08G005720	0.18	-0.19	AT4G24620	Glucose-6-phosphate isomerase
TC04G014440	0.16	0.17	AT5G16150	Plastidic glucose transporter 4
TC04G013930	0.15	-0.30	AT3G18270	Putative L-Ala-D/L-Glu epimerase
TC05G005830	0.13	0.18	NA	Predicted protein
TC01G026730	0.13	-0.21	AT4G16430	Transcription factor bHLH3
TC00G080870	-0.11	-0.12	AT5G01010	Putative uncharacterized protein
TC04G002630	-0.15	-0.13	AT5G14310	Putative uncharacterized protein
TC08G010320	-0.16	0.19	AT4G08240	Predicted protein
TC05G018850	-0.16	-0.15	AT3G12130	Zinc finger CCCH domain-containing protein 14
TC10G001890	-0.17	-0.15	AT5G05760	Syntaxin-31
TC09G006690	-0.17	-0.16	AT3G14080	Lsm1, putative
TC09G001360	-0.18	-0.16	AT4G31860	Probable protein phosphatase 2C 60
TC09G008410	-0.18	-0.22	AT1G70980	Asparaginyl-tRNA synthetase, cytoplasmic 1
TC09G003510	-0.18	-0.32	AT5G11480	GTP-binding protein At2g22870
TC03G029930	-0.19	-0.24	AT2G35500	Shikimate kinase-like protein
TC05G006520	-0.20	-0.35	AT2G37220	Ribonucleoprotein At2g37220, chloroplastic
TC01G010180	-0.20	-0.21	AT4G39490	Putative Cytochrome P450 86B1

TC02G034470	-0.20	-0.27	NA	NA
TC02G029760	-0.21	0.31	NA	NA
TC05G002500	-0.21	-0.17	AT2G38180	GDSL esterase/lipase CPRD49
TC01G028010	-0.21	-0.16	AT1G03110	WD-repeat protein, putative
TC01G003740	-0.22	-0.28	AT4G36810	Geranylgeranyl pyrophosphate synthase, chloroplastic
TC00G049090	-0.22	-0.25	AT1G52670	Biotin/lipoyl attachment
TC06G006840	-0.23	-0.45	AT1G15500	Plastidic ATP/ADP-transporter
TC05G022350	-0.23	-0.29	AT3G03050	Cellulose synthase-like protein D3
TC02G024400	-0.23	-0.39	NA	NA
TC02G009410	-0.24	-0.29	AT4G34200	D-3-phosphoglycerate dehydrogenase, chloroplastic
TC06G018700	-0.24	-0.20	AT4G21170	Putative Pentatricopeptide repeat-containing protein At4g21170
TC01G033350	-0.24	-0.16	AT1G02280	Translocase of chloroplast 33, chloroplastic
TC07G012860	-0.24	-0.20	AT1G61730	Transcription regulator, putative
TC06G012100	-0.24	0.39	AT1G30330	Auxin response factor 6
TC03G001670	-0.25	-0.22	AT5G63050	Putative uncharacterized protein
TC03G020090	-0.26	-0.26	AT4G25630	Putative Fibrillarin
TC10G005060	-0.27	-0.26	AT5G60390	Elongation factor 1-alpha
TC08G003640	-0.27	-0.32	AT1G75780	Tubulin beta-6 chain
TC01G025590	-0.27	-0.21	AT2G20420	Succinyl-CoA ligase [GDP-forming] subunit beta, mitochondrial
TC05G000450	-0.27	0.22	AT3G44370	Putative ALBINO3-like protein 2, chloroplastic
TC10G002550	-0.28	-0.30	AT3G55630	Putative Folylpolyglutamate synthase, mitochondrial
TC09G022160	-0.28	-0.28	AT3G57930	Predicted protein
TC01G011220	-0.29	-0.42	AT3G18680	Uridylate kinase
TC02G020400	-0.29	-0.12	AT3G56820	Putative uncharacterized protein
TC00G016810	-0.29	0.31	NA	Ring finger protein, putative
TC01G007950	-0.29	-0.42	AT4G38050	Nucleobase-ascorbate transporter 11
TC01G008500	-0.30	-0.43	AT5G65280	Putative LanC-like protein 2
TC05G031090	-0.30	-0.24	AT1G56350	Putative Peptide chain release factor 2
TC09G027220	-0.30	0.27	AT1G08480	Putative uncharacterized protein
TC02G000990	-0.30	-0.39	AT2G34660	ABC transporter C family member 2
TC07G008920	-0.30	-0.27	NA	NA
TC05G004950	-0.30	-0.21	AT2G37540	Putative Retinol dehydrogenase 12
TC04G013830	-0.30	-0.26	AT3G02600	Putative lipid phosphate phosphatase 3, chloroplastic
TC09G027090	-0.31	-0.29	AT5G20950	Putative Lysosomal beta glucosidase
TC04G010210	-0.31	-0.64	AT3G29360	Probable UDP-glucose 6-dehydrogenase 1
TC00G014640	-0.31	0.27	AT5G48840	Pantoate--beta-alanine ligase
TC08G012990	-0.32	-0.36	AT1G76760	Thioredoxin-like protein slr0233
TC07G010830	-0.32	-0.28	AT1G78630	50S ribosomal protein L13, chloroplastic
TC02G016150	-0.32	-0.35	AT4G28510	Prohibitin-2

TC01G028890	-0.33	-0.41	AT4G02930	Elongation factor Tu, mitochondrial
TC04G015810	-0.33	-0.31	AT3G08600	Putative uncharacterized protein
TC03G029950	-0.33	-0.33	NA	Predicted protein
TC05G023650	-0.33	-0.37	AT3G03770	Putative Probable LRR receptor-like serine/threonine-protein kinase At1g14390
TC09G014740	-0.33	0.30	NA	Hypothetical protein
TC01G035210	-0.33	-0.25	AT2G46170	Reticulon-like protein B5
TC09G011790	-0.33	-0.40	NA	Predicted protein
TC01G039950	-0.34	-0.38	AT2G44640	Putative uncharacterized protein
TC08G003530	-0.34	-0.28	NA	NA
TC02G012120	-0.34	0.43	NA	LL-diaminopimelate aminotransferase, chloroplastic
TC00G027040	-0.35	-0.37	AT1G29470	Probable methyltransferase PMT25
TC03G019700	-0.35	-0.28	AT4G25500	Arginine/serine-rich-splicing factor RSP40
TC09G014300	-0.36	-0.26	AT1G17890	Putative GDP-L-fucose synthase 2
TC03G016690	-0.36	-0.23	AT5G51220	Putative uncharacterized protein
TC07G004790	-0.38	-0.57	AT5G54770	Thiazole biosynthetic enzyme, chloroplastic
TC05G019880	-0.38	-0.28	AT2G43945	Predicted protein (Fragment)
TC01G010280	-0.39	-0.16	AT3G57940	UPF0202 protein At1g10490
TC01G037950	-0.40	-0.45	AT3G60690	Putative Auxin-induced protein X10A
TC01G002060	-0.40	-0.32	AT1G15380	Putative Lactoylglutathione lyase
TC10G015880	-0.41	-0.62	AT2G38760	Annexin D3
TC03G002170	-0.42	-0.47	AT1G23280	Putative Protein MAK16 homolog B
TC06G018680	-0.42	-0.77	NA	NA
TC03G002320	-0.43	-0.27	AT3G18580	Predicted protein
TC05G028430	-0.45	-0.45	AT4G13180	Short-chain type dehydrogenase/reductase
TC09G015240	-0.45	-0.40	AT4G30440	UDP-glucuronate 4-epimerase 1
TC05G020310	-0.45	-0.23	AT3G11964	Putative Protein RRP5 homolog
TC04G012070	-0.46	-0.34	AT5G15700	DNA-directed RNA polymerase 2, chloroplastic/mitochondrial
TC04G002710	-0.46	-0.32	AT3G27280	Prohibitin-1, mitochondrial
TC00G020080	-0.48	-0.49	AT5G07010	Putative Sulfotransferase 16
TC00G014550	-0.48	-0.51	AT5G61960	Predicted protein
TC08G011180	-0.49	-0.50	NA	NA
TC00G051081	-0.52	0.49	NA	NA
TC01G034580	-0.52	-0.38	AT5G63630	DEAD-box ATP-dependent RNA helicase 26
TC09G031410	-0.52	-0.40	AT5G59500	Predicted protein
TC03G023410	-0.52	-0.37	NA	NA
TC01G000260	-0.54	-0.59	NA	NA
TC10G012980	-0.54	-0.43	AT3G12390	Putative uncharacterized protein
TC00G020830	-0.55	-0.80	AT5G57490	Putative Porin%2C eukaryotic type
TC02G033190	-0.55	-0.27	AT1G69580	Transcription factor, putative

TC05G013900	-0.55	0.48	AT5G06150	G2/mitotic-specific cyclin S13-6
TC02G006970	-0.59	-0.54	AT2G21660	Hypothetical protein
TC03G014200	-0.60	-0.50	AT5G63290	Putative Oxygen-independent coproporphyrinogen-III oxidase-like protein sll1917
TC10G014000	-0.63	-0.32	AT3G12270	Probable protein arginine N-methyltransferase 3
TC03G026830	-0.64	-0.60	AT4G23060	Putative uncharacterized protein
TC03G028600	-0.65	0.62	NA	NA
TC04G022740	-0.68	-0.58	AT3G23580	Ribonucleoside-diphosphate reductase small chain A
TC03G029820	-0.69	-0.51	AT4G23620	50S ribosomal protein
TC06G016980	-0.73	-0.64	AT1G11260	Sugar carrier protein C
TC03G025560	-0.73	-0.44	AT1G62730	Putative UPF0551 protein C8orf38 homolog, mitochondrial
TC09G026710	-0.73	-1.15	AT3G45680	Putative Probable peptide transporter At1g52190
TC07G016420	-0.74	-0.69	NA	Hypothetical protein
TC07G002090	-0.77	0.60	NA	NA
TC04G001330	-0.82	-0.76	NA	NA
TC03G027850	-0.83	-0.45	NA	NA
TC05G028950	-0.87	-0.80	NA	NA
TC10G001550	-1.03	-0.55	AT2G40080	Putative uncharacterized protein
TC00G017110	-2.73	0.58	AT4G03420	Hypothetical protein

[a] Expression differences taken from Supplemental Tables 1 and 2

Supplemental Table 4 - PR Genes measured by microarray

PR Class	E-value	TC ID	Arabidopsis Annotation	Combined Genotypes Log_2 Fold change[a]	Combined Genotypes B-H Corrected p-value[a]	Sca6 Log_2 Fold Change[b]	Sca6 B-H Corrected p-value[b]	ICS1 Log_2 Fold Change[b]	ICS1 B-H Corrected p-value[b]
PR1	3.00E-38	TC09G016590	Pathogenesis-related_protein_PR-1	0.05	0.834	-0.13	0.681	0.22	0.433
PR1	2.00E-35	TC09G016580	STS14_protein,_putative	0.75	0.007	1.00	0.010	0.49	0.127
PR1	1.00E-26	TC01G003940	STS14_protein,_putative	0.10	0.645	-0.57	0.073	0.77	0.023
PR2	7.00E-102	TC09G024130	Lichenase	0.54	0.000	-0.03	0.922	1.11	0.000
PR2	4.00E-97	TC04G029300	Glucan_endo-1,3-beta-glucosidase,_basic_vacuolar_isoform	1.71	0.001	0.73	0.200	2.70	0.001
PR2	4.00E-63	TC09G008700	Glucan_endo-1,3-beta-glucosidase_13	0.11	0.435	0.10	0.614	0.11	0.590
PR2	3.00E-61	TC09G011610	Putative_Glucan_endo-1,3-beta-glucosidase_11	-0.33	0.013	-0.09	0.611	-0.57	0.006
PR2	6.00E-55	TC01G017650	Putative_Glucan_endo-1,3-beta-glucosidase_3	0.29	0.452	0.01	0.984	0.56	0.299
PR2	2.00E-54	TC08G001980	Glucan_endo-1,3-beta-glucosidase_7	0.03	0.850	-0.06	0.828	0.13	0.583
PR2	2.00E-53	TC03G022650	Glucan_endo-1,3-beta-glucosidase_11	-0.24	0.183	-0.28	0.193	-0.20	0.344
PR2	2.00E-52	TC09G031660	Glucan_endo-1,3-beta-glucosidase	0.19	0.410	-0.11	0.773	0.48	0.146
PR2	3.00E-52	TC07G002650	Putative_Glucan_endo-1,3-beta-glucosidase_12	-0.22	0.074	-0.29	0.099	-0.14	0.398
PR2	6.00E-47	TC08G005240	NA	-0.17	0.661	-0.29	0.614	-0.05	0.931
PR2	2.00E-45	TC05G028370	Glucan_endo-1,3-beta-glucosidase_8	0.07	0.280	0.03	0.789	0.11	0.236
PR2	1.00E-40	TC01G014030	Glucan_endo-1,3-beta-glucosidase_5	0.06	0.639	0.03	0.881	0.09	0.621
PR2	6.00E-07	TC01G006420	NA	-0.30	0.326	-0.12	0.828	-0.49	0.268
PR3	3.00E-79	TC01G000770	Endochitinase	0.65	0.140	-0.13	0.864	1.44	0.040
PR3	6.00E-28	TC04G029180	NA	-0.26	0.209	-0.18	0.570	-0.34	0.253
PR3	7.00E-24	TC04G018110	Endochitinase_PR4	0.27	0.362	-0.34	0.455	0.88	0.056
PR3	3.00E-73	TC02G003890	Endochitinase_1	1.56	0.010	0.93	0.212	2.18	0.012
PR4	7.00E-26	TC05G027250	Pathogenesis-related_protein_P2	0.40	0.272	0.27	0.642	0.53	0.307
PR5	7.00E-68	TC02G005190	NA	-0.04	0.919	-0.25	0.634	0.17	0.744
PR6	2.00E-07	TC05G022770	Inhibitor_of_trypsin_and_hageman_factor	0.12	0.500	-0.07	0.821	0.30	0.221
PR7	6.00E-142	TC01G037030	NA	0.29	0.443	0.59	0.280	-0.02	0.970
PR7	9.00E-137	TC02G005130	Subtilisin-like_protease	-0.10	0.236	0.01	0.939	-0.21	0.070
PR7	1.00E-136	TC01G006160	Subtilisin-like_protease	-0.05	0.515	-0.02	0.896	-0.09	0.445
PR7	4.00E-134	TC01G037010	NA	0.16	0.603	-0.11	0.836	0.42	0.309
PR7	8.00E-123	TC06G010630	Subtilisin-like_protease	-0.04	0.816	-0.20	0.382	0.12	0.587
PR7	4.00E-119	TC08G000270	Putative_Subtilisin-like_protease	-0.19	0.209	-0.26	0.251	-0.12	0.589
PR7	3.00E-117	TC03G022570	Putative_Subtilisin-like_protease	-0.06	0.819	0.08	0.855	-0.21	0.569
PR7	4.00E-117	TC06G013520	NA	-0.20	0.164	-0.08	0.730	-0.32	0.129

PR7	8.00E-116	TC00G032610	Putative_Subtilisin-like_protease	0.08	0.792	0.92	0.035	-0.77	0.062
PR7	1.00E-106	TC08G000260	Putative_Subtilisin-like_protease	-0.08	0.540	-0.10	0.624	-0.06	0.751
PR7	7.00E-70	TC00G038560	Putative_Subtilisin-like_protease	0.11	0.233	0.20	0.112	0.03	0.857
PR8	2.00E-91	TC01G035050	Acidic_endochitinase	0.75	0.014	-0.01	0.981	1.50	0.003
PR8	8.00E-77	TC01G032120	NA	0.52	0.229	0.11	0.885	0.94	0.145
PR9	2.00E-78	TC10G016080	NA	-0.23	0.270	-0.17	0.621	-0.29	0.334
PR9	5.00E-78	TC09G034930	Peroxidase_4	1.14	0.041	0.12	0.893	2.15	0.015
PR9	9.00E-78	TC10G015970	Peroxidase_4	0.20	0.404	-0.29	0.430	0.69	0.062
PR9	5.00E-67	TC02G030420	NA	-0.53	0.000	-0.40	0.066	-0.66	0.001
PR9	2.00E-66	TC08G013300	Peroxidase_12	0.57	0.010	0.29	0.293	0.86	0.009
PR9	4.00E-63	TC01G008530	Peroxidase_17	0.07	0.776	0.00	0.997	0.14	0.672
PR9	1.00E-60	TC01G013330	Peroxidase_47	-0.04	0.889	-0.26	0.488	0.18	0.619
PR9	1.00E-60	TC08G004060	Peroxidase_64	-0.64	0.026	-0.58	0.137	-0.70	0.072
PR9	2.00E-60	TC01G001190	Peroxidase_64	-0.64	0.008	-0.46	0.123	-0.82	0.015
PR9	1.00E-53	TC08G012700	Peroxidase_43	0.58	0.029	0.20	0.575	0.95	0.018
PR9	2.00E-53	TC01G006280	Peroxidase_73	0.11	0.161	-0.12	0.324	0.34	0.014
PR9	3.00E-29	TC04G016710	NA	-0.18	0.164	0.03	0.888	-0.38	0.051
PR9	1.00E-100	TC00G014230	Peroxidase_15	0.19	0.145	0.00	0.993	0.37	0.058
PR10	1.00E-31	TC01G031100	Pathogenesis-related_protein_STH-2	0.24	0.575	-0.39	0.531	0.86	0.150
PR10	1.00E-25	TC04G028750	Putative_Major_allergen_Pru_av_1	0.55	0.012	0.06	0.856	1.03	0.003
PR14	3.00E-15	TC04G016380	NA	0.45	0.236	-0.17	0.799	1.08	0.067
PR14	4.00E-15	TC04G016440	NA	1.56	0.003	1.07	0.074	2.04	0.005
PR14	6.00E-12	TC04G016450	Non-specific_lipid-transfer_protein	-0.08	0.620	-0.18	0.450	0.02	0.950
PR16	2.00E-45	TC01G033170	Germin-like_protein_subfamily_2_member_4	-0.49	0.023	-0.42	0.142	-0.56	0.058

[a] Mean differences and p values were calculated by fitting a general linear model in which the SA to H_2O differences (within cultivar and leaf type) were modeled against *overall_mean* + *cultivar* + *leaf-type* + *cultivar*leaf-type_interaction* + *error*, then testing if *overall_mean* was significantly different than zero. Column shows values of *overall_mean.* A BH multiple testing adjustment was used.

[b] Mean differences and p values were calculated by fitting a general linear model in which the SA to H_2O differences (within leaf type) were modeled against *overall_mean* + *leaf _type* + *error*, then testing if the overall mean was significantly different than zero. Columns show values of *overall_mean.* A BH multiple testing adjustment was used.

Supplemental Table 5 - Parametric Analysis of Gene Expression - Enriched GO terms after SA treatment for Sca6 Genotype

No	GO Term	Ontology[a]	Description	Z-score	Mean Log_2 FC	BY-adjusted p-value
1	GO:0006091	BP	generation of precursor metabolites and energy	7.8	1	1.60E-12
2	GO:0015979	BP	photosynthesis	7	1.1	2.50E-10
3	GO:0009408	BP	response to heat	4	0.67	0.0022
4	GO:0009266	BP	response to temperature stimulus	3.6	0.49	0.0078
5	GO:0009628	BP	response to abiotic stimulus	3.6	0.36	0.0078
6	GO:0006979	BP	response to oxidative stress	3.3	0.65	0.018
7	GO:0009416	BP	response to light stimulus	3.3	0.46	0.019
8	GO:0006950	BP	response to stress	3.2	0.33	0.025
9	GO:0009314	BP	response to radiation	3.1	0.44	0.03
10	GO:0042254	BP	ribosome biogenesis	-4	-0.32	0.0022
11	GO:0022613	BP	ribonucleoprotein complex biogenesis	-4	-0.32	0.0022
12	GO:0006412	BP	translation	-4.3	-0.13	0.001
13	GO:0005198	MF	structural molecule activity	-3.6	-0.1	0.01
14	GO:0003735	CC	structural constituent of ribosome	-3.7	-0.12	0.01
15	GO:0034357	CC	photosynthetic membrane	5.2	0.88	5.20E-06
16	GO:0009579	CC	thylakoid	4.7	0.73	3.40E-05
17	GO:0070469	CC	respiratory chain	3.6	0.68	0.0022
18	GO:0044435	CC	plastid part	3.4	0.4	0.0047
19	GO:0043234	CC	protein complex	3.2	0.35	0.0071
20	GO:0044425	CC	membrane part	3.2	0.39	0.0071
21	GO:0044436	CC	thylakoid part	2.9	0.59	0.016
22	GO:0044434	CC	chloroplast part	2.9	0.38	0.018
23	GO:0009507	CC	chloroplast	2.6	0.27	0.033
24	GO:0005829	CC	cytosol	-2.6	-0.02	0.033
25	GO:0031981	CC	nuclear lumen	-2.6	-0.12	0.033
26	GO:0015934	CC	large ribosomal subunit	-2.6	-0.16	0.031
27	GO:0033279	CC	ribosomal subunit	-2.7	-0.067	0.026
28	GO:0044428	CC	nuclear part	-2.7	-0.11	0.024
29	GO:0005730	CC	nucleolus	-3.3	-0.24	0.0061
30	GO:0022625	CC	cytosolic large ribosomal subunit	-3.5	-0.34	0.0028
31	GO:0005840	CC	ribosome	-3.9	-0.12	0.00091
32	GO:0022627	CC	cytosolic small ribosomal subunit	-4	-0.32	0.00064
33	GO:0030529	CC	ribonucleoprotein complex	-4	-0.12	0.00064
34	GO:0043232	CC	intracellular non-membrane-bounded organelle	-4.7	-0.12	3.40E-05
35	GO:0043228	CC	non-membrane-bounded organelle	-4.7	-0.12	3.40E-05
36	GO:0044445	CC	cytosolic part	-5.2	-0.31	5.20E-06
37	GO:0022626	CC	cytosolic ribosome	-5.4	-0.31	3.80E-06

[a] BP = Biological Process; MF = Molecular Function; CC = Cellular Component.

Supplemental Table 6 - Parametric Analysis of Gene Expression - Enriched GO terms after SA treatment for ICS1 Genotype

No	GO Term	Ontology[a]	Description	Z-score	Mean Log_2 FC	BY-adjusted p-value
1	GO:0006979	BP	response to oxidative stress	4.5	0.58	0.0015
2	GO:0042221	BP	response to chemical stimulus	3.7	0.2	0.018
3	GO:0007154	BP	cell communication	3.5	0.53	0.025
4	GO:0005506	MF	iron ion binding	3.7	0.57	0.018
5	GO:0030312	CC	external encapsulating structure	2.5	0.27	0.033
6	GO:0005618	CC	cell wall	2.5	0.27	0.033
7	GO:0005576	CC	extracellular region	2.4	0.36	0.036
8	GO:0048046	CC	apoplast	2.4	0.39	0.039
9	GO:0044436	CC	thylakoid part	-2.3	-0.26	0.041
10	GO:0009526	CC	plastid envelope	-2.4	-0.22	0.039
11	GO:0034357	CC	photosynthetic membrane	-2.5	-0.3	0.033
12	GO:0044464	CC	cell part	-2.5	-0.06	0.031
13	GO:0005623	CC	cell	-2.5	-0.06	0.031
14	GO:0043232	CC	intracellular non-membrane-bounded organelle	-2.5	-0.25	0.031
15	GO:0043228	CC	non-membrane-bounded organelle	-2.5	-0.25	0.031
16	GO:0043234	CC	protein complex	-2.9	-0.2	0.013
17	GO:0009570	CC	chloroplast stroma	-3	-0.3	0.011
18	GO:0009579	CC	thylakoid	-3.1	-0.29	0.0079
19	GO:0043231	CC	intracellular membrane-bounded organelle	-3.2	-0.095	0.0062
20	GO:0043227	CC	membrane-bounded organelle	-3.2	-0.095	0.0062
21	GO:0009532	CC	plastid stroma	-3.2	-0.3	0.0062
22	GO:0032991	CC	macromolecular complex	-3.4	-0.22	0.0032
23	GO:0043229	CC	intracellular organelle	-3.4	-0.1	0.0032
24	GO:0043226	CC	organelle	-3.4	-0.1	0.0032
25	GO:0005622	CC	intracellular	-3.6	-0.095	0.0023
26	GO:0005737	CC	cytoplasm	-3.6	-0.11	0.0023
27	GO:0044424	CC	intracellular part	-3.7	-0.099	0.0021
28	GO:0044444	CC	cytoplasmic part	-3.7	-0.11	0.0021
29	GO:0044434	CC	chloroplast part	-3.7	-0.25	0.0021
30	GO:0009507	CC	chloroplast	-3.8	-0.17	0.0018
31	GO:0044446	CC	intracellular organelle part	-3.9	-0.19	0.0014
32	GO:0044422	CC	organelle part	-3.9	-0.19	0.0014
33	GO:0009536	CC	plastid	-4.1	-0.17	0.0014
34	GO:0044435	CC	plastid part	-4.1	-0.26	0.0014

[a] BP = Biological Process; MF = Molecular Function; CC = Cellular Component.

Supplemental Table 7 - Parametric Analysis of Gene Expression - Enriched GO terms after SA treatment for combined genotypes

No	GO Term	Ontology[a]	Description	Z-score	Mean log2 FC	BY-adjusted p-value
1	GO:0009408	BP	response to heat	6.2	0.56	1.50E-07
2	GO:0009642	BP	response to light intensity	5.5	0.64	4.80E-06
3	GO:0006979	BP	response to oxidative stress	5.2	0.46	1.50E-05
4	GO:0042221	BP	response to chemical stimulus	5	0.21	2.90E-05
5	GO:0000302	BP	response to reactive oxygen species	4.8	0.61	6.70E-05
6	GO:0006950	BP	response to stress	4.4	0.18	0.00035
7	GO:0009314	BP	response to radiation	4.3	0.29	0.00039
8	GO:0010035	BP	response to inorganic substance	4.3	0.33	0.00044
9	GO:0009628	BP	response to abiotic stimulus	4.3	0.19	0.00044
10	GO:0009416	BP	response to light stimulus	4.2	0.29	0.00044
11	GO:0009266	BP	response to temperature stimulus	4.1	0.27	0.00069
12	GO:0050896	BP	response to stimulus	4	0.14	0.0012
13	GO:0006091	BP	generation of precursor metabolites and energy	3.9	0.38	0.0012
14	GO:0009415	BP	response to water	3.2	0.32	0.019
15	GO:0009414	BP	response to water deprivation	3.2	0.32	0.019
16	GO:0051171	BP	regulation of nitrogen compound metabolic process	3.1	0.18	0.021
17	GO:0045449	BP	regulation of transcription	3.1	0.18	0.021
18	GO:0031326	BP	regulation of cellular biosynthetic process	3.1	0.18	0.021
19	GO:0009889	BP	regulation of biosynthetic process	3.1	0.18	0.021
20	GO:0055114	BP	oxidation reduction	3.1	0.38	0.021
21	GO:0007154	BP	cell communication	3	0.32	0.021
22	GO:0031323	BP	regulation of cellular metabolic process	3	0.17	0.021
23	GO:0010556	BP	regulation of macromolecule biosynthetic process	3	0.18	0.023
24	GO:0080090	BP	regulation of primary metabolic process	3	0.17	0.024
25	GO:0006350	BP	transcription	2.9	0.17	0.025
26	GO:0019219	BP	regulation of nucleobase, nucleoside, nucleotide and nucleic acid metabolic process	2.9	0.17	0.026
27	GO:0010468	BP	regulation of gene expression	2.9	0.17	0.03
28	GO:0009605	BP	response to external stimulus	2.9	0.25	0.03
29	GO:0019222	BP	regulation of metabolic process	2.8	0.16	0.031
30	GO:0051716	BP	cellular response to stimulus	2.8	0.2	0.031
31	GO:0060255	BP	regulation of macromolecule metabolic process	2.7	0.16	0.042
32	GO:0051704	BP	multi-organism process	2.7	0.2	0.042
33	GO:0070887	BP	cellular response to chemical stimulus	2.7	0.23	0.042
34	GO:0051707	BP	response to other organism	2.7	0.22	0.042
35	GO:0050794	BP	regulation of cellular process	2.7	0.13	0.042
36	GO:0044085	BP	cellular component biogenesis	-2.6	-0.15	0.05

37	GO:0006412	BP	translation	-3.2	-0.14	0.02
38	GO:0003700	MF	transcription factor activity	4	0.22	0.0034
39	GO:0046906	MF	tetrapyrrole binding	3.9	0.48	0.0034
40	GO:0030528	MF	transcription regulator activity	3.8	0.2	0.0036
41	GO:0003677	MF	DNA binding	3.3	0.16	0.018
42	GO:0016651	MF	oxidoreductase activity, acting on NADH or NADPH	3.1	0.4	0.03
43	GO:0070469	CC	respiratory chain	3	0.38	0.05
44	GO:0031224	CC	intrinsic to membrane	-3	-0.24	0.05
45	GO:0043232	CC	intracellular non-membrane-bounded organelle	-3.2	-0.13	0.047
46	GO:0043228	CC	non-membrane-bounded organelle	-3.2	-0.13	0.047

[a] BP = Biological Process; MF = Molecular Function; CC = Cellular Component.

Supplemental Table 8 - Parametric Analysis of Gene Expression - Comparison of Basal Sca6 and ICS1 (Sca6 H_2O-ICS1 H_2O).

No	GO Term	Ontology[a]	Description	Z-score	Mean Log_2 FC	BY-adjusted p-value
1	GO:0042254	BP	ribosome biogenesis	3.1	0.53	0.026
2	GO:0022613	BP	ribonucleoprotein complex biogenesis	3.1	0.53	0.026
3	GO:0006412	BP	translation	2.9	0.24	0.044
4	GO:0006091	BP	generation of precursor metabolites and energy	-2.9	-0.49	0.042
5	GO:0006955	BP	immune response	-2.9	-0.64	0.042
6	GO:0002376	BP	immune system process	-2.9	-0.64	0.042
7	GO:0050789	BP	regulation of biological process	-3	-0.24	0.033
8	GO:0006952	BP	defense response	-3.1	-0.52	0.028
9	GO:0050794	BP	regulation of cellular process	-3.1	-0.26	0.023
10	GO:0051704	BP	multi-organism process	-3.2	-0.42	0.023
11	GO:0010200	BP	response to chitin	-3.2	-0.83	0.02
12	GO:0060255	BP	regulation of macromolecule metabolic process	-3.2	-0.33	0.019
13	GO:0010468	BP	regulation of gene expression	-3.3	-0.34	0.019
14	GO:0009743	BP	response to carbohydrate stimulus	-3.3	-0.6	0.015
15	GO:0006350	BP	transcription	-3.4	-0.35	0.013
16	GO:0009620	BP	response to fungus	-3.4	-0.85	0.013
17	GO:0080090	BP	regulation of primary metabolic process	-3.4	-0.36	0.013
18	GO:0019222	BP	regulation of metabolic process	-3.4	-0.34	0.013
19	GO:0031326	BP	regulation of cellular biosynthetic process	-3.5	-0.36	0.013
20	GO:0009889	BP	regulation of biosynthetic process	-3.5	-0.36	0.013
21	GO:0010556	BP	regulation of macromolecule biosynthetic process	-3.5	-0.37	0.013
22	GO:0051707	BP	response to other organism	-3.6	-0.53	0.013

23	GO:0045333	BP	cellular respiration	-3.6	-0.89	0.013
24	GO:0015980	BP	energy derivation by oxidation of organic compounds	-3.6	-0.89	0.013
25	GO:0009607	BP	response to biotic stimulus	-3.6	-0.52	0.013
26	GO:0045449	BP	regulation of transcription	-3.7	-0.39	0.013
27	GO:0019219	BP	regulation of nucleobase, nucleoside, nucleotide and nucleic acid metabolic process	-3.7	-0.39	0.013
28	GO:0031323	BP	regulation of cellular metabolic process	-3.8	-0.38	0.013
29	GO:0051171	BP	regulation of nitrogen compound metabolic process	-3.9	-0.39	0.013
30	GO:0030528	MF	transcription regulator activity	-3.5	-0.34	0.029
31	GO:0003700	MF	transcription factor activity	-3.6	-0.37	0.029
32	GO:0022626	CC	cytosolic ribosome	4.7	0.64	0.00022
33	GO:0043232	CC	intracellular non-membrane-bounded organelle	4	0.31	0.0017
34	GO:0043228	CC	non-membrane-bounded organelle	4	0.31	0.0017
35	GO:0030529	CC	ribonucleoprotein complex	3.8	0.36	0.0026
36	GO:0005840	CC	ribosome	3.8	0.4	0.0026
37	GO:0022625	CC	cytosolic large ribosomal subunit	3.6	0.71	0.0052
38	GO:0044445	CC	cytosolic part	3.5	0.47	0.0062
39	GO:0070469	CC	respiratory chain	-3	-0.66	0.037

[a] BP = Biological Process; MF = Molecular Function; CC = Cellular Component.

Supplemental Table 9 - Parametric Analysis of Gene Expression - Comparison of Induced Sca6 and ICS1 (Sca6 SA-ICS1 SA)

No	GO Term	Ontology[a]	Description	Z-score	Mean Log_2 FC	BY-adjusted p-value
1	GO:0019684	BP	photosynthesis, light reaction	3.6	0.7	0.027
2	GO:0015979	BP	photosynthesis	3.6	0.55	0.027
3	GO:0022607	BP	cellular component assembly	3.2	0.52	0.04
4	GO:0034621	BP	cellular macromolecular complex subunit organization	3.2	0.59	0.04
5	GO:0034622	BP	cellular macromolecular complex assembly	3.1	0.6	0.048
6	GO:0043933	BP	macromolecular complex subunit organization	3.1	0.51	0.048
7	GO:0051707	BP	response to other organism	-3	-0.44	0.048
8	GO:0009607	BP	response to biotic stimulus	-3.1	-0.44	0.048
9	GO:0010033	BP	response to organic substance	-3.2	-0.32	0.04
10	GO:0009743	BP	response to carbohydrate stimulus	-3.3	-0.63	0.04
11	GO:0009605	BP	response to external stimulus	-3.3	-0.53	0.04
12	GO:0009620	BP	response to fungus	-3.4	-0.84	0.039
13	GO:0009611	BP	response to wounding	-3.6	-0.72	0.027
14	GO:0004553	MF	hydrolase activity, hydrolyzing O-glycosyl compounds	-3.7	-0.75	0.013
15	GO:0030246	MF	carbohydrate binding	-3.8	-1	0.013
16	GO:0044435	CC	plastid part	5.5	0.47	4.20E-06

17	GO:0044434	CC	chloroplast part	5.2	0.47	9.80E-06
18	GO:0009507	CC	chloroplast	5	0.27	1.70E-05
19	GO:0009536	CC	plastid	4.8	0.25	4.40E-05
20	GO:0044446	CC	intracellular organelle part	4.4	0.24	0.00016
21	GO:0044422	CC	organelle part	4.4	0.24	0.00016
22	GO:0034357	CC	photosynthetic membrane	4	0.59	0.00076
23	GO:0005737	CC	cytoplasm	4	0.14	0.00087
24	GO:0009532	CC	plastid stroma	3.9	0.54	0.001
25	GO:0005622	CC	intracellular	3.8	0.11	0.0012
26	GO:0009570	CC	chloroplast stroma	3.7	0.56	0.0019
27	GO:0009579	CC	thylakoid	3.6	0.44	0.0019
28	GO:0044424	CC	intracellular part	3.6	0.11	0.0019
29	GO:0044444	CC	cytoplasmic part	3.5	0.13	0.0026
30	GO:0043229	CC	intracellular organelle	3.4	0.1	0.004
31	GO:0043226	CC	organelle	3.4	0.1	0.004
32	GO:0055035	CC	plastid thylakoid membrane	3.3	0.56	0.0041
33	GO:0009535	CC	chloroplast thylakoid membrane	3.3	0.56	0.0041
34	GO:0032991	CC	macromolecular complex	3.2	0.2	0.006
35	GO:0009521	CC	photosystem	3.2	0.84	0.0066
36	GO:0010287	CC	plastoglobule	3.2	0.69	0.0066
37	GO:0044436	CC	thylakoid part	3.1	0.47	0.0072
38	GO:0031984	CC	organelle subcompartment	3	0.44	0.0087
39	GO:0031976	CC	plastid thylakoid	3	0.44	0.0087
40	GO:0009534	CC	chloroplast thylakoid	3	0.44	0.0087
41	GO:0043231	CC	intracellular membrane-bounded organelle	3	0.094	0.0092
42	GO:0009526	CC	plastid envelope	3	0.4	0.0094
43	GO:0042651	CC	thylakoid membrane	3	0.47	0.0095
44	GO:0043227	CC	membrane-bounded organelle	2.9	0.092	0.01
45	GO:0044464	CC	cell part	2.7	0.062	0.018
46	GO:0005623	CC	cell	2.7	0.062	0.018
47	GO:0043232	CC	intracellular non-membrane-bounded organelle	2.5	0.24	0.033
48	GO:0043228	CC	non-membrane-bounded organelle	2.5	0.24	0.033
49	GO:0044430	CC	cytoskeletal part	2.5	0.62	0.035
50	GO:0009941	CC	chloroplast envelope	2.4	0.37	0.041
51	GO:0030312	CC	external encapsulating structure	-2.4	-0.39	0.041
52	GO:0005618	CC	cell wall	-2.4	-0.39	0.041

[a] BP = Biological Process; MF = Molecular Function; CC = Cellular Component.

Supplemental Table 10 - qRT-PCR primers

Annotation	TcID	Sequence
Actin	Tc10_g003310	5'-TTCTGGTGCAGCTTGGAACTTTGC-3'<br>5' - AGTTCATCGTCACTCCAACATGAGAACA - 3'
Photosystem II CP47 chlorophyll apoprotein	TC04_g003810	5'- GCTAGAACCCTGTTCAGAGATG -3'<br>5'- GACTACTTGTCTTCTTGTAGTTGGA -3'
Peroxidase 4	TC09_g034930	5'- ATTAAGGCCAAGGTGGAGAAAG -3'<br>5'- AGTCAGGTCCTCCAAGACAA -3'
Endochitinase 1	Tc02_g003890	5'-TTTCTCACGCACAGACAGTAGCGA-3’<br>5’-TTGTAGCGAAGGCAGGGAAAGACT-3’
Photosystem I P700 chlorophyll a apoprotein A1	Tc04_g003910	5'- CAA TAG CTA AGG GAC CCG ATA C -3'<br>5'- CGA GAG ATC TCC TCC AAA TCA C -3'
NADH-ubiquinone oxidoreductase chain 4L	Tc00_g007720	5'- GAT CTC TCA CGA GGC CAT AAA T -3'<br>5'- CGA CGT ATT TCC GAT CCT TGA -3'
NADH-ubiquinone oxidoreductase chain 1	TC05_g000740	5'- TAC AGG GCT AGG GCT CAT AA -3'<br>5'- CTG AGG TGC GAA GAG AAA CA -3'
Photosystem II CP43 chlorophyll apoprotein	Tc04_g003900	5'- GAA CAT GGC CAT ATG AGT TGA TAC -3'<br>5'- CTA ATC CTC CAA GCT ACC AAC A -3'
Acidic endochitinase	TC01_g035050	5'- CTA CGT CCT TTC TTC ACC AGA G -3'<br>5'- TGC CAT CTA AGA CAG CGT TAC -3'
Endochitinase	Tc01_g000770	5'- CTG GAA ACG CCA TTA GAG AAG A -3'<br>5'- GCT GTC TTG AAG GAT ATG ATT GC -3'
Peroxidase 43	Tc08_g012700	5'- GAC AGA GAG ACC AGG CAA ATA C -3'<br>5'- TCC CAT CTT CAC CAT TGC TTT A -3'
Glucan endo-1,3-beta-glucosidase, basic vacuolar isoform	Tc04_g029300	5'- CCT TGC TAA CCC TTC CAA TGC ACA G -3'<br>5'- CCA AGG CAG GCA AAA CAA ATT GAG C -3'
